# Carbene-Catalyzed Phthalide Ether Functionalization for Discovering Chiral Phytovirucide that Specifically Targets Viral Nia Protein to Inhibit Proliferation

**DOI:** 10.34133/research.0637

**Published:** 2025-03-14

**Authors:** Xiaoyi Wang, Weijia Yang, Shang Wu, Fangru Jin, Zhongjie Shen, Xiangyang Li, Yonggui Robin Chi, Baoan Song, Runjiang Song

**Affiliations:** ^1^ State Key Laboratory of Green Pesticide, Center for R&D of Fine Chemicals of Guizhou University, Guiyang 550025, P. R. China.; ^2^ School of Chemistry, Chemical Engineering, and Biotechnology, Nanyang Technological University, Singapore 637371, Singapore.

## Abstract

Plant diseases caused by vegetable viruses are an important threat to global food security, presenting a major challenge for the development of antiviral agrochemicals. Functional proteins of plant viruses play a crucial role in the viral life cycle, and targeted inhibition of these proteins has emerged as a promising strategy. However, the current discovery of antiviral small molecules is hampered by the limitations of synthetic approaches and the narrow range of targets. Herein, we report a practical application of organocatalysis for serving pesticide discovery that bears a unique molecular basis. An *N*-heterocyclic carbene-modulated reaction is first designed to asymmetrically functionalize diverse natural phenols with phthalides. Our designed method is capable of producing a series of new phthalidyl ethers under mild conditions with good yields, enantioselectivity, and functional group tolerance. Among these, compound (*R*)-**3w** exhibits excellent and enantioselectivity-preferred curative activity against potato virus Y (PVY). Mechanistically, it is proposed that (*R*)-**3w** interacts with the nuclear inclusion body A (Nia) protein of PVY at the His150 residue. This binding impairs Nia’s function to cleavage polyprotein, thereby inhibiting formation of viral replication complex. The study provides insights into advancing synthetic protocol to facilitate agrochemical discovery, and our identified (*R*)-**3w** may serve as a potential lead for future research and development PVY-Nia inhibitors.

## Introduction

Currently, the global population continues to grow, with projections indicating that by 2050, there will be nearly 10 billion people requiring sustenance [1–4]. In addition, the unpredictability of weather patterns can lead to crop failures, reduced agricultural land productivity, and increased vulnerability to pests and diseases [5–7]. The intricate tapestry of global food security is increasingly being strained by the dual challenges of climate change and a burgeoning population. In the face of these issues, ensuring food security while adhering to the principles of sustainable development is a formidable task, which necessitates the urgent development of sustainable plant protection strategies [8,9]. “Green” pesticides are characterized not only by their high efficacy in pest control and management, but also by their safety for nontarget organisms and humans. Meanwhile, the process of preparing these pesticides must adhere to principles of sustainability [10–12]. Asymmetric catalysis has emerged as a potent tool for discovering or manufacturing useful molecules with stereoselectivity-driven bioactivities, such as asymmetric hydrogenation to Ramipril and Montelukast [13,14]. Within this field, asymmetric synthesis enabled by organocatalysis, owing to its advantages of simple operation, mild conditions, metal-free nature, and relative sustainability, holds promise for the development of green agrochemicals [15–17]. Despite the success of organocatalysis in pharmaceutical synthesis and polymer preparation and degradation, its application in pesticide discovery is still limited [18–22].

Potato virus Y (PVY) is a significant plant pathogen belonging to the *Potyvirus* genus within the family *Potyviridae*. It is widely recognized as one of the most economically detrimental viruses affecting potato crops (*Solanum tuberosum*) and other solanaceous plants, including peppers and tomatoes [23–27]. PVY management has remained a pending problem in agricultural chemistry, stemming from the evolutionary resistance of its vector insect, the aphid, to insecticides, as well as the repetitive molecular targets of existing antiviral agents [28–30]. Based on the genome of PVY, it encodes a total of 10 functional proteins, including the coat protein (CP), nuclear inclusion body A (Nia) protein, the first protein, helper component-proteinase, etc. [31]. These proteins play crucial roles in the PVY life cycle, pathogenic behavior, and virulence. Interfering with these key viral proteins using small molecules has emerged as a promising antiviral strategy. Indeed, a number of potential small molecules focusing on PVY CP have been progressively uncovered [32–34]. For instance, our previous work on *N*-heterocyclic carbene (NHC)-mediated [3+4] cycloaddition discovered a chiral antiviral compound with a unique molecular structure of arylimidazole-fused diazepine and mechanism that competitively inhibits the interaction between CP and plant host factor, thereby disabling the virion’s ability to move across cells [35]. Additionally, the commercially available drug ningnanmycin has been suggested to exert its antiviral effects by binding to CP [33,36]. However, though CP is important for protecting the PVY genome and facilitating intercellular traffic, antivirals with a similar mechanism of action are susceptible to resistance induced by point mutations [37–39]. Therefore, it is worthwhile to investigate potential small molecules beyond serving inhibitors of CP. Unfortunately, to our knowledge, compounds targeting other functional proteins of PVY have yet to be identified.

**Fig. 1.**
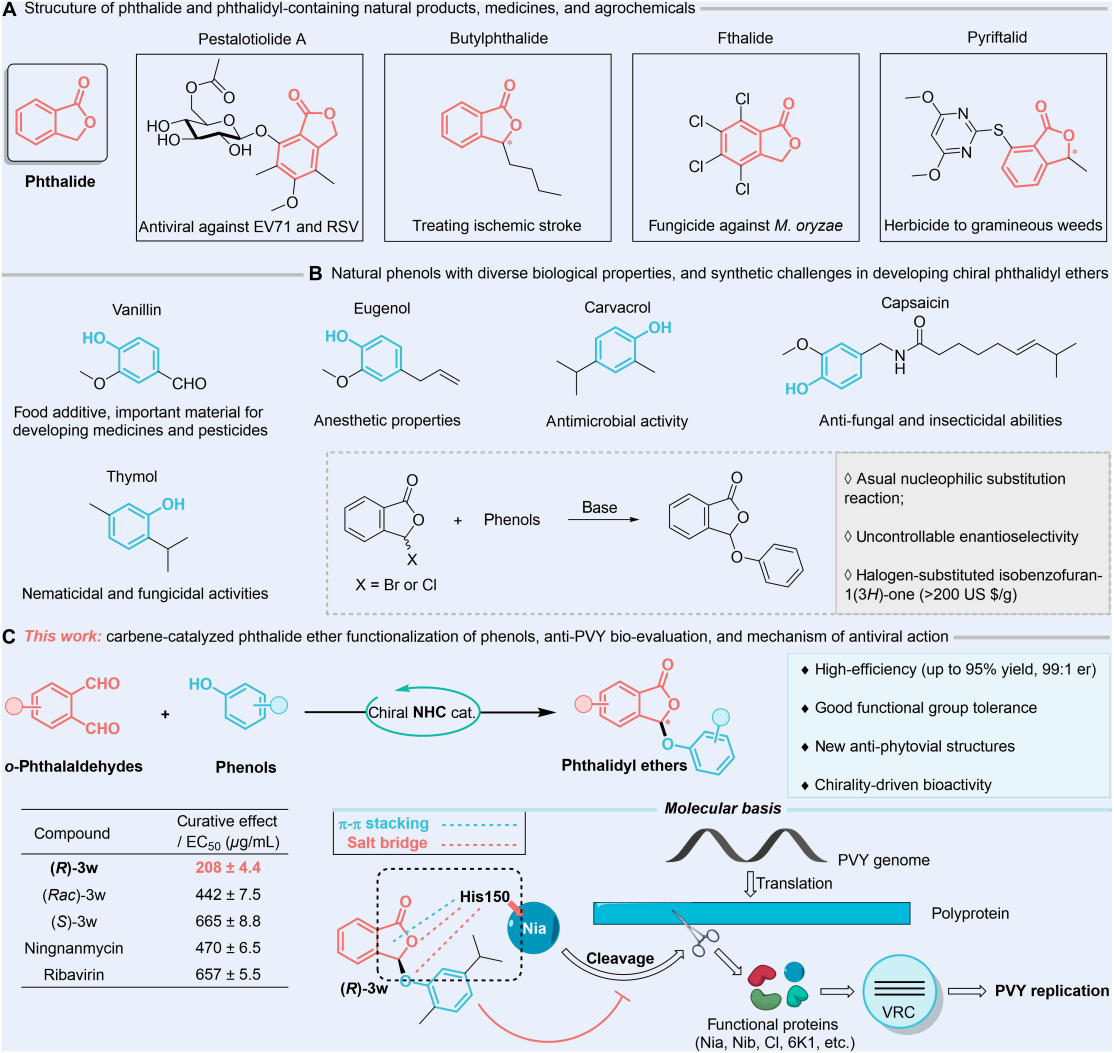
Discovery of phthalide antivirals with unique mechanisms of action. (A) Strucuture of phthalide and phthalidyl-containing natural products, medicines, and agrochemicals. (B) Natural phenols with diverse biological properties and synthetic challenges in developing chiral phthalidyl ethers. (C) Carbene-catalyzed phthalide ether functionalization of phenols, anti-PVY bio-evaluation, and mechanism of antiviral action.

In our pursuit to identify new molecules with potential anti-PVY activity, phthalides have garnered significant attention. These derivatives possess a wide range of biological properties and have been explored for treating ischemic stroke and acute coughing, as well as for their antifungal and herbicidal applications (Fig. 1A) [40–42]. Furthermore, phthalide esters are commonly employed as prodrug moieties in pharmaceutical design. In contrast, there is a paucity of studies concerning their antiviral properties. Jia et al. [43] were the first to isolate a unique phthalidyl ether, pestalotiolide A, from the fermentation broth of *Pestalotiopsis* sp., which exhibited an inhibitory impact against enterovirus 71 and respiratory syncytial virus. This indicates that phthalide ethers may constitute promising leads for developing phytovirucides with innovative mechanisms of action. Furthermore, many natural phenols possess diverse structures and pesticidal activities and are available at a low market price. In terms of antimicrobial properties, thymol has already been registered as a pesticide [44–46]. Consequently, natural phenols can serve as promising fragments for the construction of ether bonds with phthalides. The 3-position of phthalides is the primary site for derivatization, yet challenges persist, especially in the asymmetric introduction of active groups at this site, which could enhance the specific binding of molecules to targets and reduce the risk to nontarget organisms. Conventional methods involve the substitution reaction of phenols with 3-halogenoisobenzofuran-1(3*H*)-one that is constrained in the discovery of pesticides due to the inability to control enantioselectivity and the high cost of initial materials (Fig. 1B).

Herein, we report a carbene-catalyzed phthalide ether functionalization of phenol compounds, facilitating facile synthesis of a variety of optically enriched aryl phthalidyl ethers (Fig. 1C). A myriad of differently substituted phenols, even natural phenols, can be converted into their corresponding products with outstanding enantioselectivity (up to 99:1 er) and conversion rates (up to 95% yield) under our optimal condition catalyzed by NHC. We further discovered that the phthalide ether derivative (*R*)-**3w** of carvacrol possesses potent activity against PVY through bio-evaluations. Specifically, it displayed substantially superior and enantioselectivity-preferred curative effects in comparison to the popular commercial drugs ningnanmycin and ribavirin, as well as its enantiomer (*S*)-**3w** and racemic mixture (*Rac*)-**3w**, which encouraged subsequent mechanism of action investigations. It is noteworthy that the curative effect of a drug usually correlates with its impact on pathogen replications, and the current mechanisms of antiviral action in plants are primarily based on the protective and inactivating activities of virucides [47]. Combining molecular docking with a series of molecular biological validations allows one to reasonably hypothesize that (*R*)-**3w** may act on a brand-new molecular target via His150 residue, namely, the Nia involved in cleavage of the viral polyprotein into functional proteins. Several of these proteins are essential for generation of the viral replication complex (VRC), which mediates PVY replication. Both the mutation at site 150 and (*R*)-**3w** treatment significantly reduced the proliferation of PVY in *Nicotiana benthamiana (N. benthamiana*). We expect this study to contribute novel antiviral structures, asymmetric synthetic protocol, and potential new target protein for the PVY management.

## Results and Discussion

### Synthetic methodology

**Fig. 2.**
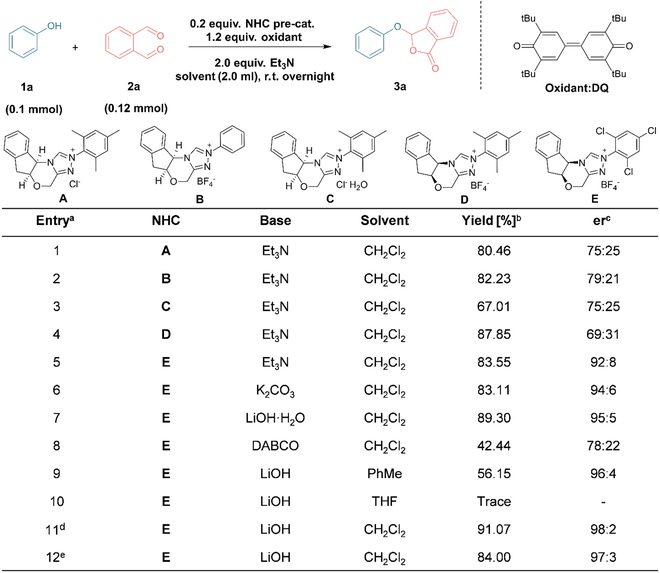
Optimization of reaction conditions ^a^ Unless otherwise specified, the reactions were carried out with 1a (0.1 mmol), 2a (0.12 mmol), NHC (20 mol%), base (200 mol%), DQ (120 mol%), CH_2_Cl_2_ (2 ml), 12 h, r.t. ^b^ Isolated yield. ^c^ Determined by chiral HPLC analysis (IB column, 0.6 ml/min, hexane/iPrOH = 80/20). ^d^ Determined by chiral HPLC analysis (IB column, 0.6 ml/min, hexane/iPrOH = 80/20); 1a (0.1 mmol), 2a (0.12 mmol), NHC (20 mol%), DQ (120 mol%), base (200 mol%), 4Å MS (80 mg), solvent (2 ml), 12 h, r.t. ^e^ 1a (0.1 mmol), 2a (0.12 mmol), NHC (20 mol%), DQ (120 mol%), base (200 mol%), solvent (2 ml), 12 h, 0 °C.

Our initial attempt involved the use of phenol (**1a**) and aldehyde (**2a**) as standard substrates under the catalysis of NHC precursor **A** [48] and the mediation of oxidant quinone (DQ). These starting materials are commercially available and inexpensive. The primary results obtained are presented in Fig. 2. Encouragingly, a decent yield was achieved, and the title product **3a** was observed to be nonracemic (Fig. 2, entry 1). Under the same conditions, modifying the *N*-mesityl group of the catalyst to an unsubstituted phenyl group (to produce catalyst **B** [49]) slightly enhanced both the yield and the optical purity of the product (entry 2). In contrast, the use of the hydrated form of **A**, namely, catalyst **C** [50], led to a decrease in yield (entry 3). Considering that the different anion might also influence reactivity [51], we replaced pre-catalyst **A** with its tetrafluoroborate form **D** [52], but it did not yield satisfactory results (entry 4). Based on previous studies suggesting the reversible addition of the NHC to aldehydes regulated by the *N*-substituent of the catalyst, which affects the formation of the Breslow intermediate [53], we further introduced electron-withdrawing groups into the *N*-aryl ring. With the assistance of triazole salt **E** [54], the conversion of the reaction could be maintained above 80% while significantly enhancing the enantioselectivity (entry 5). Moreover, the reaction could proceed with either organic or inorganic bases, but the latter generally provided better stereoselectivity in this case (entries 6 to 8). The choice of solvent had a pronounced impact on the reaction, with solvents other than dichloromethane yielding adverse effects on the reaction outcomes (entries 9 to 11). Additionally, conducting the reaction at lower temperatures, such as 0 °C, did not further enhance the optical purity of the product while also reducing the yield (entry 12). Overall, we favor the combination of entry 11 as the optimal condition for the reaction (for the detailed optimization process, please refer to Table S1).

**Fig. 3.**
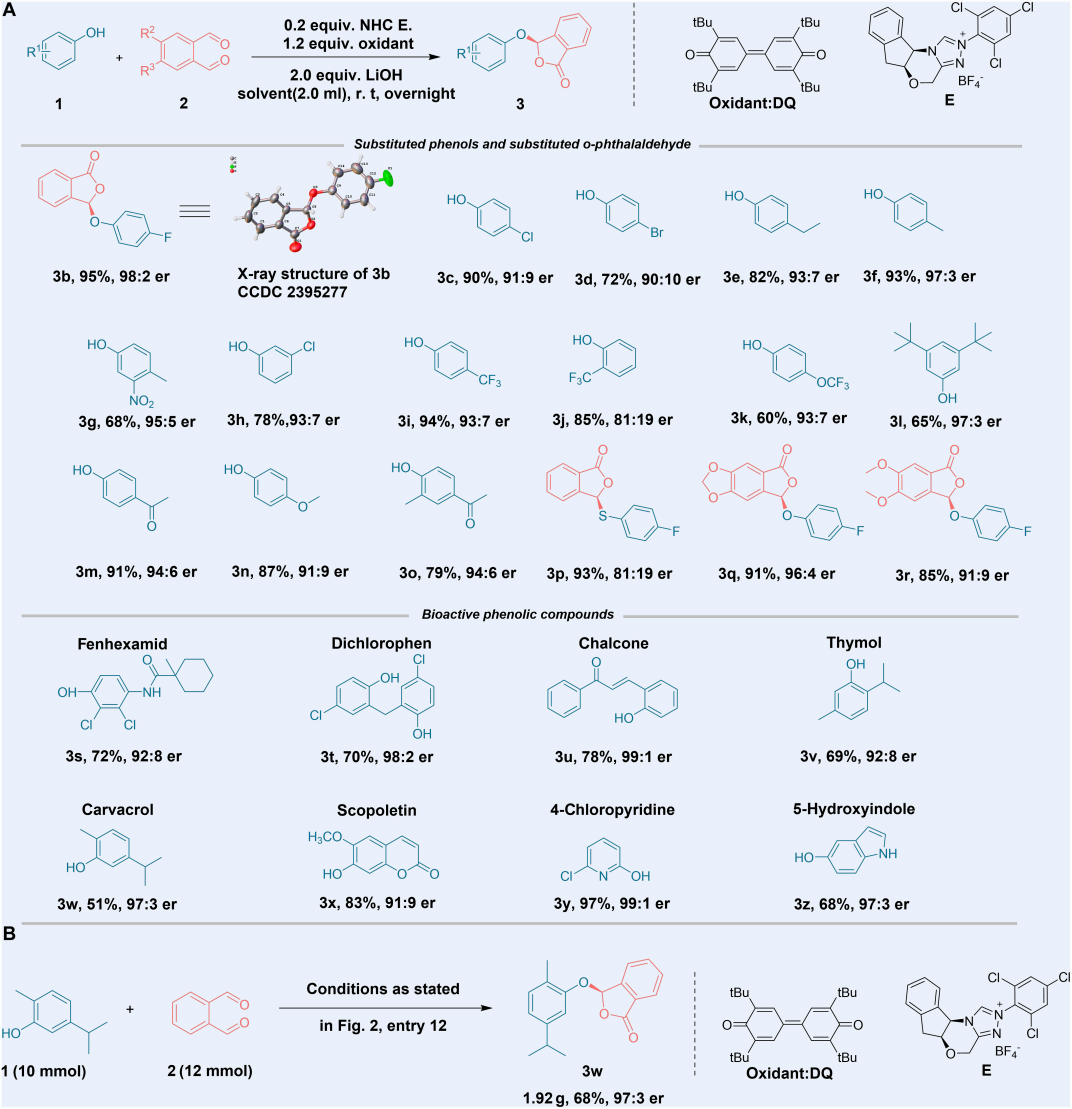
(A) Reaction scope of phenolic compound 1 and ortho benzaldehyde substrate 2. The reaction conditions are shown in Fig. 2, entry 12. The yield is the isolated yield after purification by column chromatography. The Er value is determined by HPLC on a chiral stationary phase. (B) Gram-scale preparation of (*R*)-3w using our developed catalysis.

Armed with the optimized reaction conditions, we next explored the scope of the reaction using various phenols **1** and substituted *o*-phthalaldehydes **2**. As shown in Fig. 3A, the reaction tolerated phenols with electron-withdrawing and electron-donating groups, as well as multiply substituted species, affording relatively high yields and enantiomeric ratios (**3b** to **3r**). Bioisosteric replacement is a widely used strategy in drug design; therefore, we attempted to change phenol with its bioisosteres thiophenol upon the same condition. The reaction proceeded successfully with thiophenol, producing the corresponding product in good yield with slightly reduced enantioselectivity (**3p**). Furthermore, the use of substituted *o*-phthalaldehydes also led to the corresponding derivatives without a significant decrease in er values. Notably, phenolic structures are common in natural products, pesticides, and pharmaceuticals, exhibiting a broad range of biological activities [55–57]. For example, fenhexamid, a commercialized fungicide developed by Bayer, is primarily used to control plant diseases such as gray mold, sclerotinia, and black spot [58]. Thymol, another phenolic compound, is registered as an active component in pesticide products utilized as an animal repellant, microbicide, disinfectant, and anti-tubercular agent [44,45]. To our delight, these structurally unique phenolic ingredients demonstrated good functional group tolerance in the reaction, providing phthalide-functionalized products with high optical purity (Fig. 3A, **3****s** to **3****z**). This phthalide ether functionalization reaction was preliminary assessed at the gram scale using **3w** as an example (Fig. 3B). Upon increasing the reaction conditions, including the quantities of substrate, catalyst, and oxidant, by a factor of 100, the conversion rate enhanced while the enantioselectivity remained consistent.

**Fig. 4.**
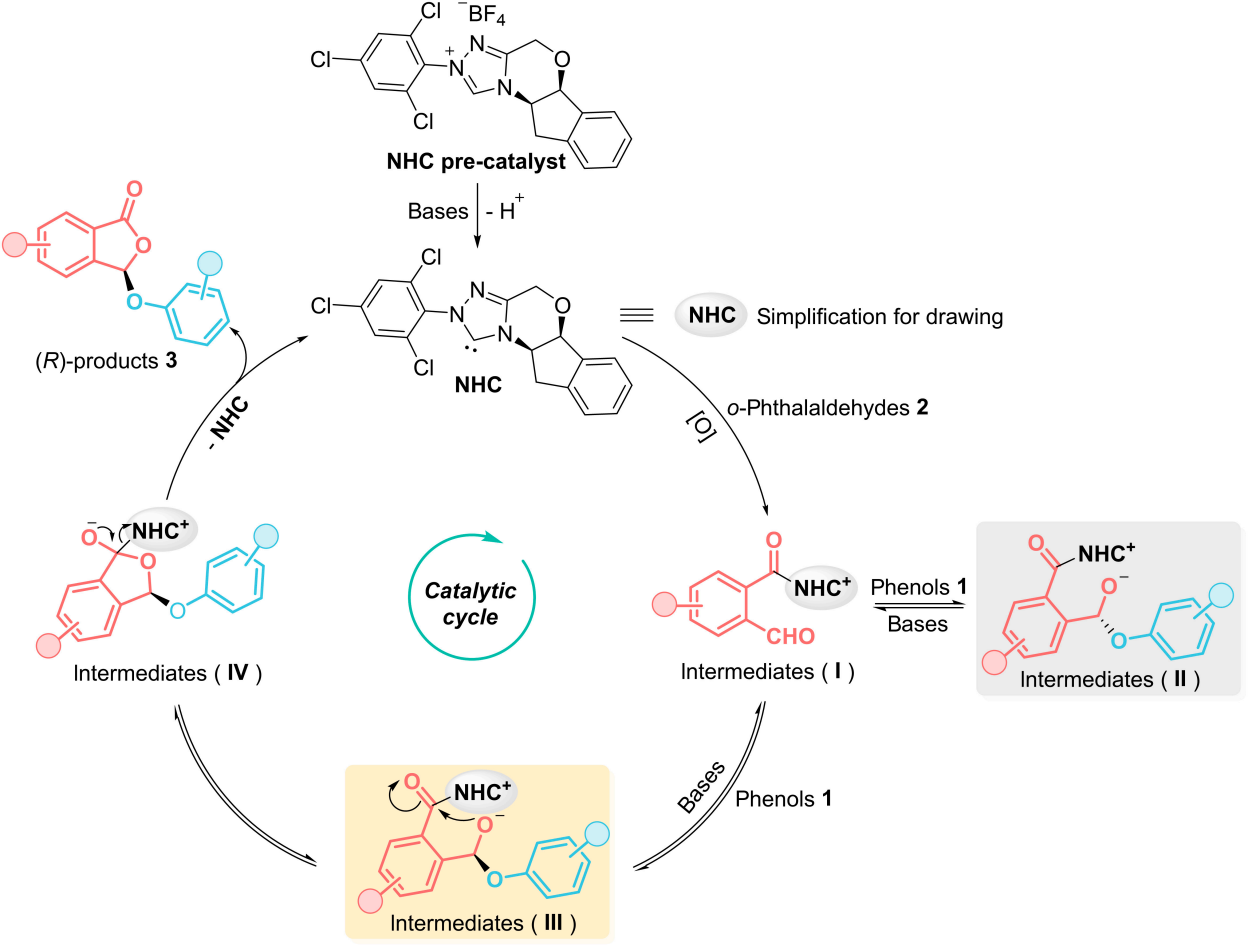
Speculated mechanism of our developed NHC-catalyzed phthalide ether functionalization of phenols.

To elucidate the mechanistic underpinnings of our synthetic methodology, we proposed a preliminary catalytic cycle as depicted in Fig. 4. The inferred reaction pathway involves the following sequence: The NHC precatalyst undergoes deprotonation under basic conditions to generate a catalytically active free NHC species. This species subsequently reacts with phthalaldehydes **2** in the presence of an oxidant, forming NHC-bound intermediates **I** [59]. Intermediates **I** then engage with phenolic substrates **1** via nucleophilic attack at the carbonyl group, yielding 2 stereochemically distinct intermediates (**II** and **III**), contingent upon the trajectory of nucleophilic addition (upper or lower face of the carbonyl moiety). Although this step is theoretically reversible, the steric bias imparted by the chiral NHC framework enforces a kinetic preference for the formation of intermediates **III**. Subsequent addition–elimination steps generate intermediates **IV**, which releases the free NHC to reinitiate the catalytic cycle while affording the target products **3**. Recently, carbene-mediated reactions have emerged as a powerful tool for stereoselective optimization of hydroxyl-containing bioactive constituents (e.g., saccharides, carboxylic acids, aldehydes, and alcohols) in drugs and natural products, yet their application in phenolic compound modification remains underexplored [60–64]. Beyond advancing synthetic methodologies, the characterization of novel types of chirality may facilitate the discovery of functional molecules with exciting properties [65–68]. To sum up, our synthetic protocol can efficiently achieve functionalization and modification of complex phenols with different substituting patterns or even natural ones with good stereoselectivity and scalability, which is expected to serve the discovery of medicines or pesticides.

### Anti-PVY evaluations

PVY-induced viral diseases have a detrimental impact on the sustainable yield of solanaceous crops. Current antiviral strategies mainly focus on managing the vector aphids, using specific insecticides such as pymetrozine or neonicotinoids [69]. However, the repertoire of direct antiviral agents remains relatively limited. Many phenolic and phthalide derivatives are known to possess significant antimicrobial potential [44–46,55–58]. To thoroughly evaluate the practical utility of our developed synthetic method in plant protection, we systematically assessed the antiviral activity of all synthesized products against PVY (Tables 1 and 2), using commercially available antiviral agents ningnanmycin and ribavirin as positive controls. Several of our synthesized isobenzofuran-1(3*H*)-one derivatives exhibited promising antiviral activity, with compound (*R*)-**3w** demonstrating superior curative efficacy compared to 2 commercial antivirals. We also tested the anti-PVY activity of carvacrol, which demonstrated limited efficacy. This observation precludes the possibility of intrinsic antiviral activity in carvacrol itself and confirms that structural modification with phthalide significantly enhances its bioactivity against PVY. Preliminary structure–activity relationship analysis based on anti-PVY activity indicated that most chiral isobenzofuranone derivatives synthesized from phenolic compounds with natural bioactivity exhibited moderate to excellent protective, curative, and inactive effect against PVY (e.g., **3s**, **3v**, **3w**, and **3y**). Compared with the commercial reference drugs ningnanmycin and ribavirin, compound **3v** derived from the thymol isomer of carvacrol exhibited appreciable antiviral activity against PVY, although its efficacy was lower than that of compound (*R*)-**3w**, which also demonstrated excellent curative activity against PVY. This suggests that the steric hindrance of the substituents may be a critical factor influencing antiviral activity. Compounds **3b**, **3c**, and **3d**, with identical electron-withdrawing groups (such as -F, -Cl, and -Br), exhibited similar curative, protective, and inactivating activities against PVY. Substituting phenol with thiophenol (e.g., **3p**) enhanced the inactivating activity against PVY. Additionally, compounds **3q** and **3r** further illustrated the significant impact of the steric hindrance of substituents on the antiviral activity.

**Table 1.** Anti-PVY activities of the compounds 3b-3z(*R*) (500 μg/ml)

Compound^a^	Curative effect (%)	Protective effect (%)	Inactive effect (%)
3b	52.37 ± 0.61	42.69 ± 3.50	52.28 ± 3.52
3c	54.95 ± 3.27	45.17 ± 2.52	51.28 ± 3.88
3d	38.58 ± 4.10	39.21 ± 1.36	42.56 ± 0.89
3e	30.82 ± 4.65	43.73 ± 4.28	40.08 ± 1.71
3f	49.82 ± 4.78	52.90 ± 4.48	49.75 ± 1.31
3g	58.00 ± 4.46	31.22 ± 3.47	52.26 ± 0.76
3h	36.88 ± 2.86	32.52 ± 2.44	55.04 ± 4.92
3i	43.67 ± 3.44	59.82 ± 3.04	52.83 ± 3.61
3j	43.65 ± 4.66	51.17 ± 2.49	45.10 ± 3.58
3k	47.53 ± 7.79	37.68 ± 3.73	55.84 ± 1.86
3l	30.63 ± 4.99	31.15 ± 4.13	47.13 ± 3.58
3m	40.49 ± 2.13	57.46 ± 0.85	40.28 ± 3.65
3n	42.45 ± 3.50	46.02 ± 3.30	38.82 ± 1.43
3o	39.61 ± 1.29	44.54 ± 3.15	39.12 ± 1.11
3p	31.00 ± 4.52	42.15 ± 3.37	63.50 ± 3.46
3q	54.88 ± 4.54	55.57 ± 2.60	52.64 ± 1.81
3r	50.44 ± 1.32	54.45 ± 3.47	51.66 ± 4.28
3s	34.90 ± 2.49	34.05 ± 3.50	57.23 ± 4.55
3t	33.87 ± 2.23	47.47 ± 2.62	41.88 ± 0.86
3u	46.29 ± 1.91	47.45 ± 2.57	40.33 ± 1.95
3v	54.26 ± 0.61	45.52 ± 2.60	55.45 ± 4.96
3w	67.30 ± 1.30	46.95 ± 4.46	61.16 ± 3.99
3x	37.37 ± 4.84	48.68 ± 2.59	44.23 ± 2.07
3y	30.90 ± 3.98	30.90 ± 3.98	52.76 ± 2.15
3z	40.20 ± 4.48	47.77 ± 3.17	40.20 ± 4.48
Carvacrol	31.81 ± 1.41	36.00 ± 2.80	44.15 ± 0.72
NNM^b^	50.69 ± 3.38	51.6 ± 3.02	85.9 ± 2.34
RBV^c^	45.04 ± 3.61	51.5 ± 4.89	62.6 ± 2.22

^a^ All data were average data of 3 replicates. Ribavirin was used as the positive control.

^b^ NNM, ningnanmycin.

^c^ RBV, ribavirin.

**Table 2.** EC_50_ values of the (*R*)-3w, (*Rac*)-3w, and (*S*)-3w anti-PVY in vivo (μg/ml)

Compound^a^	Curative effect (%)	Curative effect (EC_50_)
(*R*-3w	67.30 ± 1.30	208 ± 4.4
(*Rac*)-3w	50.35 ± 3.51	442 ± 7.5
(*S*)-3w	40.57 ± 3.18	665 ± 8.8
NNM^b^	50.69 ± 3.38	470 ± 6.5
RBV^c^	45.04 ± 3.61	657 ± 5.5

^a^ All data were average data of 3 replicates. ribavirin was used as the positive control.

^b^ NNM, ningnanmycin.

^c^ RBV, ribavirin.

Chiral pesticides have emerged as potent tools for achieving selective control of agricultural pests. Enantiomers within a pair can display significantly different performances in pest control, toxicity to nontarget organisms, and even distinct fates in the environment [70,71]. A representative instance is metolachlor, a widely used herbicide effective against grass and broadleaf weeds. However, *S*-metolachlor has been demonstrated to possess better herbicidal activity and bio-safety compared to its *R*-enantiomer [72,73]. On this basis, we selected the (*R*)-**3w** identified in our previous bio-screening for further investigation. With assistance from the enantiomer of pre-catalyst **E** and an achiral NHC catalyst, we successfully synthesized the *S*-enantiomer [(*S*)-**3w**] and the racemic mixture (*Rac*)-**3w** of (*R*)-**3w**. By further comparing their protective activities against PVY, we were surprised to find that the *R* isomer had a significantly lower EC_50_ value than its enantiomer and racemate. The results of the biological evaluation provide more evidence that supports the great potential of our synthetic approach in pesticide discovery. Compound (*R*)-**3w** with a novel structure may behave as an efficient chiral phytoviricide, and it is worth exploring whether it has a unique mechanism of action.

### Mechanism of antiphytoviral action

Currently, there are 2 main explanations for the mechanisms of action of antiviral agrochemicals. The first is drugs serving as plant immune inducers in preventing and controlling viral infections by activating the plant’s innate immune responses [74]. These inducers trigger the production of signaling molecules such as salicylic acid, jasmonic acid, and ethylene [75]. Upon activation, these signaling molecules induce the expression of defense-related genes, including those encoding pathogenesis-related (PR) proteins, which have antiviral properties [76]. PR proteins can directly interfere with viral replication and movement within the plant, as well as enhance the plant’s structural and biochemical defenses. Additionally, immune inducers can activate systemic acquired resistance, a long-lasting and broad-spectrum resistance mechanism that primes the plant to more effectively defend against future viral attacks [75]. In most cases, these pesticides exhibit favorable protective activity. Another straightforward basis is inactivation of viral replication, motility and infection, which can be realized through molecules that target the viral RNA or proteins [77–80]. Recently, numerous examples of using small-molecule inhibitors to inactivate the PVY CP have emerged, and despite sharing the same target, their mechanisms of action are not identical [81–83]. For instance, benzo[*b*]thiophene and 2*H*-chromene-based analogues may interfere with viral particle formation by competing for the binding sites of CP to nucleic acids [84], whereas arylimidazole-fused diazepines have been reported to disrupt the interaction between CP and host pro-viral factors, thereby affecting the intercellular movement of viral particles [85]. These studies are often based on the superior inactivation activity of small molecules. In contrast, our compound has shown curative effects in tests with infected plants. Typically, during the early stages of infection, the drug may achieve its purpose by interfering with the efficient proliferation of the virus. PVY proliferation depends on the proper execution of genomic replication functions, which requires the coordinated regulation of several functional proteins. Among them, Nia acts as a critical scissor, responsible for cleaving the polyprotein translated from the genome into essential functional proteins necessary for subsequent self-reproduction [86,87]. Therefore, we propose that Nia might be the primary target of (*R*)-**3w** and aim to verify this hypothesis.

**Fig. 5.**
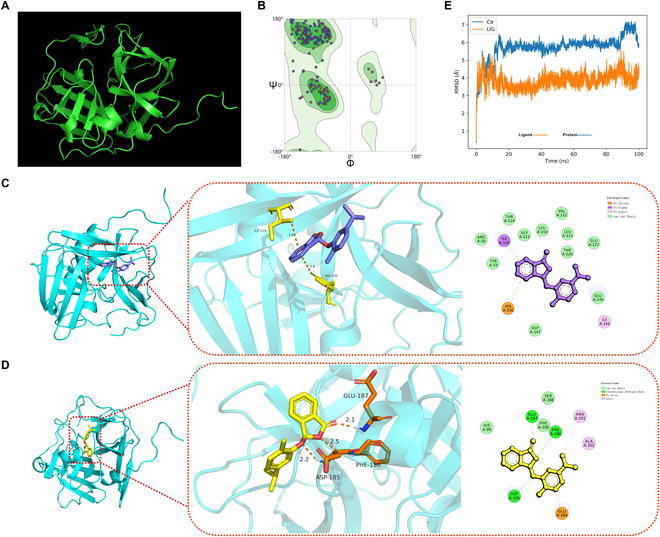
(A) The 3-dimensional (3D) structural model of PVY Nia protein was constructed by homology modeling. (B) Homology modeling to obtain the 3D structure model of the PVY Nia protein Ramachandran plot. (C) Molecular docking of (*R*)-3w with the 3D structural model of PVY Nia protein. (D) Molecular docking of (*S*)-3w with the 3D structural model of PVY Nia protein. (E) The binding stability of compound (*R*)-3w to the receptor protein was evaluated by surveying the root mean square deviation values of the initial positions of the atoms via molecular dynamics simulation.

The identification of crystal structures of molecular targets in pest organisms contributes to the opportunity for specific design of new pesticides [88]. So far, among the protein components of PVY, only the structural basis of CP and VPg has been elucidated [90]. To determine the binding sites of small molecules on Nia, we performed homology modeling with the reported sequence to construct a 3-dimensional (3D) structural model of PVY-Nia (Fig. 5A). In the Ramachandran plot analysis, it is evident that 94.55% (Fig. 5B and supporting data are presented in Fig. S1) of the amino acid residues are located within the favored regions, indicating that the constructed model is of satisfactory quality and can be utilized for further structural and functional studies. The molecular docking results (Fig. 5C and D) demonstrate that (*R*)-**3w** formed multiple interactions with PVY-Nia, including π–cation and π–alkyl interactions with H150, along with salt bridges. Additionally, I124 engaged in π–σ interactions with the compound. Quantitative analysis revealed bond lengths of 3.6 Å for the H150 π–cation interaction and 3.9 Å for the I124 π–σ interaction. Given that shorter bond lengths correlate with lower energy states (indicating enhanced system stability) and considering interaction multiplicity preferences [91–93], we prioritized H150 for mutagenesis. The calculated binding energy between (*R*)-**3w** and wild-type Nia protein was −5.884 kcal/mol. Subsequent mutations at I124 and H150 residues yielded binding energies of −5.853 and −5.158 kcal/mol, respectively. The substantial energy shift following H150 mutation indicates its critical role in maintaining binding stability. Notably, the less active (*S*)-**3w** enantiomer docking revealed no H150 interactions, confirming residue specificity. To thoroughly assess the binding stability of the compound (*R*)-**3w** with the receptor protein, we conducted molecular dynamics simulations. The conformational stability, represented by the changes in root-mean-square deviation over the simulation time, is illustrated in Fig. 5E. Through this simulation approach, we were able to observe and quantify in detail the stability of the binding between the compound (*R*)-**3w** and the receptor protein. Moreover, after (*R*)-**3w** binds to protein, the value becomes smaller. The overall stability is more stable. The analysis revealed that the (*R*)-**3w** PVY Nia complex attained stability after 15 ns, indicating that the system had reached an equilibrium state (Fig. 5E). Furthermore, the binding free energy of the compound (*R*)-**3w** to the PVY Nia molecular dynamics trajectory was calculated using the molecular mechanics Poisson–Boltzmann surface area method, yielding a value of −42.37 kcal mol^−1^. These preliminary experimental findings corroborate our hypothesis that the compound (*R*)-**3w** binds to Nia through π–cation interactions, π–alkyl interactions, and salt bridges and have identified a potential binding site (H150). These findings are consistent with the results of previous activity assays and support the hypothesis that the chirality-driven activity differences between the enantiomers are possibly attributed to their binding affinities to the potential target. In addition, the superior interaction strength and quantity of H150 in binding to the drug allow it to be speculated as a key site for the following investigation.

**Fig. 6.**
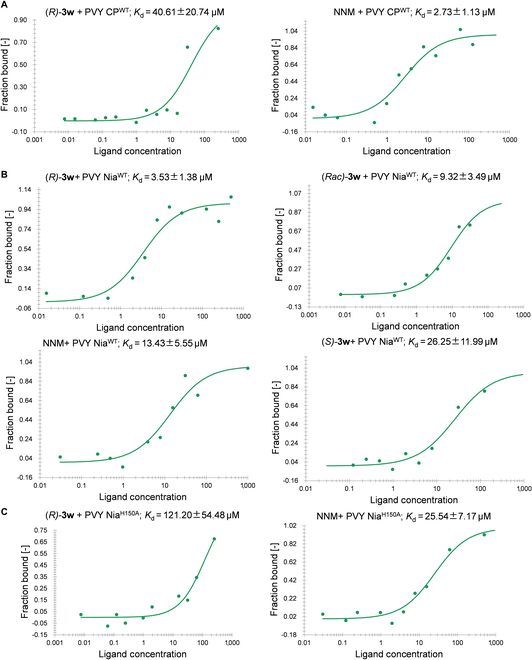
(A) MST results of (*R*)-3w, NNM, and PVY CP. (B) MST results of (*R*)-3w, (*Rac*)-3w, (*S*)-3w, and NNM PVY Nia. (C) MST results of (*R*)-3w, NNM, and PVY Nia mutants.

With the H150 site in hand, our next objective was to verify its accuracy in binding to our compounds. We constructed prokaryotic expression vectors for both wild-type PVY Nia (PVY Nia^WT^) and mutant PVY Nia (PVY Nia^H150A^), and expressed and purified the corresponding proteins using *Escherichia coli.* Microscale thermophoresis (MST), a widely employed technique for studying the interaction strength between small molecules and proteins, was used to investigate the affinity of (*R*)-**3w** to Nia. The results showed that compound (*R*)-**3w** had a weak interaction with PVY CP^WT^, with a dissociation constant (*K*_d_) of 40.61 μM, while NNM exhibited a much stronger interaction, with a *K*_d_ of 2.73 μM (Fig. 6A), indicating that (*R*)-**3w** does not share the same molecular target as NNM. Furthermore, compound (*R*)-**3w** demonstrated a strong binding affinity to PVY Nia^WT^, with a *K*_d_ of 3.53 μM. (*Rac*)-**3w** showed moderate binding affinities to both NNM and PVY Nia^WT^, with *K*_d_ values of 9.32 and 13.43 μM, respectively. Conversely, (*S*)-**3w** exhibited significantly weaker binding to PVY Nia^WT^, with a *K*_d_ of 26.25 μM (Fig. 6B). After mutating histidine at position 150 to alanine, the *K*_d_ value of (*R*)-**3w** binding to the mutant PVY Nia^H150A^ protein increased to 121.20 μM (Fig. 6C), indicating that the binding affinity of (*R*)-**3w** with PVY Nia^WT^ was significantly stronger than with PVY Nia^H150A^. These results are consistent with the previous molecular docking studies, further providing evidence for the proposal that (*R*)-**3w** targets Nia rather than CP, and that the binding ability of (*R*)-**3w** to Nia is configuration-dependent.

**Fig. 7.**
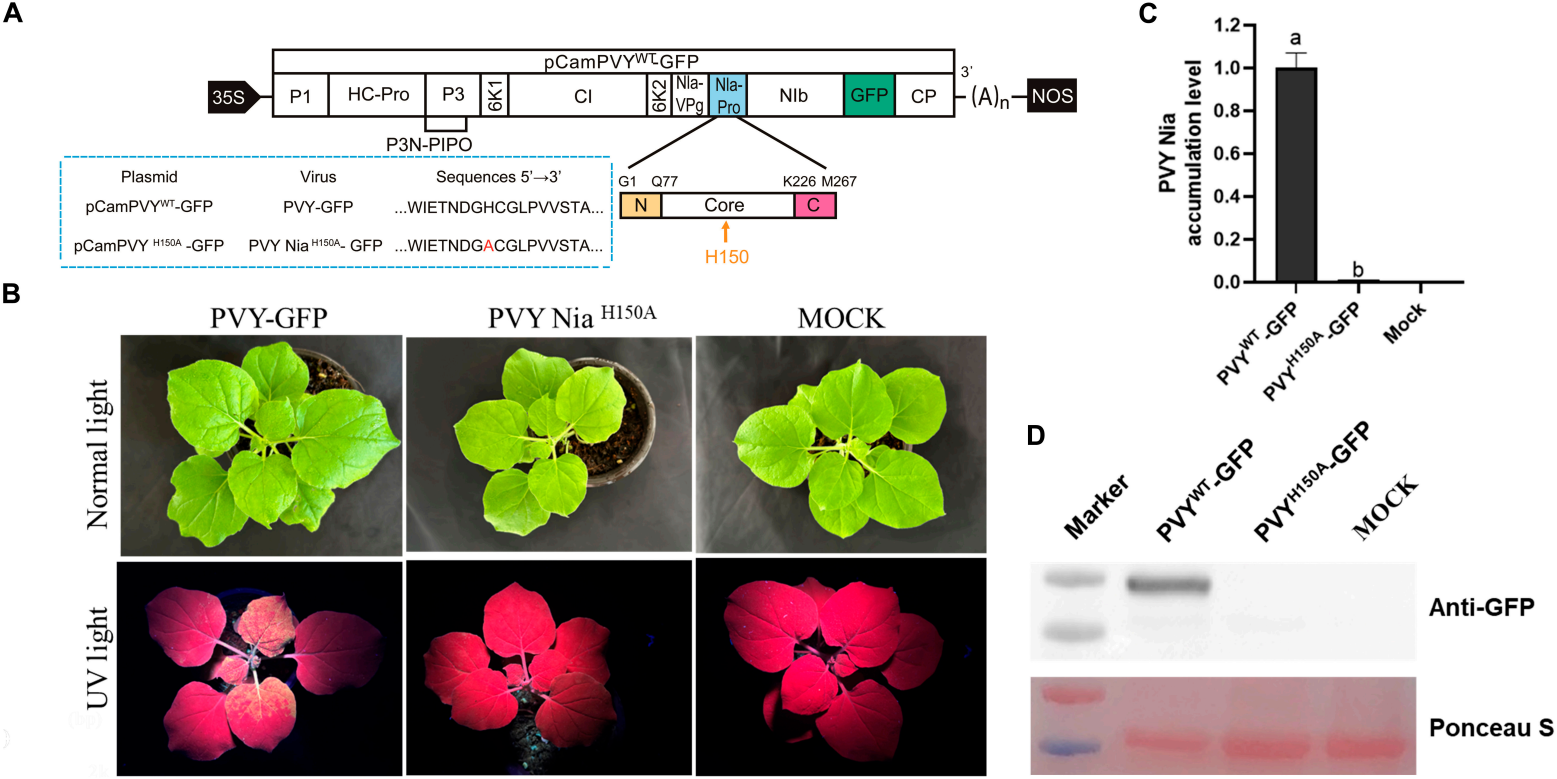
Residue 150, where compound (*R*)-3w binds to PVY Nia, is critical for PVY systemic infection and self-reproduction. (A) Schematic diagram of the pCamPVY-GFP genomic structure. H150 indicated by the yellow arrow are located in the core region of PVY Nia. The site-directed mutagenized and wild-type plasmids, viruses, and sequences are displayed in the blue-lined box. (B) Symptoms (top) and green fluorescence (bottom) under UV light of *N. benthamiana* infiltrated by wild-type and mutated PVY. (C and D) The accumulation levels of PVY Nia in the systemically infected leaves of the wild-type and mutated PVY-infected *N. benthamiana* plants were analyzed at 7 days postagroinfiltration (dpai) by Western blot and quantitative RT-PCR (RT-qPCR), respectively. Staining of RuBisCO with Ponceau S was used as a sample loading control. The data are shown as means ± SD from 3 biological replicates per treatment. Different letters mean statistically significant differences (*P* < 0.05, one-way analysis of variance).

While in vitro experiments have established that H150 is a critical site for (*R*)-**3w** interaction with Nia, whether this site mediates the proliferation of PVY remains unknown. Plant viruses rely on the cellular environment of their host for effective replication. Therefore, it is necessary to further investigate the impact of H150 on PVY pathogenicity in vivo. To rigorously validate in vivo whether histidine residue H150 in the Nia protein of PVY serves as a potential binding site for compound (*R*)-**3w** and its mediated functions, this study employed a site-directed mutagenesis strategy. Specifically, we substituted the H150 residue in the Nia protein of the pCamPVY-GFP plasmid with an alanine (Ala, A) residue [94], successfully constructing the mutant plasmid pCamPVY Nia^H150A^-GFP (Fig. 7A). *Agrobacterium* cultures harboring the plasmids pCamPVY-GFP and pCamPVY Nia^H150A^-GFP were infiltrated into *N. benthamiana* plants, respectively. Systemically infected PVY-GFP *N. benthamiana* plants exhibited typical curling and mosaic symptoms on their leaves. In contrast, plants inoculated with the mutant PVYNia^H150A^-GFP showed almost no apparent leaf symptoms 7 days postagroinfiltration (dpi) (as shown in the upper panel of Fig. 7B). Additionally, under ultraviolet (UV) light illumination, strong green fluorescent signals were observed in the young leaves of PVY-GFP-infected plants (as shown in the lower panel of Fig. 7B). The results from Western blot analysis, reverse transcription-polymerase chain reaction (RT-PCR), and quantitative RT-PCR (RT-qPCR) collectively demonstrate that PVY-GFP accumulates significantly in systemically infected leaves of *N. benthamiana*, whereas no accumulation of PVY is observed in the systemically infected leaves of *N. benthamiana* inoculated with PVY ^H150A^-GFP (as shown in Fig. 7C and D). Based on these findings, we conclude that the H150 residue in the Nia protein is crucial for the systemic infection and proliferation of PVY in *N. benthamiana* plants.

**Fig. 8.**
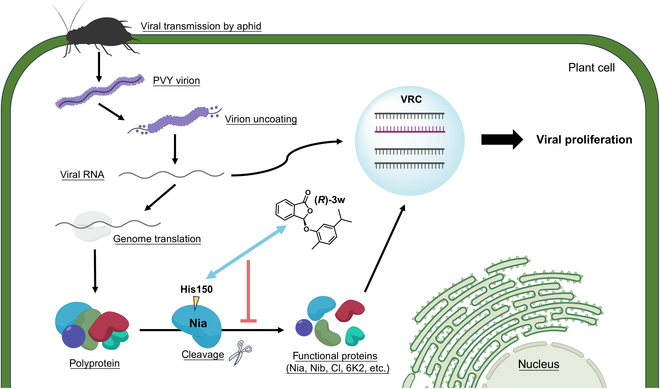
Proposed mechanism of antiviral action of (*R*)-3w by combining molecular docking and a series of molecular biological verifications. (*R*)-3w engages in an interaction with Nia via the H150 residue. Nia holds responsibility for truncating PVY polyprotein into smaller, functional proteins. The interaction perturbs the standard cleavage mechanism, consequently leading to a marked reduction in the amount of functional proteins essential for the assembly of the VRC.

PVY-Nia functions as a protease and plays a critical role in the upper stages of the viral life cycle [95]. It is involved in the cleavage of multiple polyprotein sites, and the resulting functional proteins mediate downstream pathogenic behaviors such as replication, assembly, and intercellular movement [96,97]. Additionally, reports have shown that Nia interacts with indole-3-acetic acid–amido synthetase within the host to regulate auxin homeostasis, thereby promoting disease development [98]. Transgenic potatoes expressing scFv antibody to PVY Nia exhibit a more substantial resistance to PVY compared to those expressing scFv-antibody against PVY CP [99]. Despite its potential as an ideal target, there are fewer cases of using small molecules to abnormalize Nia function for the treatment of plant diseases. Based on the results obtained, we reasonably propose that (*R*)-**3w** binds to Nia through the H150 site, thereby affecting its ability to cleave polyproteins. This leads to a reduction in the amount or functionality of downstream proteins that mediate the formation of the VRC, ultimately disrupting the normal proliferation of the virus within the host and achieving antiviral effects (Fig. 8).

## Conclusion

To summarize, we developed an NHC-mediated functionalization reaction that promoted the synthesis of a series of phthalide ether derivatives with good conversion rates and stereoselectivity. Our catalytic protocol showed excellent functional group tolerance and scalability. Screening for bioactivity against PVY led to the discovery of a potential chiral antiviral molecule, (*R*)-**3w**. Mechanistic studies both in vitro and in vivo collectively suggested that (*R*)-**3w** impacts the capacity of Nia to cleave polyproteins by binding to the H150 residue, leading to a reduction in quantity or functionality of cut proteins. These functional proteins are vital for unlocking the subsequent formation of VRC, which enables viral proliferation. Overall, this study contributes novel candidate molecule, synthetic method, and brand-new target protein for PVY management.

## Materials and Methods

### Chemicals and instruments

In the absence of specific notation, all reagents and solvents utilized throughout the experimental procedures were purchased from commercial sources. The chemical substances employed in this experiment, including *o*-phthalaldehyde (purity 98%; CAS: 643-79-8) and carvacrol (purity 98%; CAS: 499-75-2), were acquired from authorized commercial entities and were used without further purification. Phusion enzyme, DpnI enzyme, and DNase I enzyme were obtained from Thermo Fisher Scientific. Reverse transcriptase, Taq polymerase, and fluorescence quantitative reagent kits were purchased from Nanjing Novogene Biotechnology Co., Ltd. Trizol was obtained from Beijing Quanshijin Biotechnology Co., Ltd. Plasmid extraction kits were sourced from Omega Bio-Tek. His, glutathione S-transferase , and GFP antibodies were purchased from Wuhan Sanjing Biotechnology Co., Ltd. Enterokinase, protein markers, and DNA ladders were obtained from Shenggong Bioengineering (Shanghai) Co., Ltd.

### General synthetic method of target compounds 3a to 3z

To a solution of phthalaldehyde (0.12 mmol), substituted phenol (0.1 mmol), oxidant (0.12 mmol), base LiOH (0.2 mmol), and *N*-heterocarbene catalyst (0.02 mmol) in dichloromethane (2 ml), stirring was carried out overnight at room temperature. Upon completion of the reaction, the resultant mixture was initially subjected to vacuum distillation to remove the solvent, followed by purification via column chromatography. The obtained target compounds **3a** to **3z** were structurally characterized using proton nuclear magnetic resonance (^1^H NMR), ^13^C NMR, and high-resolution mass spectrometry . Furthermore, the absolute configuration of compound **3b** was unequivocally determined as the *R* configuration through x-ray crystallographic analysis, with the crystallographic data deposited at the Cambridge Crystallographic Data Centre (CCDC) under reference number 2395277. All detailed spectroscopic data and crystallographic information are included in the Supplementary Materials.

### Antiviral activity assay against PVY

The PVY virus was inoculated into tobacco cultivar k326 and purified according to previously reported methods [100–102]. The antiviral activity of the compounds against PVY was evaluated using the classical half-leaf lesion method on appropriate host plants of the genus Veronica [103]. Compounds were tested at a concentration of 500 μg/ml to assess their protective, curative, and inhibitory effects on PVY. Solutions of the compounds were prepared at concentrations of 500, 250, 125, 62.5, and 31.25 μg/ml to evaluate the EC_50_ values of the selected compounds. Ningnanmycin and ribavirin were used as positive controls. Three plant samples were treated for each compound. Five to seven days later, the number of lesions on both sides of the leaves was counted and recorded. The inhibition rate was calculated using the formula:

Inhibition rate (%) = (Number of necrotic spots on the left side of the leaf - Number of necrotic spots on the right side of the leaf) / Number of necrotic spots on the left side of the leaf × 100%

### Construction of an infectious wild-type clone

The wild-type plasmid pCamPVY-GFP is provided by Professors Xiangdong Li and Yanping Tian from Shandong Agricultural University [35,94]. This plasmid harbors a gene encoding GFP. Upon infection of plants such as *N. benthamiana*, it enables the expression of GFP, facilitating the visualization of gene expression through the emission of green fluorescence.

### Molecular docking

Initially, homology modeling of the PVY Nia protein was performed using SWISS-MODEL. This approach aids in predicting the 3D structure of the PVY Nia protein based on sequence similarity to known protein structures. Subsequently, the PVY Nia structure was refined using PyMOL software version 2.3.0 (Schrödinger, New York). Following this, the obtained 3D structure of the PVY Nia protein was subjected to molecular docking with the ligand (*R*)-**3w**. Flexible docking simulations were conducted using AutoDock 1.5.6 to elucidate the binding mode of (*R*)-**3w** with PVY Nia. Finally, the docking results were processed using PyMOL.

### Molecular dynamics trajectory simulations

Using the results of molecular docking as the initial conformation of the crystal, AmberTools23 was used to calculate the BCC charge of the ligand. AmberTools23 was used to build the ligand–protein complex simulation system. A box was built around the complex, with a minimum distance of 1.0 nm between the edge of the box and the protein–ligand complex. TIP3P water was added to the box, along with 3 Na^+^ to neutralize the system’s charge. The Amber 14SB force field was applied to the protein, solvent, and balancing ions, while the GAFF force field was applied to the ligand. Parmed was used to generate compatible run files for Gromacs 2023. Gromacs 2023 was used for system simulation. First, the system was energy-minimized using the steepest descent method, reaching the lowest energy in 1,205 steps. Then, 100 ps of constant-temperature equilibration and constant-pressure equilibration was performed separately. Finally, a 100-ns molecular dynamics simulation was conducted using the equilibrated results. The Gromacs trajconv module was used for preliminary processing of the simulation trajectory, including handling periodic boundary conditions and aligning the protein alpha carbon atoms to eliminate the translation and rotation of the protein–ligand complex system during the simulation. The processed trajectory was analyzed using MD analysis.

### Construction of prokaryotic expression plasmids for wild-type and mutant PVY Nia proteins

As described in Ref. [89], based on the wild-type protein gene, site-directed mutagenesis was performed to mutate the 150th amino acid residue from histidine (H150) to alanine (H150A), resulting in the PVY Nia^H150A^ plasmid for the subsequent expression of the mutant protein(PVY Nia^H150A^).

### Prokaryotic expression of PVY Nia and PVY Nia^H150A^ variants

Following the methodology outlined in Ref. [104], we have carried out the prokaryotic expression of PVY Nia and its mutant variant, PVY Nia^H150A^.

### MST determination of binding force

Initially, PVY Nia^WT^ and PVY Nia^H150A^ were labeled with fluorophores. Subsequently, the target compound and NNM were diluted to form solutions with concentration gradients. These solutions were mixed with an equal volume of protein and aspirated into capillaries for analysis. The binding fractions were plotted against the ligand concentrations, with the binding fractions serving as the ordinate and the ligand concentrations as the abscissa. By utilizing the obtained fitting data, the *K*_d_ value was derived [105,106].

### Construction of infectious mutant clone

As described in Ref. [35], site-directed mutagenesis was employed to mutate the H150 amino acid residue to A150 in the pCamPVY-GFP plasmid, based on the pCamPVY Nia^WT^ plasmid. This process facilitated the construction of the pCamPVY Nia^H150A^-GFP plasmid, which was subsequently utilized for the development of subsequent Agrobacterium plasmids.

### Construction of the mutant PVY Nia^H150A^-GFP agrobacterium plasmid

The plasmid was built using the pCam vector as the backbone. Agrobacterium-mediated transformation was then employed for the introduction of the mutant PVY viral infection strains. Site-directed mutagenesis PCR strategy was used to generate the mutation, and the potential key binding sites were verified through in vivo assays.

### RNA extraction, RT-PCR, and RT-qPCR

Initially, *N. benthamiana* leaves were finely powdered and subjected to total RNA extraction using TransZol reagent (TransGen Biotech, Beijing, China). Following the addition of chloroform, the mixture was allowed to stand. Centrifugation was performed to collect the supernatant, which was then mixed with isopropanol for precipitation, followed by another centrifugation step. The precipitate was treated with 75% ethanol, centrifuged once again, and resuspended in diethyl pyrocarbonate-treated water to obtain the RNA. To eliminate DNA contamination, gDNA wipeout enzyme (Vazyme, Nanjing, China) was utilized. Reverse transcription was carried out using gene-specific primers or random primers along with a reverse transcriptase kit (Vazyme, Nanjing, China). PCR amplification was performed using taq DNA polymerase (Vazyme, Nanjing, China), while RT-qPCR was conducted using SYBR Green qPCR Mix (Vazyme, Nanjing, China). Detailed information regarding the primers used in this study is described in Table S2.

### Western blotting analysis

Total protein extraction from *N. benthamiana* leaves was conducted as previously described. Protein separation and identification were conducted using a 12% sodium dodecyl sulfate–polyacrylamide gel electrophoresis method. Anti-GFP antibody (Proteintech, Wuhan) was used as the primary antibody, and horseradish peroxidase-labeled goat anti-rabbit IgG (Proteintech, Wuhan) served as the secondary antibody. The target protein signals were visualized using the ChemiDoc MP Imaging System (Bio-Rad).

## Data Availability

All data are available in the manuscript or the Supplementary Materials.
