# Supplementary material for: Carbene-Catalyzed Phthalide Ether Functionalization for Discovering Chiral Phytovirucide that Specifically Targets Viral Nia Protein to Inhibit Proliferation
Source: Research (Wash D C). 2025 Mar 14;8:0637. doi: 10.34133/research.0637 (PMC11908822; doi:10.34133/research.0637)
Supplement: Supplementary 1 — Supplementary Text Fig. S1 [file research.0637.f1.docx]

**Supporting Information**

**Carbene-catalyzed Phthalide Ether Functionalization for Discovering Chiral Phytovirucide that Specifically Targets Viral Nia Protein to Inhibit Proliferation**

Xiaoyi Wang^a^, Weijia Yang^a^, Shang Wu^a^, Fangru Jin^a^, Zhongjie Shen^a^, Xiangyang Li^a,^*, Yonggui Robin Chi^a,b^, Baoan Song^a^, Runjiang Song^a,^*

^a^ *State Key Laboratory of Green Pesticide, Center for R&D of Fine Chemicals of Guizhou University, Guiyang 550025, P. R. China.*

^b^ *School of Chemistry, Chemical Engineering, and Biotechnology, Nanyang Technological University, Singapore 637371, Singapore*.

* Corresponding authors.

*Contents*

[I. General information 1](#_Toc185802311)

[II. Preparation of substrates 3](#_Toc185802312)

[III. Reaction condition optimization 4](#_Toc185802313)

[IV. General procedure for the catalytic reactions 5](#_Toc185802314)

[VI. Antiviral Bioassay 6](#_Toc185802315)

[VII. Mechanism of Action Experimental Section 8](#_Toc185802316)

[VIII. Characterization of products 10](#_Toc185802317)

[IX. NMR spectra of products 23](#_Toc185802318)

[X. HPLC spectra of products 61](#_Toc185802319)

**I. General information**

Commercially available materials purchased from Aladdin or J&K were used as received. Unless otherwise specified, all reactions were carried out in 10 mL dry Schlenk tube. Proton nuclear magnetic resonance (^1^H NMR) spectra were recorded on a Bruker (400 MHz) spectrometer or on a JEOL-ECX-500 (500 MHz) spectrometer. Chemical shifts were recorded in parts per million (ppm, δ) relative to tetramethylsilane (δ 0.00) or the corresponding deuterium solvent. ^1^H NMR splitting patterns are designated as singlet (s), doublet (d), triplet (t), quartet (q), dd (doublet of doublets); m (multiplets), and etc. All first-order splitting patterns were assigned on the basis of the appearance of the multiplet. Splitting patterns that could not be easily interpreted are designated as multiplet (m) or broad (br). Carbon nuclear magnetic resonance (^13^C NMR) spectra were recorded on a Bruker (101 MHz) spectrometer or on a JEOL-ECX-500 (126 MHz) spectrometer. The melting points (m.p.) of the title compounds were determined when left untouched on an XT-4-MP apparatus from Beijing Tech. Instrument Co. (Beijing, China). High resolution mass spectral analysis (HRMS) was performed on a quadrupole/electrostatic field orbitrap mass spectrometer. Absolute configuration of the products was determined by X-ray crystallography. The determination of enantiomeric excess was performed via chiral HPLC analysis using Shimadzu LC-20AD HPLC workstation. Optical rotations were measured using a 1 mL cell with a 1 dm path length on a Jasco P-1030 polarimeter and are reported as follows: [α]^rt^_D_ (c in g per 100 mL solvent). Analytical thin-layer chromatography (TLC) was carried out on Merck 60 F254 pre-coated silica gel plate (0.2 mm thickness). Visualization was performed using a UV lamp.

**II. Preparation of substrates**

Combining a slightly modified literature method, chiral isobenzofuranone derivatives were synthesized using phenolic compounds and *o*-Phthalaldehyde as raw materials.

**Method 1**

To a solution of phthalaldehyde **2**(0.12 mmol), substituted phenol **1**(0.1 mmol), oxidant (0.12 mmol), base LiOH (0.2 mmol), and *N*-heterocarbene catalyst (0.02 mmol) in dichloromethane (2.0 ml.), stirring was carried out overnight at room temperature. Upon completion of the reaction, the mixture was extracted and purified by column chromatography to yield the *R*-configured compounds **3a-3z**.

**III. Reaction condition optimization**

**Table S1.** Screening of different catalysts, bases, solvents and temperatures^a^

| Entry | NHC | Base | Condition | Yield [%]^b^ | er^c^ |
| --- | --- | --- | --- | --- | --- |
| 1 | A | Et_3_N | CH_2_Cl_2_ | 80.46 | 75:25 |
| 2 | B | Et_3_N | CH_2_Cl_2_ | 82.23 | 79:21 |
| 3 | C | Et_3_N | CH_2_Cl_2_ | 67.01 | 75:25 |
| 4 | D | Et_3_N | CH_2_Cl_2_ | 87.85 | 69:31 |
| 5 | E | Et_3_N | CH_2_Cl_2_ | 83.55 | 92:8 |
| 6 | E | K_2_CO_3_ | CH_2_Cl_2_ | 83.11 | 94:6 |
| 7 | E | DBU | CH_2_Cl_2_ | 16.36 | 65:35 |
| 8 | E | LiOH·H_2_O | CH_2_Cl_2_ | 89.30 | 95:5 |
| 9 | E | DABCO | CH_2_Cl_2_ | 42.44 | 78:22 |
| 10^d^ | E | LiOH | CH_2_Cl_2_ | 87.98 | 98:2 |
| 10 | E | LiOH | PhMe | 56.15 | 96:4 |
| 11 | E | LiOH | Hexane | - | - |
| 12 | E | LiOH | THF | - | - |
| 13 | E | LiOH | EA | - | - |
| 14^e^ | E | LiOH | CH_2_Cl_2_ | 84.00 | 97:3 |
| 15^f^ | E | LiOH | CH_2_Cl_2_ | 83.55 | 99:1 |
| 16^g^ | E | LiOH | CH_2_Cl_2_ | 86.65 | 94:6 |
| 17^h^ | E | LiOH | CH_2_Cl_2_ | 81.34 | 94:6 |
| 18^i^ | E | LiOH | CH_2_Cl_2_ | 88.42 | 94:6 |
| 19^j^ | E | LiOH | CH_2_Cl_2_ | 91.07 | 98:2 |

^a^General reaction conditions: **1a** (0.1 mmol), **2a** (0.12 mmol), NHC (20 mol%), base (200 mol%), DQ (120 mol%), CH_2_Cl_2_ (2 mL), 12 h、r.t.; ^b^Isolated yield; ^c^Determined by chiral HPLC analysis (IB column, 0.6 mL/min, hexane/iPrOH = 80/20); ^d^**1a** (0.1 mmol), **2a** (0.12 mmol), NHC (20 mol%), base (200 mol%), CH_2_Cl_2_ (2 mL), 12 h、r.t; ^e^**1a** (0.1mmol), **2a** (0.12 mmol), NHC (20 mol%), DQ (120 mol%), base (200 mol%), solvent (2 mL), 12 h, 0 ̊C; ^f^**1a** (0.1mmol), **2a** (0.12 mmol), NHC (20 mol%), DQ (120 mol%) base (200 mol%), solvent (2 mL), 12 h, 10 ̊C; ^g^used 150 mol% base LiOH, other conditions were the same as **d**. ^h^used 5 mol% NHC, other conditions were the same as **d**. ^i^Used 10 mol% NHC, other conditions were the same as **d**. ^j^**1a** (0.1 mmol), **2a** (0.12 mmol), NHC (20 mol%), DQ (120 mol%) base (200 mol%), 4Å MS (80 mg), solvent (2 mL), 12 h, r.t.. THF = tetrahydrofuran. DBU = 1,8-diazabicyclo[5.4.0]undec-7-ene. DABCO = 1,4-diazabicyclo[2.2.2]octane. Et_3_N = triethylamine. CH_2_Cl_2_ = dichloromethane. r.t. = room temperature.

**IV. General procedure for the catalytic reactions**

Procedure for synthesis of chiral product **3w** with gram-scale:

To a 10 mL round bottom flask tube with a stirring bar, carvacrol (10 mmol, 1.50 g) **1**, *o*-phthalaldehyde **2** (12 mmol, 1.61 g), DQ (12 mmol, 4.90 g), base LiOH (20 mmol, 0.48 g), *N*-heterocarbene catalyst (2 mmol, 0.48 g) and 4Å molecular sieves (8.0 g) were added. Anhydrous CH_2_Cl_2_ (20 mL) was added. The mixture was monitored by TLC plate until the reaction was completed at room temperature overnight (12 h). The mixture was concentrated and purified by silica gel column chromatography using CH_2_Cl_2_/petroleum ether (1:1) as eluent to obtain the product **3w** (1.92 g, 68%, 97:3 er).

**V. X-ray crystallography of product 3b.**

The colorless crystal of product **3b** was obtained by vaporization of a CH_2_Cl_2_ / methanol solution, and its absolute configuration was determined via X-ray structure analysis. CCDC 2395277 contains the supplementary crystallographic data that can be obtained free of charge from The Cambridge Crystallographic Data Centre via www.ccdc.cam.ac.uk/ data_request/cif.

**VI. Antiviral Bioassay**

Extraction of potato virus Y (PVY). Viruses were propagated in *Nicotiana. tabacum* cv. K326, ground in phosphate buffer and filtered with a double-layer pledget. The extract was centrifuged for 5 min at 10000×g, and the supernatant was used as the crude extract of the virus. The extraction process was carried out at 4 ̊C.

**Curative activities of target compounds against PVY *in vivo*.** Chenopodium *amaranticolor* plants were used to evaluate the anti-PVY activities. The crude extracts of PVY were dipped and inoculated on the whole leaves, which were scattered with silicon carbide beforehand. The leaves were washed with water after inoculation for 30 minutes and then dried. The target compound solution was smeared on the right side of *Chenopodium* *amaranticolor* leaves, and solvent was smeared on the left side, which served as the control. All the plants were cultivated in an incubator under an illumination of 10 000 lx at 28 ± 2 ̊C. The number of local lesions appearing 5 to 6 days after inoculation was counted. Measurements were performed in triplicate.

**Protective activities of target compounds against PVY *in vivo*.** The target compound solution was smeared on the right side of Chenopodium amaranticolor leaves, and solvent was smeared on the left side, which served as the control. Chenopodium amaranticolor plants were inoculated with PVY after 24 hours, which were scattered with silicon carbide beforehand. The leaves were washed with water after inoculation for 30 minutes. All the plants were cultivated in an incubator under an illumination of 10 000 lx at 28 ± 2 ̊C. The number of local lesions appearing 5 to 6 days after inoculation was counted. Measurements were performed in triplicate. Measurements were performed in triplicate.

**Inactive activities of target compounds against PVY *in vivo*.** The virus was inhibited by mixing it with compound solution with the same volume for 30 min. The right side of *Chenopodium* *amaranticolor* leaves with silicon carbide was inoculated with the mixture, whereas the left side of each leaf, which served as the control, was inoculated with the mixture containing the solvent and the virus. All the plants were cultivated in an incubator under an illumination of 10 000 lx at 28 ± 2 ̊C. The number of local lesions appearing 5 to 6 days after inoculation was counted. Measurements were performed in triplicate.

**VII. Mechanism of Action Experimental Section**

**Homology modeling**

Initially, homology modeling of the PVY Nia protein was performed using SWISS-MODEL. This approach aids in predicting the three-dimensional (3D) structure of the PVY Nia protein based on sequence similarity to known protein structures. Additionally, the results of our homology modeling are presented in **Fig.** S1.


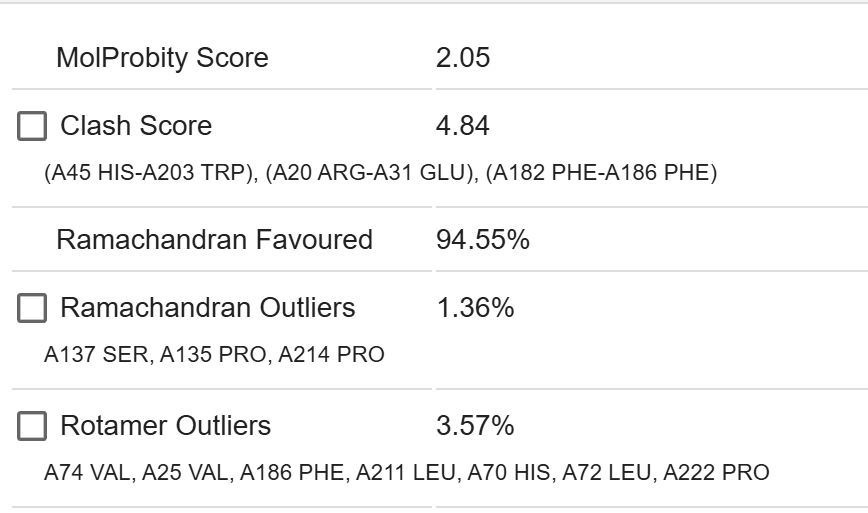


**Fig.** S1. The quality of the homology modeling was assessed using a Ramachandran plot.

**Plant growth, virus inoculation, and protein transient expression.** A greenhouse was used to cultivate *N. tabacum* cv. K326, *N. benthamiana*, and *Chenopodium Amaranticolor* plants maintained at a 6/18-hour (dark/light) photoperiod and 25 ̊C. Individual plasmids were transformed into the GV3101 strain of agrobacterium tumefaciens. Cultivation of agrobacterium cultures containing different plasmids, dilution of agrobacterium cultures, and transient expression analysis refer to previous reports.

**RNA extraction, RT-PCR, and RT-qPCR.** The leaves of the assayed *N. benthamiana* were used to extract total RNA by TransZol reagent (TransGen Biotech, Beijing, China), and DNA contamination was eliminated by a gDNA wipe enzyme (Vazyme, Nanjing, China). Reverse transcription was carried out using gene-specific primers or random primers and a reverse transcriptase kit (Vazyme, Nanjing, China) according to the instructions. The taq DNA polymerase (Vazyme, Nanjing, China) was used to execute PCR. RT-qPCR was conducted using the SYBR Green qPCR mix (Vazyme, Nanjing, China). The primers mentioned in this study are described in **Table S2**.

Primers used in this study

**Table S2.** Primers used in this study

| Primes | Sequences 5’→3’ |
| --- | --- |
| PVY-Nia^H150A^-F | GAAACAAATGATGGGGCGTGTGGATTACCAGTAGTGAGTA |
| PVY- Nia^H150A^ -R | CTCACTACTGGTAATCCACACGCCCCATCATTTGTTTCAA |
| PVY-GFP^H150A^-F | GGTATTGCTAAGTTTCCTG |
| PVY- GFP^H150A^ -R | TGTTTCGTGCACCAGTTT |
| qPCR-PVY Nia-F | GGAATACACAGCTTGGCGAAT |
| qPCR-PVY Nia-F | TGGTCCACTCATTATGCTCATT |
| qPCR- EF1𝛼 -F | CACACTGGAGTGATGGTTGG |
| qPCR- EF1𝛼 -R | GGTGTGGTGCCAAATCTTCT |

**VIII. Characterization of products**

**(*R*)-3-(4-fluorophenoxy)isobenzofuran-1(3*H*)-one**

White solid, m.p. 124.7-126.5 ̊C, 72.94% yield.

[α] ^28^_D_. = -264.3 (c = 0.330 in CHCl_3_)

^1^H NMR(500 MHz, CDCl_3_) δ 7.93 (dt, *J* = 7.7, 1.0 Hz, 1H), 7.77 (td, *J* = 7.5, 1.1 Hz, 1H), 7.71 – 7.63 (m, 2H), 7.21 – 7.14 (m, 2H), 7.07 – 6.99 (m, 2H), 6.76 (s, 1H).

^13^C NMR (101 MHz, CDCl_3_) δ 168.12 (s), 159.08 (d, *J* = 242.0 Hz), 152.58 (d, *J* = 2.5 Hz), 144.28 (s), 134.79 (s), 131.34 (s), 126.81 (s), 125.74 (s), 123.80 (s), 118.85 (d, *J* = 8.3 Hz), 116.30 (d, *J* = 23.3 Hz), 100.19 (s).

HRMS (ESI, m/z): calculated for C_14_H_9_FO_3_Na[M+Na]^+^: 267.0428, found: 267.0423.

HPLC analysis:97.8:2.2 e.r. (Chiralpak IB, Hexane/2-PrOH = 80/20, 0.6 mL/min), Rt (major) = 16.1 min, Rt (minor) = 14.0min.

**(*R*)-3-(4-chlorophenoxy)isobenzofuran-1(3*H*)-one**

White solid, m.p. 180.6-181.9 ̊C, 89.97% yield.

[α] ^28^_D_. = -259.9 (c = 0.330 in CHCl_3_)

^1^H NMR (500 MHz, CDCl_3_) δ 7.93 (dt, *J* = 7.7, 1.0 Hz, 1H), 7.78 (td, *J* = 7.5, 1.1 Hz, 1H), 7.71 – 7.63 (m, 2H), 7.34 – 7.28 (m, 2H), 7.19 – 7.11 (m, 2H), 6.79 (s, 1H).

^13^C NMR (101 MHz, CDCl_3_) δ 168.03, 155.13, 144.18, 134.84, 131.40, 129.76, 128.92, 126.72, 125.78, 123.81, 118.54, 99.56.

HRMS (ESI, m/z): calculated for C_14_H_9_ClO_3_Na [M+Na]^+^: 283.0132, found: 283.0131.

HPLC analysis:91.0:9.0 e.r. (Chiralpak IB, Hexane/2-PrOH = 80/20, 0.6 mL/min), Rt (major) = 10.4 min, Rt (minor) = 12.3min.

**(*R*)-3-(4-bromophenoxy)isobenzofuran-1(3*H*)-one**

White solid, m.p. 185.6-187.0 ̊C, 72.40% yield.

[α] ^28^_D_. = -261.5 (c = 0.330 in CHCl_3_)

^1^H NMR (500 MHz, CDCl_3_) δ 7.94 (d, *J* = 7.6 Hz, 1H), 7.78 (td, *J* = 7.6, 1.2 Hz, 1H), 7.71 – 7.65 (m, 2H), 7.48 – 7.44 (m, 2H), 7.14 – 7.07 (m, 2H), 6.79 (s, 1H).

^13^C NMR (101 MHz, CDCl_3_) δ 167.99, 155.67, 144.17, 134.83, 132.72, 131.40, 126.72, 125.78, 123.80, 118.97, 116.39, 99.45.

HRMS (ESI, m/z): calculated for C_14_H_9_BrO_3_Na [M+Na]^+^: 326.9627, found: 326.9618.

HPLC analysis:90.1:9.9 e.r. (Chiralpak IB, Hexane/2-PrOH = 80/20, 0.6 mL/min), Rt (major) =10.1 min, Rt (minor) =11.7min.

**(*R*)-3-(4-ethylphenoxy)isobenzofuran-1(3*H*)-one**

White solid, m.p. 76.8-78.1 ̊C, 81.93% yield.

[α] ^28^_D_. = -319.4 (c = 0.330 in CHCl_3_)

^1^H NMR (500 MHz, CDCl_3_) δ 7.93 (d, *J* = 7.6 Hz, 1H), 7.77 (t, *J* = 7.5 Hz, 1H), 7.72 – 7.62 (m, 2H), 7.18 (d, *J* = 8.7 Hz, 2H), 7.15 (d, *J* = 8.6 Hz, 2H), 6.81 (s, 1H), 2.63 (q, *J* = 7.6 Hz, 2H), 1.22 (t, J = 7.6 Hz, 3H).

^13^C NMR (101 MHz, CDCl_3_) δ 168.30, 154.74, 144.62, 139.78, 134.68, 131.18, 129.08, 126.90, 125.64, 123.79, 117.12, 100.09, 28.14, 15.84.

HRMS (ESI, m/z): calculated for C_16_H_15_O_3_ [M+H]^+^: 255.1016, found: 255.1009.

HPLC analysis:92.7:7.3 e.r. (Chiralpak IB, Hexane/2-PrOH = 80/20, 0.6 mL/min), Rt (major) =8.3 min, Rt (minor) =9.3min.

**(*R*)-3-(p-tolyloxy)isobenzofuran-1(3*H*)-one**

White solid, m.p. 133.0-137.6 ̊C, 93.25% yield.

[α] ^28^_D_. = -350.8 (c = 0.330 in CHCl_3_)

^1^H NMR (500 MHz, CDCl_3_) δ 7.93 (d, *J* = 7.6 Hz, 1H), 7.77 (td, *J* = 7.5, 1.2 Hz, 1H), 7.69 (d, *J* = 7.5 Hz, 1H), 7.67 – 7.63 (m, 1H), 7.15 (d, *J* = 8.7 Hz, 2H), 7.13 – 7.10 (m, 2H), 6.80 (s, 1H), 2.32 (s, 3H).

^13^C NMR (101 MHz, CDCl_3_) δ 154.57, 144.60, 134.71, 133.33, 131.20, 130.25, 126.89, 125.64, 123.82, 117.12, 100.13, 30.36, 20.69.

HRMS (ESI, m/z): calculated for C_15_H_12_O_3_Na [M+Na]^+^: 263.0679, found: 263.0674.

HPLC analysis:96.8:3.2 e.r. (Chiralpak IB, Hexane/2-PrOH = 80/20, 0.6 mL/min), Rt (major) =9.5 min, Rt (minor) =11.3min.

**(*R*)-3-(4-methyl-3-nitrophenoxy)isobenzofuran-1(3*H*)-one**

White solid, m.p. 133.3-135.5 ̊C, 68.07% yield.

[α] ^28^_D_. = -366.4 (c = 0.330 in CHCl_3_)

^1^H NMR (500 MHz, CDCl_3_) δ 7.95 (d, *J* = 7.6 Hz, 1H), 7.84 (d, *J* = 2.6 Hz, 1H), 7.80 (t, *J* = 7.5 Hz, 1H), 7.74 – 7.65 (m, 2H), 7.38 (dd, *J* = 8.5, 2.7 Hz, 1H), 7.33 (d, *J* = 8.5 Hz, 1H), 6.85 (s, 1H), 2.57 (s, 3H).

^13^C NMR (101 MHz, CDCl_3_) δ 168.05, 157.07, 144.77, 144.26, 134.86, 134.63, 132.69, 131.42, 128.71, 127.48, 126.75, 125.82, 123.82, 118.98, 117.62, 110.76, 99.24.

HRMS (ESI, m/z): calculated for C_15_H_11_NO_5_Na [M+Na]^+^: 308.0529, found: 308.0521.

HPLC analysis:95.1:4.9 e.r. (Chiralpak IB, Hexane/2-PrOH = 80/20, 0.6 mL/min), Rt (major) =16.8 min, Rt (minor) =21.0min.

**(*R*)-3-(3-chlorophenoxy)isobenzofuran-1(3*H*)-one**

White solid, m.p. 125.5-127.3 ̊C, 78.12% yield.

[α] ^28^_D_. = -329.1 (c = 0.330 in CHCl_3_)

^1^H NMR (500 MHz, CDCl_3_) δ 7.94 (d, *J* = 7.6 Hz, 1H), 7.82 – 7.76 (m, 1H), 7.72 – 7.64 (m, 2H), 7.29 (t, *J* = 8.2 Hz, 1H), 7.12 (dt, *J* = 7.8, 2.3 Hz, 2H), 6.81 (s, 1H).

^13^C NMR (101 MHz, CDCl_3_) δ 158.10, 154.67, 143.66, 139.49, 135.33, 134.80, 133.86, 131.73, 125.86, 124.26, 117.31, 99.65.

HRMS (ESI, m/z): calculated for C_14_H_9_ClO_3_Na [M+Na]^+^: 283.0132, found: 283.0126.

HPLC analysis:92.4:7.6 e.r. (Chiralpak IB, Hexane/2-PrOH = 80/20, 0.6 mL/min), Rt (major) =11.6 min, Rt (minor) =15.1min.

**(*R*)-3-(4-(trifluoromethyl)phenoxy)isobenzofuran-1(3*H*)-one**

Yellow solid, m.p. 89.7-90.8 ̊C, 96.72% yield.

[α] ^28^_D_. = -261.5 (c = 0.330 in CHCl_3_)

^1^H NMR (500 MHz, CDCl_3_) δ 7.96 (d, *J* = 7.6 Hz, 1H), 7.82 – 7.78 (m, 1H), 7.70 (dd, *J* = 14.1, 7.3 Hz, 2H), 7.66 – 7.62 (m, 2H), 7.31 (d, *J* = 8.6 Hz, 2H), 6.88 (s, 1H).

^﻿13^C NMR (151 MHz, Chloroform-d) δ 167.86, 158.98, 144.10, 134.99, 131.58, 127.34, 126.72, 125.94 (d, *J* = 33.3 Hz), 125.94, 124.14 (d, *J* = 271.5 Hz), 123.88, 117.03, 98.82.

HRMS (ESI, m/z): calculated for C_15_H_9_F_3_O_3_Na [M+Na]^+^: 317.0396, found: 317.0393.

HPLC analysis:91.8:8.2 e.r. (Chiralpak IB, Hexane/2-PrOH = 80/20, 0.6 mL/min), Rt (major) =19.5 min, Rt (minor) =20.5min.

**(*R*)-3-(2-(trifluoromethyl)phenoxy)isobenzofuran-1(3*H*)-one**

Yellow solid, m.p. 100.1-103.8 ̊C, 84.63% yield.

[α] ^28^_D_. = -249.8 (c = 0.330 in CHCl_3_)

^﻿﻿1^H NMR (500 MHz, CDCl_3_) δ 7.94 (d, *J* = 7.6 Hz, 1H), 7.79 (td, *J* = 7.5, 1.1 Hz, 1H), 7.73 (d, *J* = 7.6 Hz, 1H), 7.68 (d, *J* = 7.5 Hz, 1H), 7.66 – 7.63 (m, 2H), 7.63 – 7.58 (m, 2H), 6.74 (s, 1H).

﻿^13^C NMR (151 MHz, Chloroform-d) δ 168.00, 154.84, 144.24, 135.12, 133.89, 131.50, 127.20 (q, *J* = 4.9 Hz), 126.67, 125.73, 124.15, 123.86, 123.42 (d, *J* = 271.9 Hz), 120.78, 118.50, 100.56.

HRMS (ESI, m/z): calculated for C_15_H_9_F_3_O_3_Na [M+Na]^+^: 317.0396, found: 317.0391.

HPLC analysis:81.0:19.0 e.r. (Chiralpak IB, Hexane/2-PrOH = 80/20, 0.6 mL/min), Rt (major) =8.9 min, Rt (minor) =10.4min.

**(*R*)-3-(4-(trifluoromethoxy)phenoxy)isobenzofuran-1(3*H*)-one**

White solid, m.p. 89.9-91.6 ̊C, 59.18% yield.

[α] ^28^_D_. = -248.8 (c = 0.330 in CHCl_3_)

^1^H NMR (500 MHz, CDCl_3_) δ 7.95 (d, *J* = 7.6 Hz, 1H), 7.79 (td, *J* = 7.5, 1.2 Hz, 1H), 7.68 (h, *J* = 7.4 Hz, 3H), 7.23 (d, *J* = 2.7 Hz, 3H), 6.81 (s, 1H).

^13^C NMR (101 MHz, CDCl_3)_ δ 167.97 (s), 154.96 (s), 144.97 (s), 144.13 (s), 134.87 (s), 131.44 (s), 126.70 (s), 125.81 (s), 123.80 (s), 122.70 (s), 120.48 (d, *J* = 256.8 Hz), 118.21 (s), 99.51 (s).

HRMS (ESI, m/z): calculated for C_15_H_9_F_3_O_4_Na [M+Na]^+^: 333.0345, found: 333.0338.

HPLC analysis:92.6:7.4 e.r. (Chiralpak IB, Hexane/2-PrOH = 80/20, 0.6 mL/min), Rt (major) =9.2 min, Rt (minor) =8.2min.

**(*R*)-3-(3,5-di-tert-butylphenoxy)isobenzofuran-1(3*H*)-one**

White solid, m.p. 93.8-95.7 ̊C, 65.16% yield.

[α] ^28^_D_. = -252.8 (c = 0.330 in CHCl_3_)

﻿1H NMR (500 MHz, CDCl_3_) δ 7.93 (d, *J* = 7.6 Hz, 1H), 7.77 (t, *J* = 7.4 Hz, 1H), 7.70 (d, *J* = 7.8 Hz, 1H), 7.65 (t, *J* = 7.5 Hz, 1H), 7.19 (t, *J* = 1.7 Hz, 1H), 7.06 (d, *J* = 1.7 Hz, 2H), 6.89 (s, 1H), 1.33 (s, 18H).

^13^C NMR (101 MHz, CDCl_3_) δ 168.44, 156.42, 152.85, 144.78, 134.65, 131.13, 126.94, 125.61, 123.79, 117.84, 111.29, 99.94, 35.11, 31.43.

HRMS (ESI, m/z): calculated for C_22_H_26_O_3_Na [M+Na]^+^: 361.1774, found: 361.1765.

HPLC analysis:96.5:3.5 e.r. (Chiralpak IB, Hexane/2-PrOH = 80/20, 0.6 mL/min), Rt (major) =6.4 min, Rt (minor) =6.8min.

**(*R*)-3-(4-acetylphenoxy)isobenzofuran-1(3*H*)-one**

White solid, m.p. 162.9-164.9 ̊C, 82.76% yield.

[α] ^28^_D_. = -432.3 (c = 0.330 in CHCl_3_)

^1^H NMR (500 MHz, CDCl_3_) δ 8.00 (d, *J* = 2.1 Hz, 1H), 7.99 (d, *J* = 2.0 Hz, 1H), 7.96 (dd, *J* = 7.7, 1.1 Hz, 1H), 7.80 (td, *J* = 7.5, 1.1 Hz, 1H), 7.73 – 7.67 (m, 3H), 7.26 – 7.24 (m, 2H), 6.92 (s, 1H), 2.59 (s, 3H)

^13^C NMR (101 MHz, CDCl_3_) δ 168.05, 157.07, 144.77, 144.26, 134.86, 134.63, 132.69, 131.42, 128.71, 127.48, 126.75, 125.82, 123.82, 118.98, 117.62, 110.76, 99.24.

HRMS (ESI, m/z): calculated for C_16_H_12_O_4_Na [M+Na]^+^: 291.0628, found: 291.0623.

HPLC analysis:93.6:6.4 e.r. (Chiralpak IB, Hexane/2-PrOH = 80/20, 0.6 mL/min), Rt (major) =20.2 min, Rt (minor) =21.9min.

**(*R*)-3-(4-methoxyphenoxy)isobenzofuran-1(3*H*)-one**

White solid, m.p. 162.9-164.9 ̊C, 82.76% yield.

[α] ^28^_D_. = -217.7 (c = 0.330 in CHCl_3_)

^1^H NMR (500 MHz, CDCl_3_) δ 7.92 (d, *J* = 7.6 Hz, 1H), 7.77 (td, *J* = 7.5, 1.1 Hz, 1H), 7.70 (d, *J* = 7.6 Hz, 1H), 7.65 (t, *J* = 7.4 Hz, 1H), 7.20 – 7.13 (m, 2H), 6.90 – 6.84 (m, 2H), 6.75 (s, 1H), 3.79 (s, 3H)

^13^C NMR (101 MHz, CDCl_3_) δ 168.32, 156.06, 150.48, 144.55, 134.66, 131.18, 126.95, 125.63, 123.80, 118.85, 114.74, 100.84, 55.70.

HRMS (ESI, m/z): calculated for C_15_H_12_O_4_Na [M+Na]^+^: 279.0628, found: 279.0620.

HPLC analysis:91.0:9.0 e.r. (Chiralpak IB, Hexane/2-PrOH = 80/20, 0.6 mL/min), Rt (major) =19.9 min, Rt (minor) =21.8min.

**(*R*)-3-(4-acetyl-2-methylphenoxy)isobenzofuran-1(3*H*)-one**

White solid, m.p. 133.9-135.8 ̊C, 79.15% yield.

[α] ^28^_D_. = -341.1 (c = 0.330 in CHCl_3_)

^1^H NMR (500 MHz, CDCl_3_) δ 7.99 – 7.94 (m, 1H), 7.86 (dd, *J* = 8.6, 2.3 Hz, 1H), 7.83 (dd, *J* = 5.4, 1.6 Hz, 1H), 7.81 – 7.79 (m, 1H), 7.69 (t, *J* = 7.4 Hz, 2H), 7.40 (d, *J* = 8.6 Hz, 1H), 6.88 (s, 1H), 2.58 (s, 3H), 2.26 (s, 3H).

^13^C NMR (101 MHz, CDCl_3_) δ 197.20, 167.93, 158.68, 144.32, 134.94, 131.45, 131.40, 130.64, 130.16, 128.42, 127.93, 125.88, 123.70, 114.23, 99.02, 26.56, 16.35.

HRMS (ESI, m/z): calculated for C_17_H_14_O_4_Na [M+Na]^+^: 305.0784, found: 305.0779.

HPLC analysis:93.6:6.4 e.r. (Chiralpak IB, Hexane/2-PrOH = 80/20, 0.6 mL/min), Rt (major) =18.1 min, Rt (minor) =22.3min.

**(*R*)-3-((4-chlorophenyl)thio)isobenzofuran-1(3*H*)-one**

White solid, m.p. 115.3-121.1 ̊C, 92.65% yield.

[α] ^28^_D_. = -210.6 (c = 0.330 in CHCl_3_)

^1^H NMR (500 MHz, CDCl_3_) δ 7.79 (d, *J* = 7.6 Hz, 1H), 7.73 – 7.68 (m, 1H), 7.63 (d, *J* = 7.8 Hz, 1H), 7.51 (t, *J* = 7.4 Hz, 1H), 7.43 – 7.39 (m, 2H), 7.24 – 7.20 (m, 2H), 6.67 (s, 1H).

^13^C NMR (101 MHz, CDCl_3_) δ 168.12, 160.28, 157.88, 152.59, 152.57, 144.28, 134.79, 131.34, 126.81, 125.74, 123.80, 118.89, 118.81, 116.41, 116.18, 100.19.

HRMS (ESI, m/z): calculated for C_14_H_9_ClO_2_SNa [M+Na]^+^: 298.9904, found: 298.9896.

HPLC analysis:80.7:19.3 e.r. (Chiralpak IB, Hexane/2-PrOH = 80/20, 0.6 mL/min), Rt (major) =13.9 min, Rt (minor) =15.1min.

**(*R*)-7-(4-fluorophenoxy)-[1,3]dioxolo[4,5-f]isobenzofuran-5(7*H*)-one**

White solid, m.p. 121.9-122.8 ̊C, 91.0% yield.

[α] ^28^_D_. = -236.3 (c = 0.330 in CHCl_3_)

﻿^1^H NMR (600 MHz, CDCl_3_) δ 7.21 (s, 1H), 7.15 (dd, *J* = 9.0, 4.6 Hz, 2H), 7.03 (d, *J* = 8.3 Hz, 2H), 7.01 (d, *J* = 4.7 Hz, 2H), 6.60 (s, 1H), 6.16 (d, *J* = 2.4 Hz, 2H).

﻿^13^C NMR (151 MHz, Chloroform-d) δ 167.58, 159.15 (d, *J* = 241.3 Hz), 154.14, 152.55, 150.93, 140.86, 120.99, 118.97 (d, *J* = 8.5 Hz), 116.33 (d, *J* = 23.1 Hz), 104.47, 103.56, 103.11, 99.49.

HRMS (ESI, m/z): calculated for C_15_H_9_FO_5_Na [M+Na]^+^: 311.0326, found: 311.0319.

HPLC analysis:96.1:3.9 e.r. (Chiralpak IB, Hexane/2-PrOH = 80/20, 0.6 mL/min), Rt (major) =16.5 min, Rt (minor) =18.2min.

**(*R*)-3-(4-fluorophenoxy)-5,6-dimethoxyisobenzofuran-1(3*H*)-one**

White solid, m.p. 119.9-120.8 ̊C, 85% yield.

[α] ^28^_D_. = -257.1 (c = 0.330 in CHCl_3_)

﻿^1^H NMR (600 MHz, CDCl_3_) δ 7.29 (s, 1H), 7.21 – 7.14 (m, 2H), 7.08 (s, 1H), 7.05 – 7.01 (m, 2H), 6.66 (s, 1H), 4.01 (s, 3H), 3.95 (s, 2H).

﻿^13^C NMR (151 MHz, Chloroform-d) δ 168.35, 159.11 (d, *J* = 242.6 Hz), 155.32, 152.67, 152.12, 138.75, 119.08, 118.88 (d, *J* = 8.6 Hz), 116.32 (d, *J* = 23.2 Hz), 106.14, 105.00, 99.69, 56.66, 56.53.

HRMS (ESI, m/z): calculated for C_16_H_13_FO_5_Na [M+Na]^+^: 327.0639, found: 327.0632.

HPLC analysis:91.0:9.0 e.r. (Chiralpak IB, Hexane/2-PrOH = 80/20, 0.6 mL/min), Rt (major) =11.4 min, Rt (minor) =9.7min.

**(*R*)-N-(2,3-dichloro-4-((3-oxo-1,3-dihydroisobenzofuran-1-yl)oxy)phenyl)-1-methylcyclohexane-1-carboxamide**

White solid, m.p. 109.3-110.5 ̊C, 71.82% yield.

[α] ^28^_D_. = -148.7 (c = 0.330 in CHCl_3_)

^1^H NMR (500 MHz, DMSO-*d6*) δ 9.26 (s, 1H), 7.96 – 7.92 (m, 1H), 7.91 – 7.86 (m, 2H), 7.76 (ddd, *J* = 8.0, 6.5, 2.0 Hz, 1H), 7.50 (d, *J* = 9.2 Hz, 1H), 7.46 – 7.37 (m, 1H), 7.31 (s, 1H), 2.05 (d, *J* = 13.8 Hz, 3H), 1.45 (dd, *J* = 31.5, 13.7 Hz, 5H), 1.27 – 1.19 (m, 4H), 1.17 (s, 3H).

^13^C NMR (101 MHz, CDCl_3_) δ 168.12, 160.28, 157.88, 152.59, 152.57, 144.28, 134.79, 131.34, 126.81, 125.74, 123.80, 118.89, 118.81, 116.41, 116.18, 100.19.

HRMS (ESI, m/z): calculated for C_22_H_21_Cl_2_NO_4_Na [M+Na]^+^: 456.0740, found: 450.0729.

HPLC analysis: 91.7:8.2 e.r. (Chiralpak IB, Hexane/2-PrOH = 80/20, 0.6 mL/min), Rt (major) = 25.1 min, Rt (minor) = 20.1 min.

**(*R*)-3-(4-chloro-2-(5-chloro-2-hydroxybenzyl)phenoxy)isobenzofuran-1(3*H*)-one**Yellow solid, m.p. 173.5-174.9 ̊C, 70.00% yield.

[α] ^28^_D_. = -89.8 (c = 0.330 in CHCl_3_)

^1^H NMR (500 MHz, DMSO- *d6*) δ 9.76 (s, 1H), 7.93 (d, *J* = 7.7 Hz, 1H), 7.87 (td, *J* = 7.5, 1.1 Hz, 1H), 7.74 (td, *J* = 7.6, 1.0 Hz, 1H), 7.68 (d, *J* = 7.6 Hz, 1H), 7.36 (d, *J* = 1.6 Hz, 2H), 7.13 (d, J = 1.5 Hz, 1H), 7.04 (dd, *J* = 8.5, 2.7 Hz, 1H), 6.84 (d, *J* = 2.7 Hz, 1H), 6.75 (d, *J* = 8.6 Hz, 1H), 3.80 (d, *J* = 15.1 Hz, 1H), 3.70 (d, *J* = 15.2 Hz, 1H), 1.19 (s, 1H).

^13^C NMR (101 MHz, CDCl_3_) δ 168.12, 160.28, 157.88, 152.59, 152.57, 144.28, 134.79, 131.34, 126.81, 125.74, 123.80, 118.89, 118.81, 116.41, 116.18, 100.19.

HRMS (ESI, m/z): calculated for C_21_H_15_Cl_2_O_4_ [M+H] ^+^: 401.0342, found: 401.0332.

HPLC analysis: 99.6:1.3 e.r. (Chiralpak IB, Hexane/2-PrOH = 80/20, 0.6 mL/min), Rt (major) = 23.9 min, Rt (minor) = 25.5 min.

**(*R*)-(*E*)-3-(2-(3-oxo-3-phenylprop-1-en-1-yl)phenoxy)isobenzofuran-1(3*H*)-one**White solid, m.p. 149.0-151.1 ̊C, 77.67% yield.

[α] ^28^_D_. = -416.9 (c = 0.330 in CHCl_3_)

^1^H NMR (500 MHz, CHCl_3_) δ 8.06 (d, *J* = 15.8 Hz, 1H), 7.98 (d, *J* = 7.6 Hz, 1H), 7.89 (d, *J* = 7.7 Hz, 2H), 7.82 – 7.74 (m, 2H), 7.71 (t, *J* = 7.3 Hz, 2H), 7.62 – 7.56 (m, 1H), 7.56 – 7.51 (m, 1H), 7.50 – 7.45 (m, 2H), 7.42 (t, *J* = 7.6 Hz, 2H), 7.21 (t, *J* = 7.4 Hz, 1H), 6.88 (s, 1H).

^13^C NMR (101 MHz, CDCl_3_) δ 168.05, 157.07, 144.77, 144.26, 134.86, 134.63, 132.69, 131.42, 128.71, 127.48, 126.75, 125.82, 123.82, 118.98, 117.62, 110.76, 99.24.

HRMS (ESI, m/z): calculated for C_23_H_16_O_4_Na [M+Na]^+^: 379.0941, found: 379.0932.

HPLC analysis: 99.0:1.0 e.r. (Chiralpak IB, Hexane/2-PrOH = 80/20, 0.6 mL/min), Rt (major) = 5.4 min, Rt (minor) = 5.1 min.

**(*R*)-3-(2-isopropyl-5-methylphenoxy)isobenzofuran-1(3*H*)-one**White solid, m.p. 95.6-97.7 ̊C, 68.43% yield.

[α] ^28^_D_. = -219.4 (c = 0.330 in CHCl_3_)

^1^H NMR (500 MHz, CHCl_3_) δ 7.97 – 7.94 (m, 1H), 7.81 – 7.77 (m, 1H), 7.67 (t, *J* = 7.4 Hz, 2H), 7.22 (d, *J* = 1.6 Hz, 1H), 7.16 (d, *J* = 7.7 Hz, 1H), 6.93 (dd, *J* = 7.9, 1.7 Hz, 1H), 6.80 (s, 1H), 3.27 (p, *J* = 6.9 Hz, 1H), 2.37 (s, 3H), 1.17 (d, *J* = 6.9 Hz, 3H).

^13^C NMR (101 MHz, CDCl_3_) δ 158.10, 154.67, 143.66, 139.49, 135.33, 134.80, 133.86, 131.73, 125.86, 124.26, 117.31, 99.65.

HRMS (ESI, m/z): calculated for C_18_H_18_O_3_Na [M+Na]^+^: 305.1148, found: 305.1140.

HPLC analysis: 91.7:8.2 e.r. (Chiralpak IB, Hexane/2-PrOH = 80/20, 0.6 mL/min), Rt (major) = 6.9 min, Rt (minor) = 7.5 min.

**(*R*)-3-(5-isopropyl-2-methylphenoxy)isobenzofuran-1(3*H*)-one**Yellow oil, 51.20% yield.

[α] ^28^_D_. = -131.2 (c = 0.330 in CHCl_3_)

^1^HNMR (500 MHz, CHCl_3_) δ 7.95 (d, *J* = 7.6 Hz, 1H), 7.78 (td, *J* = 7.5, 1.2 Hz, 1H), 7.70 – 7.64 (m, 2H), 7.22 (d, *J* = 1.7 Hz, 1H), 7.12 (d, *J* = 7.7 Hz, 1H), 6.93 (dd, *J* = 7.6, 1.7 Hz, 1H), 6.81 (s, 1H), 2.92 (hept, *J* = 6.9 Hz, 1H), 2.23 (s, 3H), 1.26 (dd, *J* = 6.9, 1.3 Hz, 6H).

^13^C NMR (101 MHz, CDCl_3_) δ 168.05, 157.07, 144.77, 144.26, 134.86, 134.63, 132.69, 131.42, 128.71, 127.48, 126.75, 125.82, 123.82, 118.98, 117.62, 110.76, 99.24.

HRMS (ESI, m/z): calculated for C_18_H_18_O_3_Na [M+Na]^+^: 305.1148, found: 305.1140.

HPLC analysis: 97.0:2.9 e.r. (Chiralpak IB, Hexane/2-PrOH = 80/20, 0.6 mL/min), Rt (major) = 6.9min, Rt (minor) = 7.5 min.

**(*R*)-6-methoxy-7-((3-oxo-1,3-dihydroisobenzofuran-1-yl)oxy)-2*H*-chromen-2-one**

White solid, m.p. 215.9-217.7 ̊C, 83.30% yield.

[α] ^28^_D_. = -457.2 (c = 0.330 in CHCl_3_)

^1^H NMR (500 MHz, CDCl_3_)δ 7.95 – 7.93 (m, 1H), 7.80 – 7.77 (m, 2H), 7.67 (ddd, *J* = 8.1, 6.1, 2.2 Hz, 1H), 7.64 (d, *J* = 9.5 Hz, 1H), 7.34 (s, 1H), 6.95 (s, 1H), 6.88 (s, 1H), 6.37 (d, *J* = 9.6 Hz, 1H), 3.91 (s, 3H).

^13^C NMR (101 MHz, CDCl_3_) δ 168.12, 160.28, 157.88, 152.59, 152.57, 144.28, 134.79, 131.34, 126.81, 125.74, 123.80, 118.89, 118.81, 116.41, 116.18, 100.19.

HRMS (ESI, m/z): calculated for C_18_H_13_O_6_ [M+H]^+^: 325.0707, found: 325.0699.

HPLC analysis: 90.7:9.3 e.r. (Chiralpak IB, Hexane/2-PrOH = 80/20, 0.6 mL/min), Rt (major) = 19.8 min, Rt (minor) = 21.9 min.

**(*R*)-3-((6-chloropyridin-2-yl)oxy)isobenzofuran-1(3*H*)-one**

White solid, m.p. 157.2-158.7 ̊C, 97.15% yield.

[α] ^28^_D_. = -141.5 (c=0.330 in CHCl_3_)

^1^H NMR (500 MHz, CDCl_3_) δ 7.94 (d, J = 7.6 Hz, 1H), 7.85 (s, 1H), 7.75 (t, *J* = 7.4 Hz, 1H), 7.67 (d, *J* = 7.6 Hz, 1H), 7.66 – 7.62 (m, 2H), 7.09 (d, *J* = 7.6 Hz, 1H), 6.76 (d, *J* = 8.0 Hz, 1H)

^13^C NMR (101 MHz, CDCl_3_) δ 168.12, 160.28, 157.88, 152.59, 152.57, 144.28, 134.79, 131.34, 126.81, 125.74, 123.80, 118.89, 118.81, 116.41, 116.18, 100.19.

HRMS (ESI, m/z): calculated for C_13_H_9_ClNO_3_ [M+H]^+^: 262.0265, found: 262.0259.

HPLC analysis: 98.9:1.1 e.r. (Chiralpak IB, Hexane/2-PrOH = 80/20, 0.6 mL/min), Rt (major) =9.7 min, Rt (minor) =12.2 min.

**(*R*)-3-((1H-indol-5-yl)oxy)isobenzofuran-1(3*H*)-one**

White solid, m.p. 186.2-189.3 ̊C, 68.63% yield.

[α] ^28^_D_. = -357.5 (c = 0.330 in CHCl_3_)

^1^H NMR (500 MHz, CDCl_3_)δ 8.18 (s, 1H), 7.93 (d, *J* = 7.6 Hz, 1H), 7.75 (q, *J* = 7.4 Hz, 2H), 7.64 (t, *J* = 7.4 Hz, 1H), 7.56 (s, 1H), 7.35 (d, *J* = 8.6 Hz, 1H), 7.10 (d, *J* = 8.7 Hz, 1H), 6.84 (s, 1H), 6.54 (s, 1H).

^13^C NMR (101 MHz, CDCl_3_) δ 168.03, 155.13, 144.18, 134.84, 131.40, 129.76, 128.92, 126.72, 125.78, 123.81, 118.54, 99.56.

HRMS (ESI, m/z): calculated for C_16_H_11_NO_3_Na [M+Na]^+^: 288.0631, found: 288.0624.

HPLC analysis: 96.7:3.3 e.r. (Chiralpak IB, Hexane/2-PrOH = 80/20, 0.6 mL/min), Rt (major) =26.1 min, Rt (minor) =28.1 min.

**IX. NMR spectra of products**

**3b:** **^1^H NMR**


**3b：^13^C NMR**

**3b:HRMS**

**3c:** **^1^H NMR**

**3c：^13^C NMR**

**3c:HRMS**

**3d:** **^1^H NMR**

**3d：^13^C NMR**

**3d:HRMS**

**3e:** **^1^H NMR**

**3e：^13^C NMR**

**3e:HRMS**

**3f:** **^1^H NMR**

**3f：^13^C NMR**

**3f:HRMS**

**3g:** **^1^H NMR**

**3g：^13^C NMR**

**3g:HRMS**

**3h:** **^1^H NMR**

**3h：^13^C NMR**

**3h:HRMS**

**3i:** **^1^H NMR**

**3i：^13^C NMR**

**3i:HRMS**

**3j:** **^1^H NMR**

**3j：^13^C NMR**

**3j:HRMS**

**3k:** **^1^H NMR**

**3k：^13^C NMR**

^^

**3k:HRMS**

**3l:** **^1^H NMR**

**3l：^13^C NMR**

**3l:HRMS**

**3m:** **^1^H NMR**

**3m：^13^C NMR**

**3m:HRMS**

**3n:** **^1^H NMR**

**3n：^13^C NMR**

**3n:HRMS**

**3o:** **^1^H NMR**

**3o：^13^C NMR**

**3o:HRM**

**3p:** **^1^H NMR**

**3p：^13^C NMR**

**3p:HRMS**

**3q:** **^1^H NMR**

**3q：^13^C NMR**

**3q:HRMS**

**3r:** **^1^H NMR**

**3r：^13^C NMR**

**3r:HRMS**

**3s:** **^1^H NMR**

**3s：^13^C NMR**

**3s:HRMS**

**3t:** **^1^H NMR**

**3t：^13^C NMR**

**3t:HRMS**

**3u:** **^1^H NMR**

**3u：^13^C NMR**

**3u:HRMS**

**3v:** **^1^H NMR**

**3v：^13^C NMR**

**3v:HRMS**

**3w:** **^1^H NMR**

**3w：^13^C NMR**

**3w:HRMS**

**3x:** **^1^H NMR**

**3x：^13^C NMR**

**3x:HRMS**

**3y:** **^1^H NMR**

**3y：^13^C NMR**

**3y:HRMS**

**3z:** **^1^H NMR**

**3z：^13^C NMR**

**3z:HRMS**

**X. HPLC spectra of products**

**Racemic 3b**


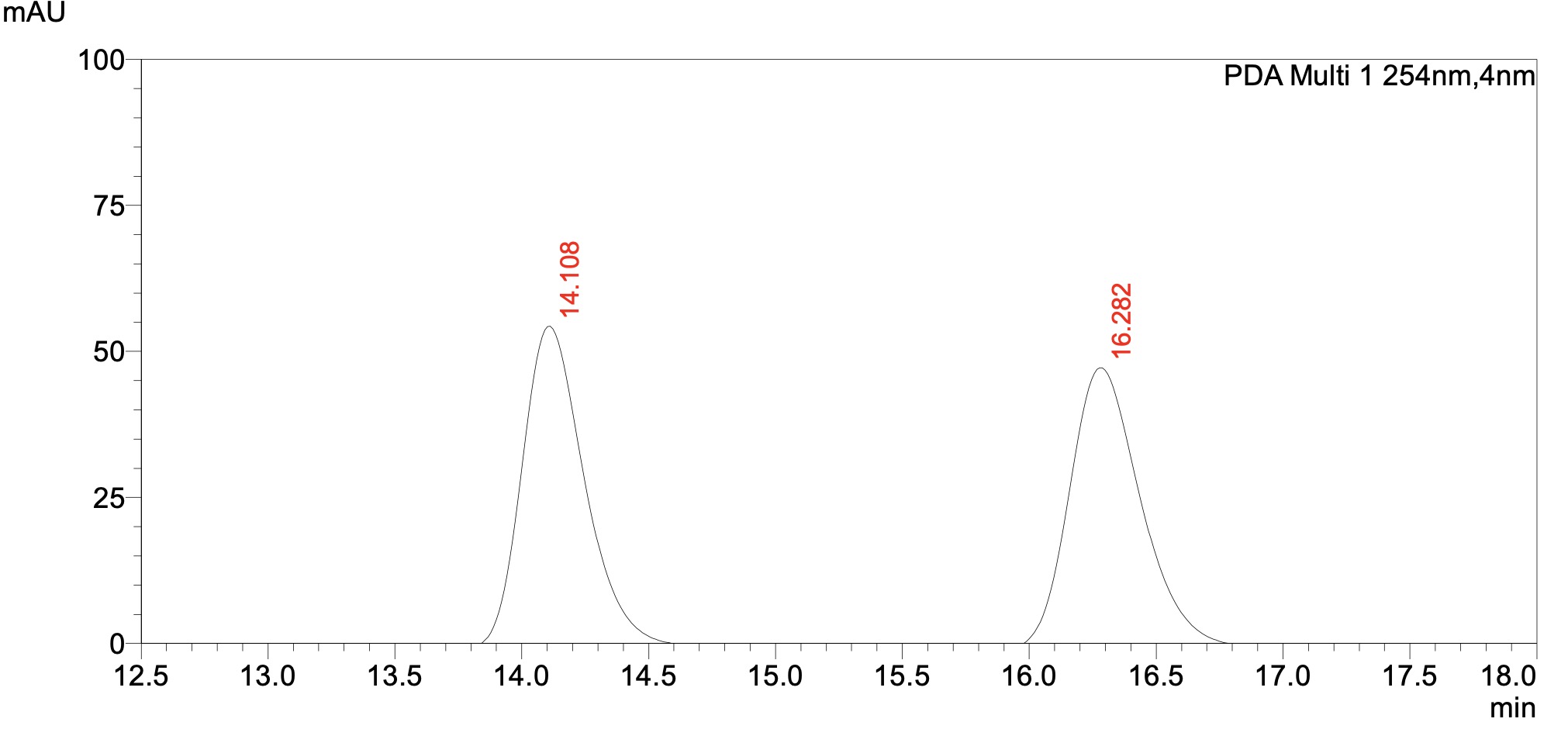


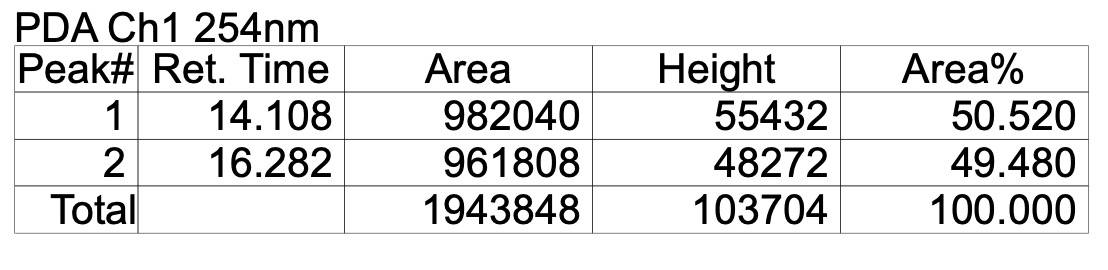


**(R)-3b**


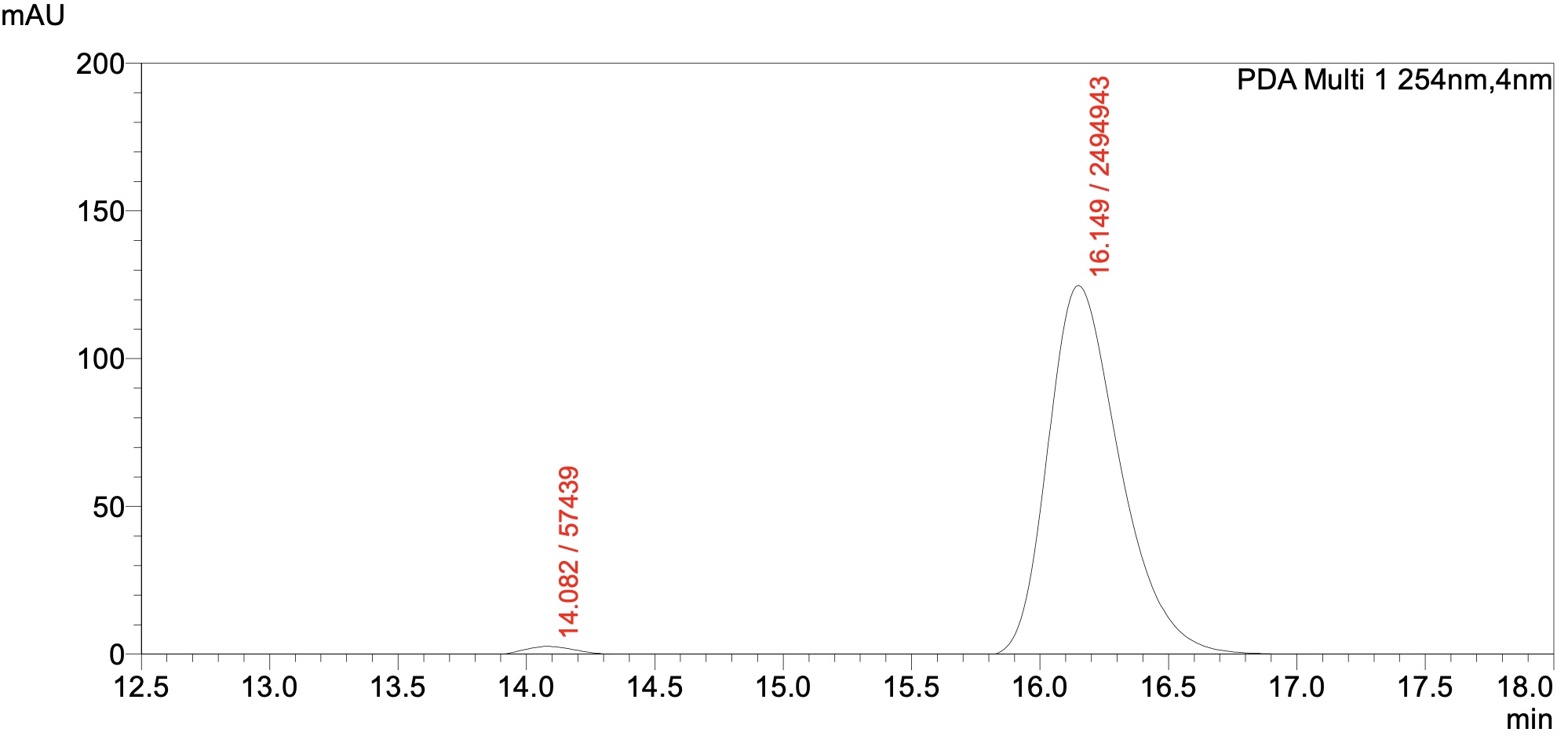


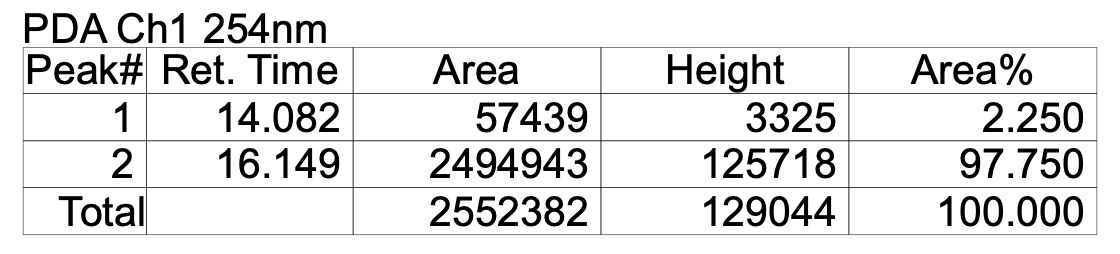


**Racemic 3c**


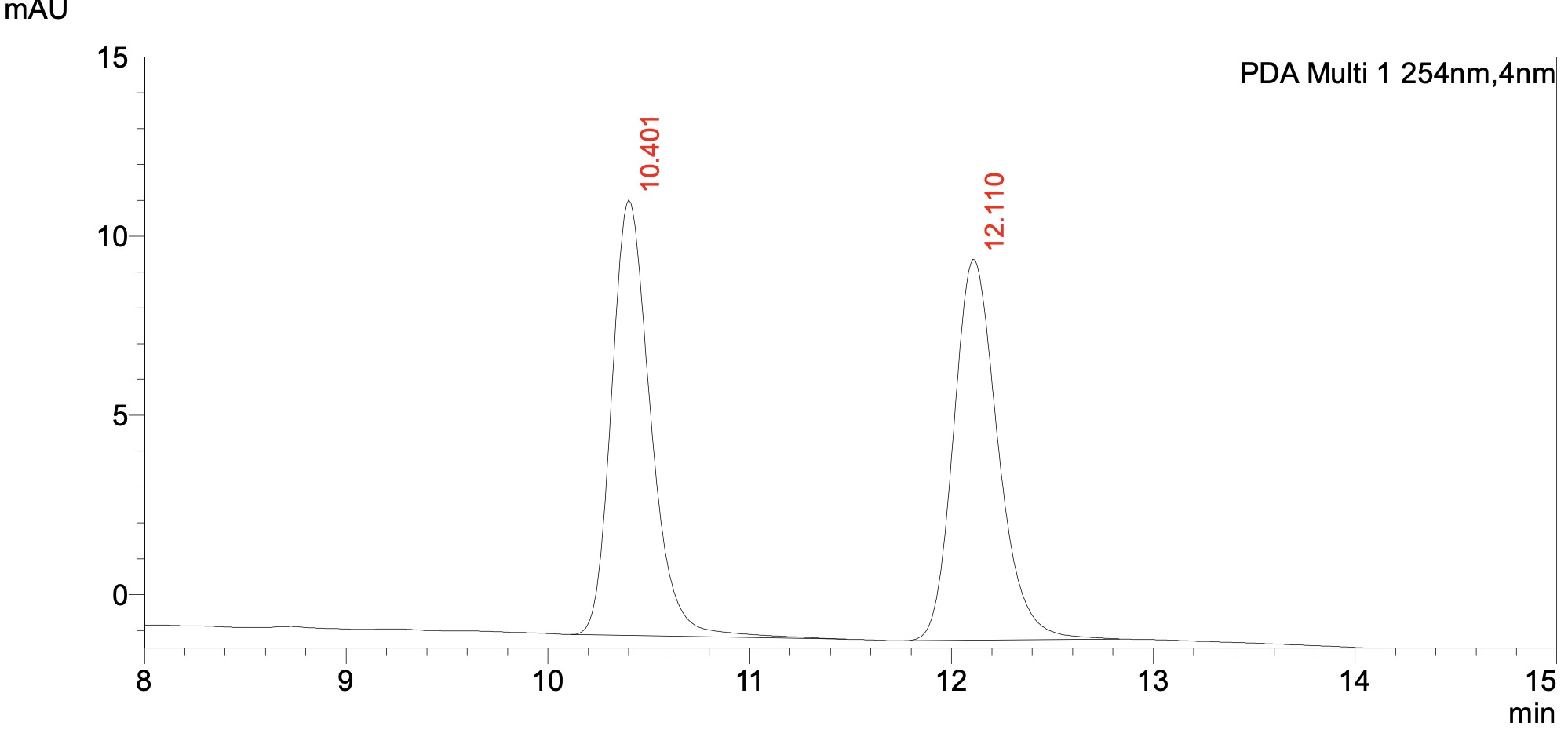


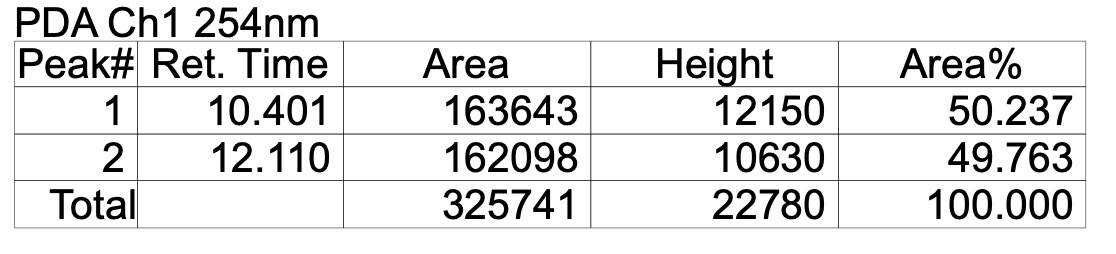


**(R)-3c**


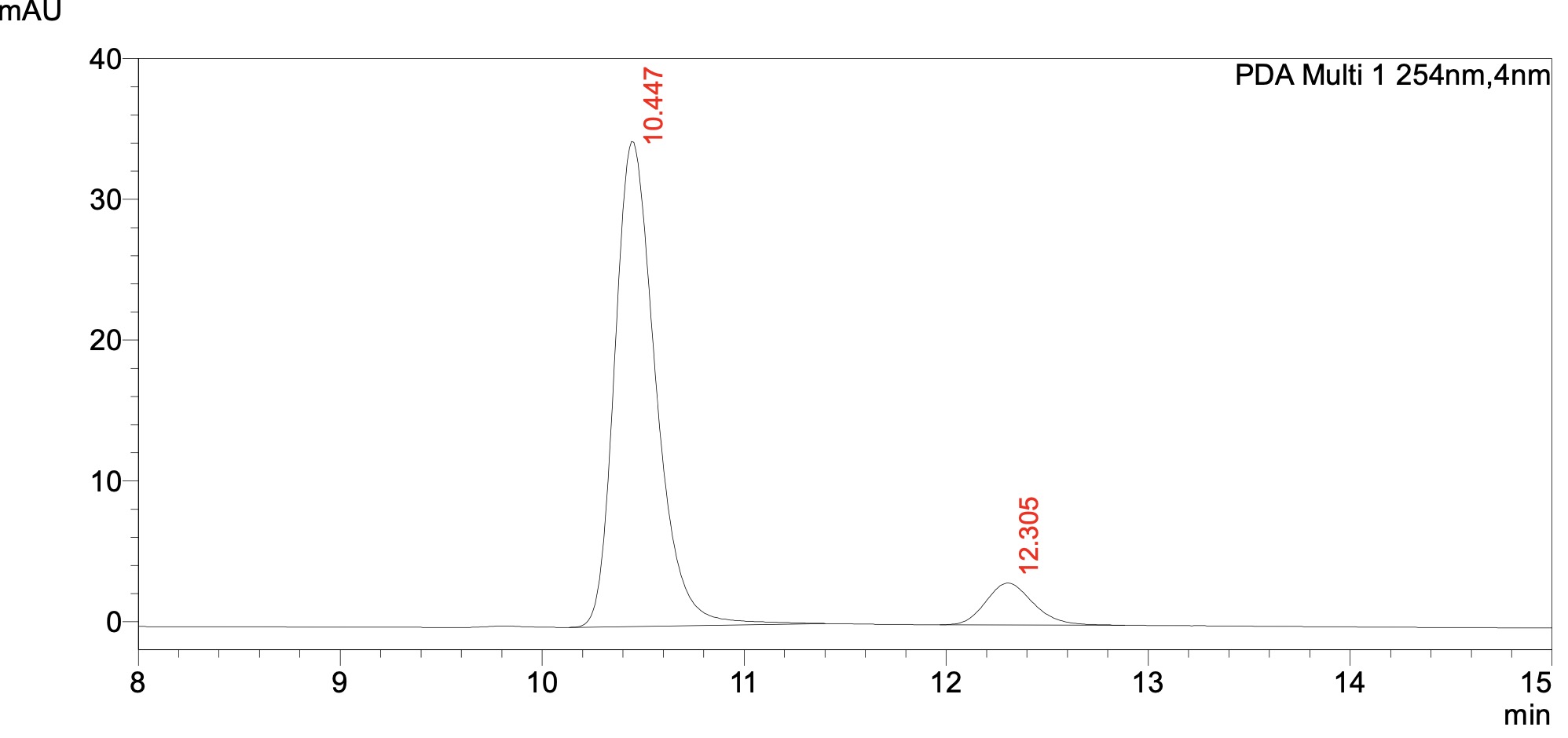

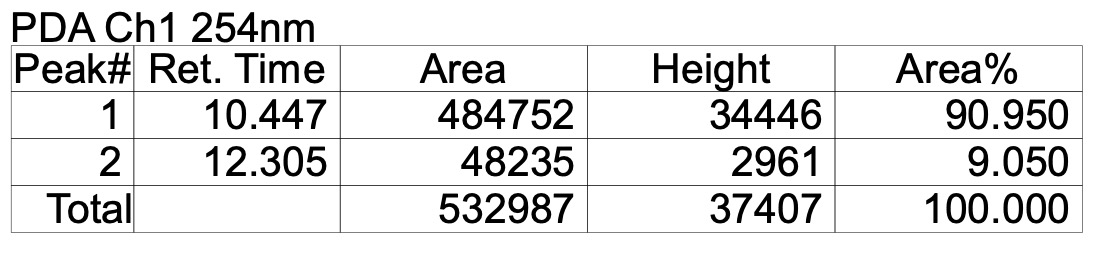


**Racemic 3d**


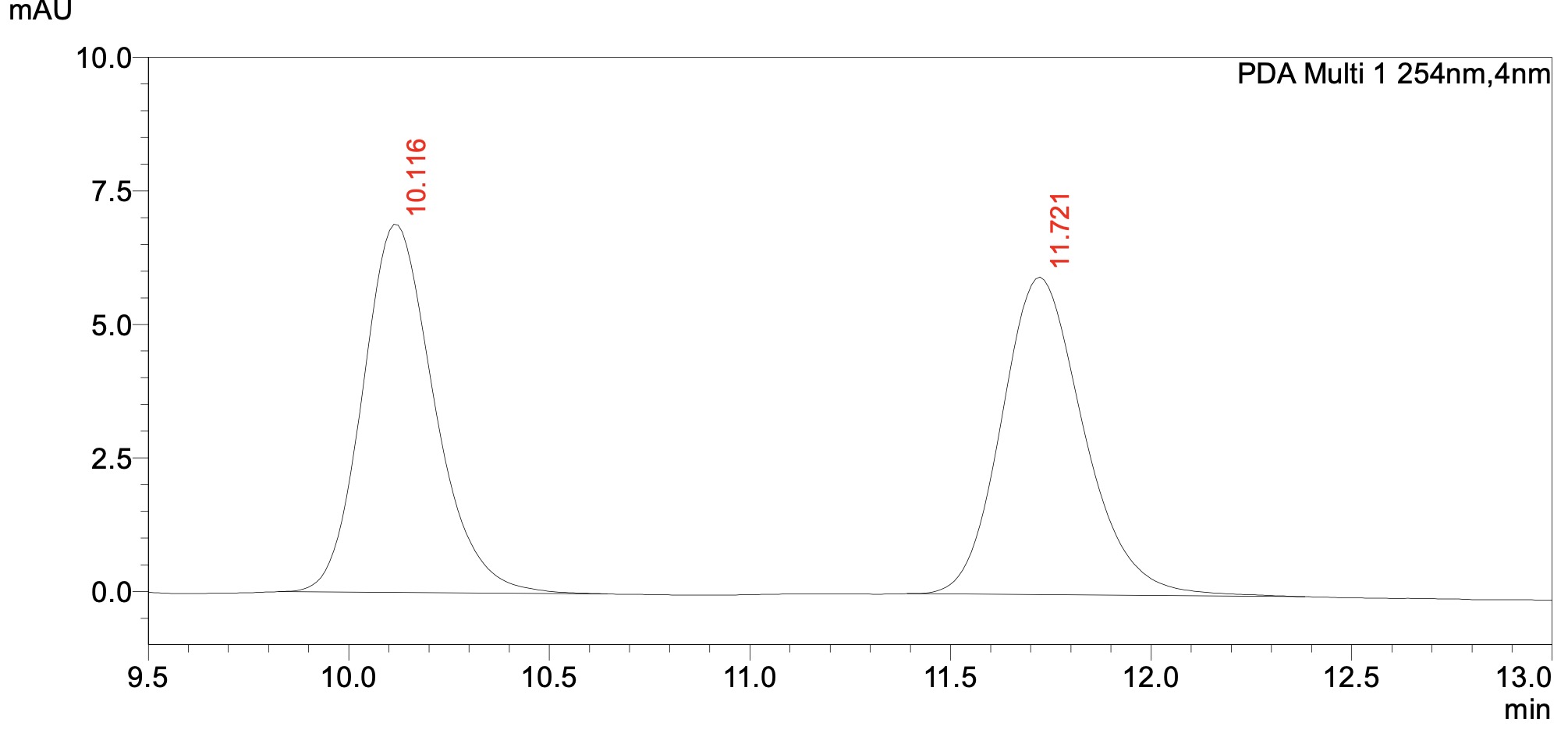


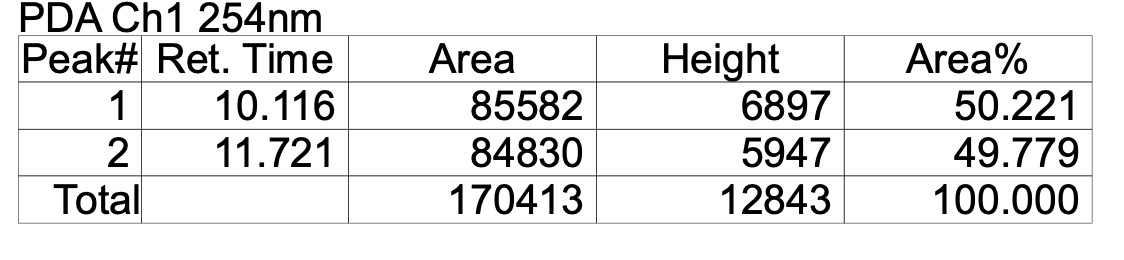


**(R)-3d**


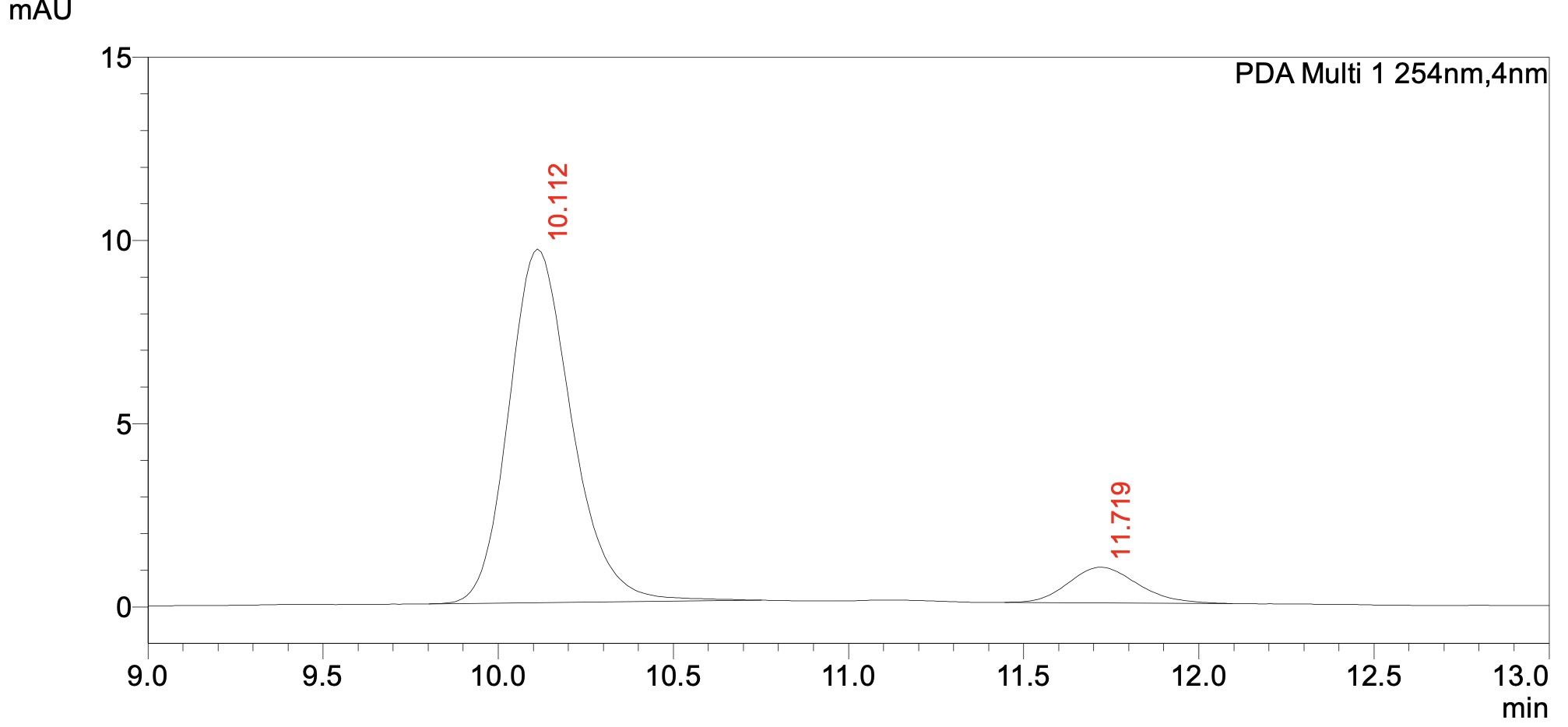


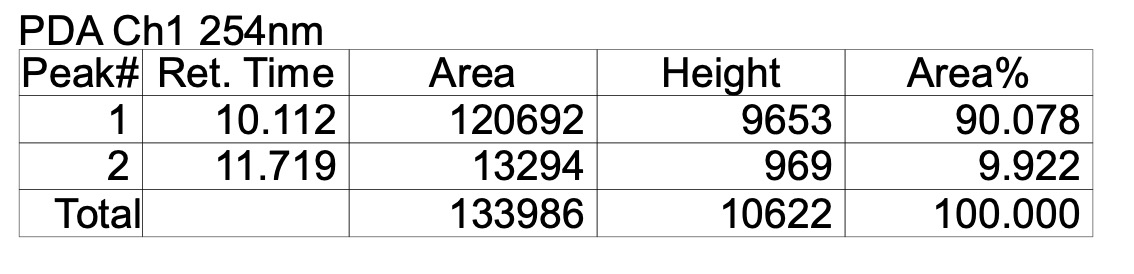


**Racemic 3e**


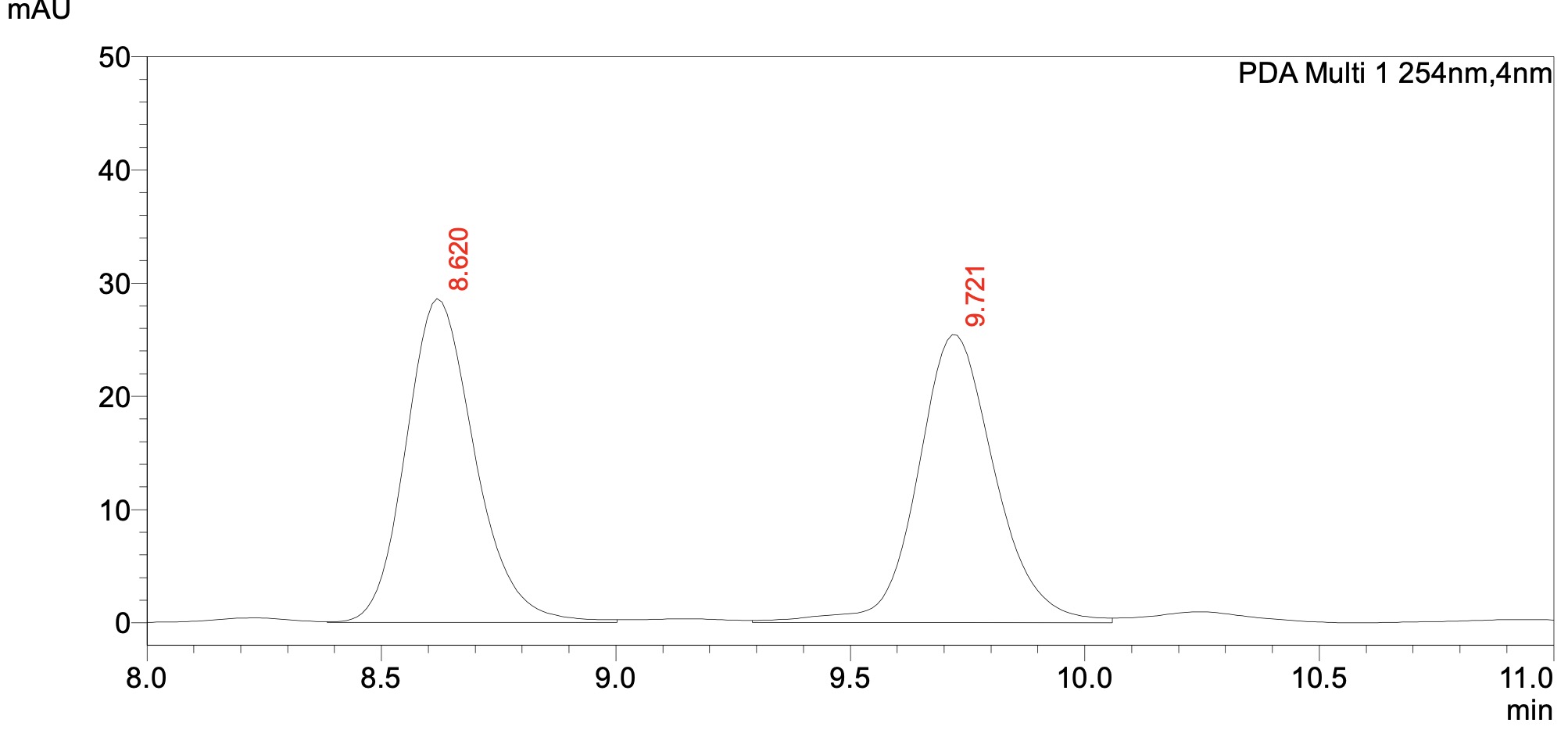


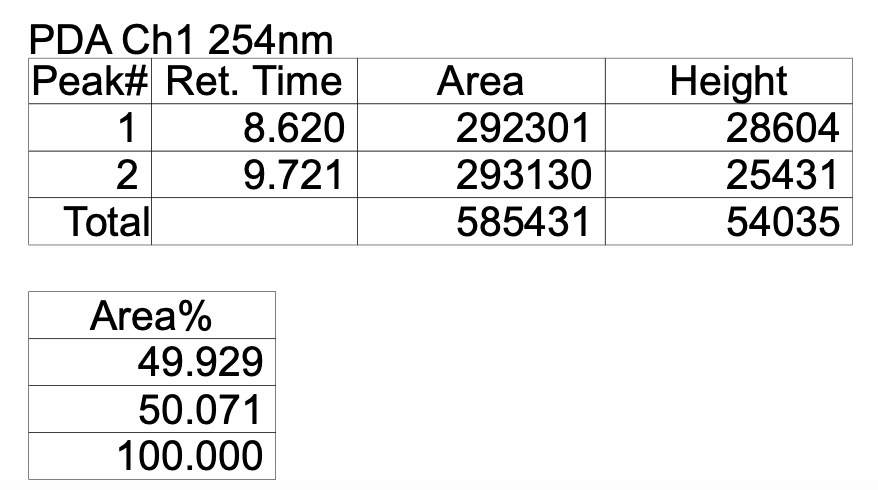


**(R)-3e**


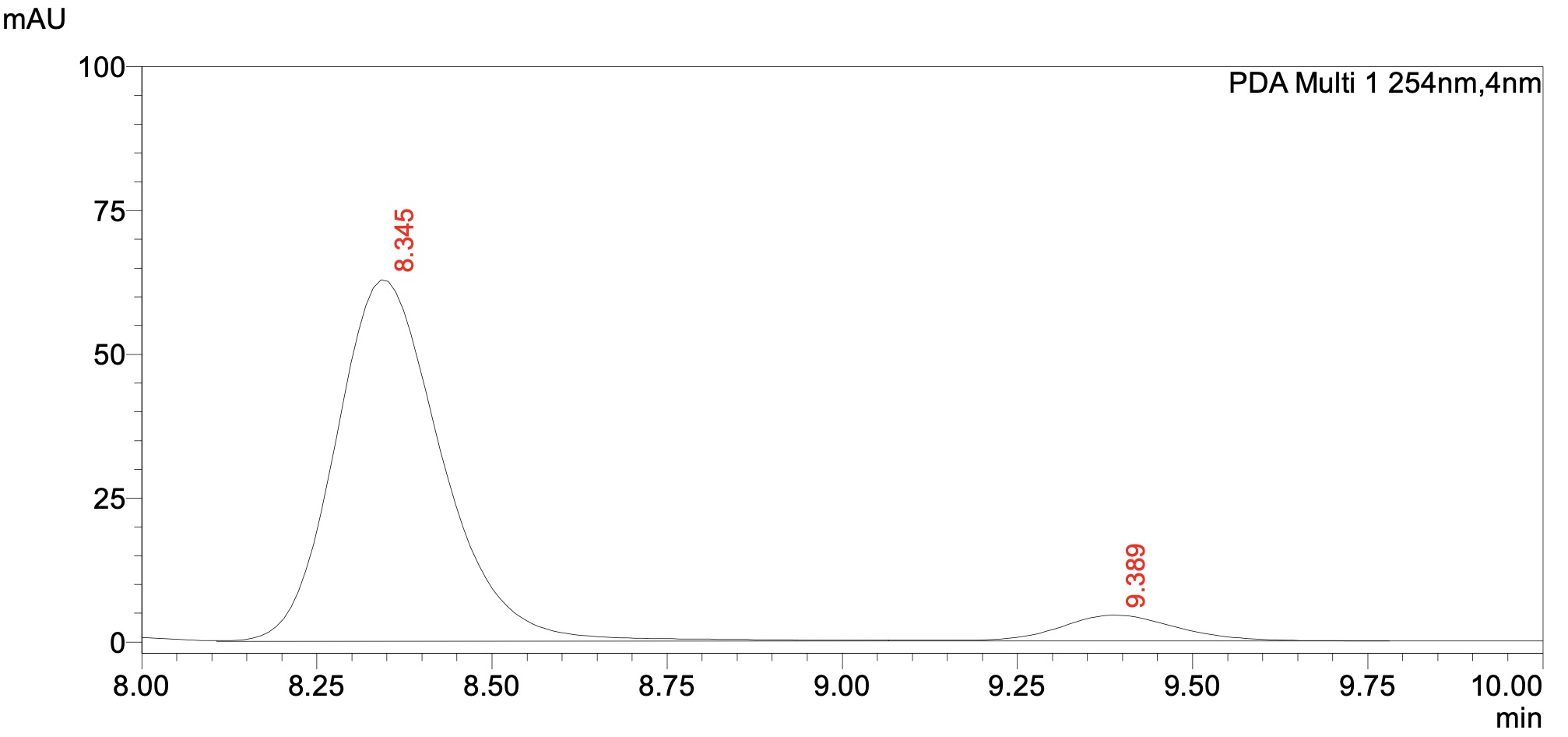


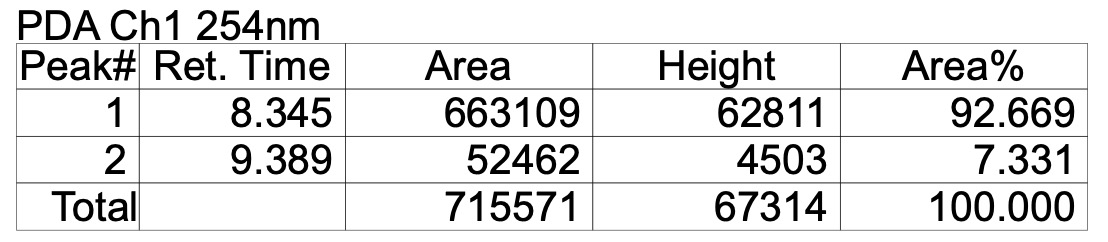


**Racemic 3f**


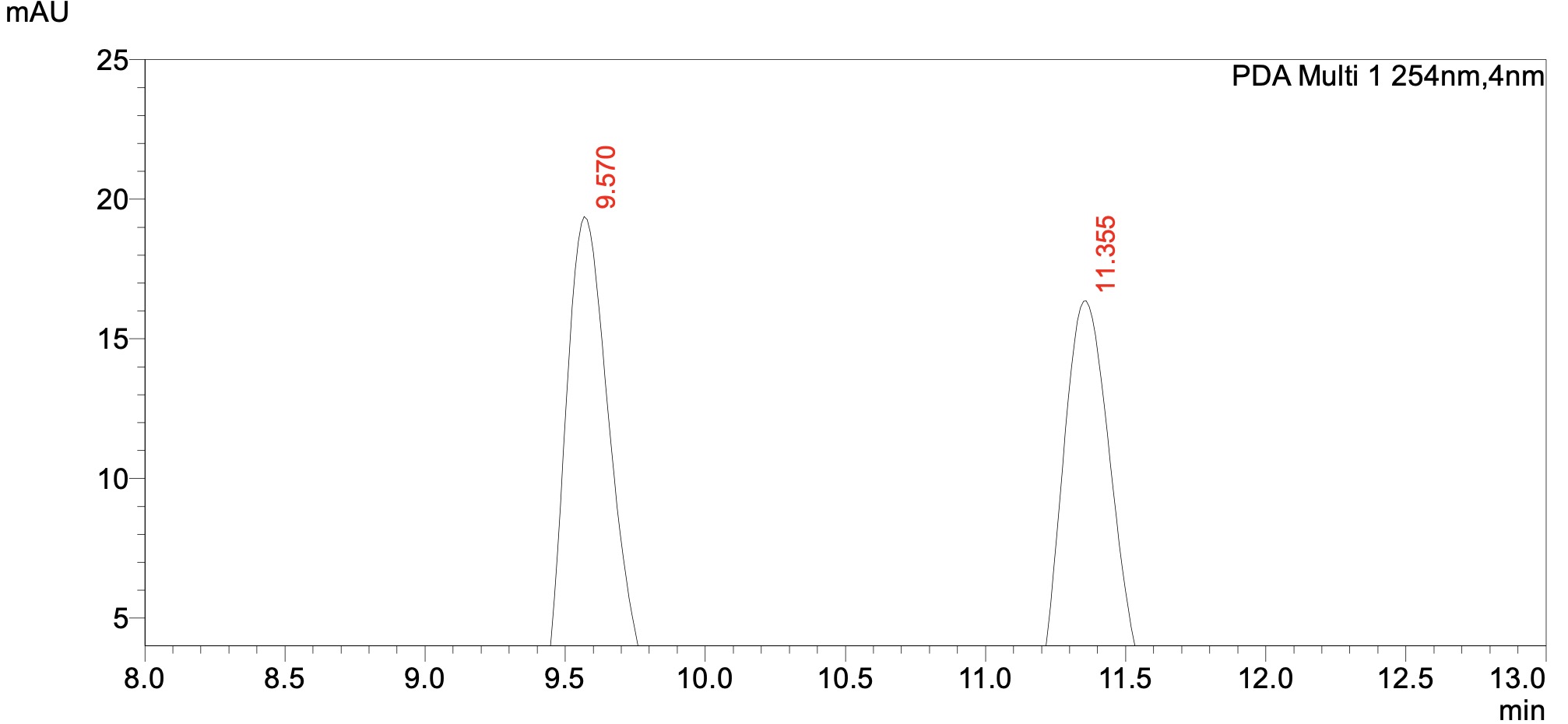


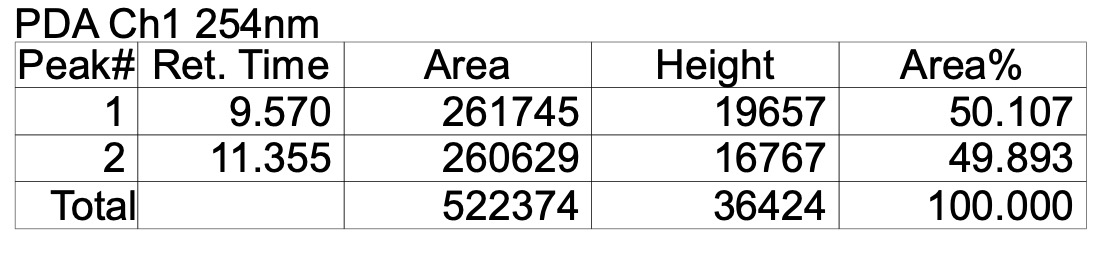


**(R)-3f**


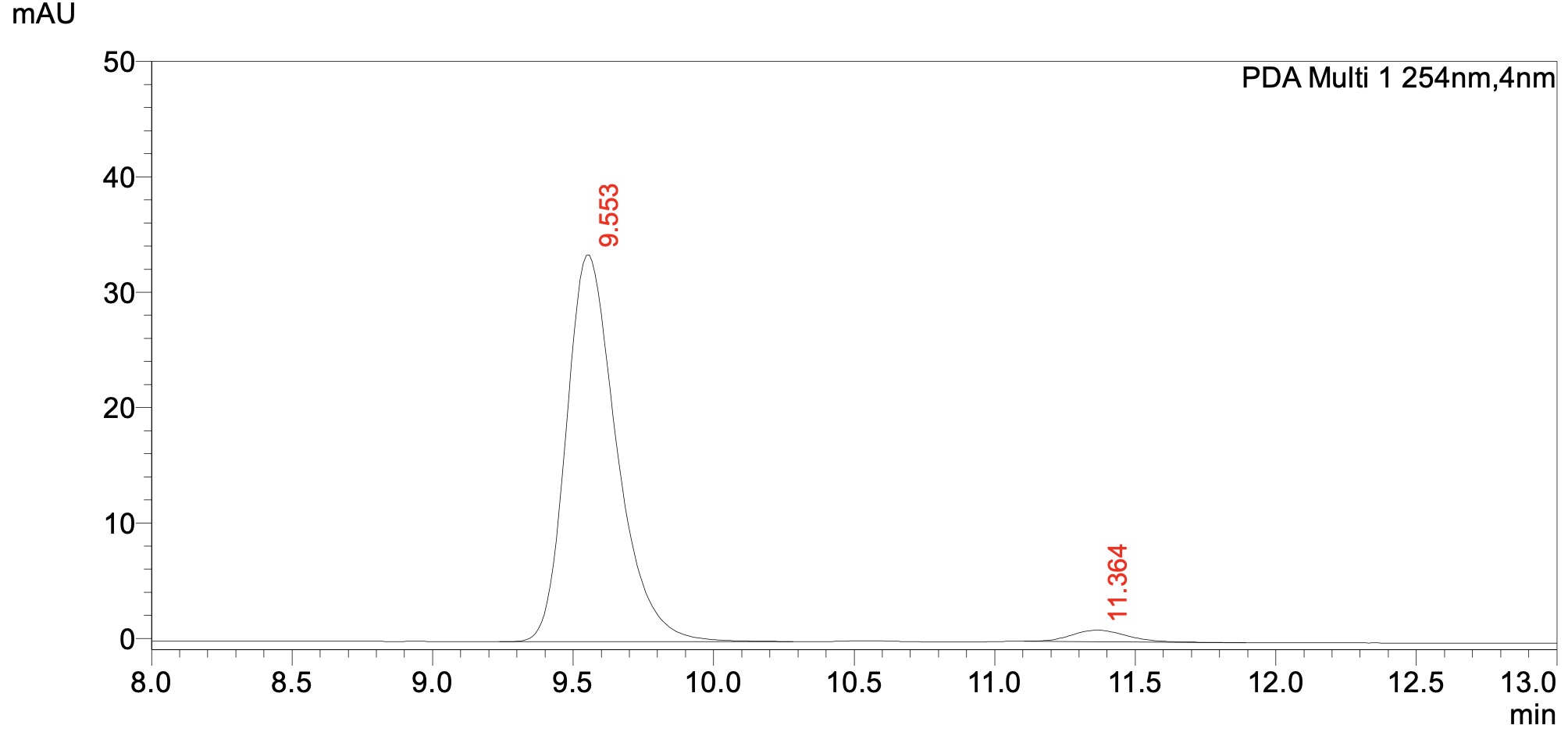


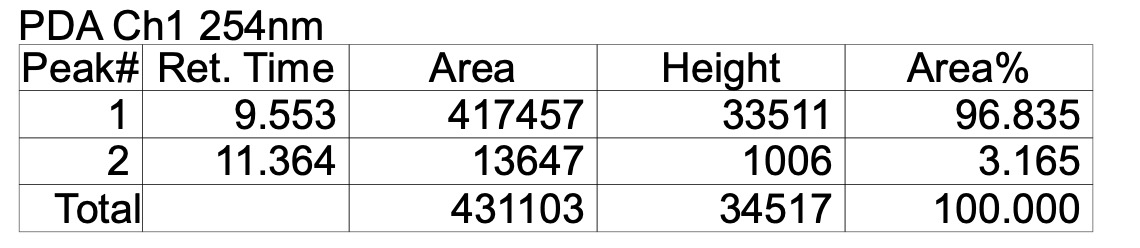


**Racemic 3g**


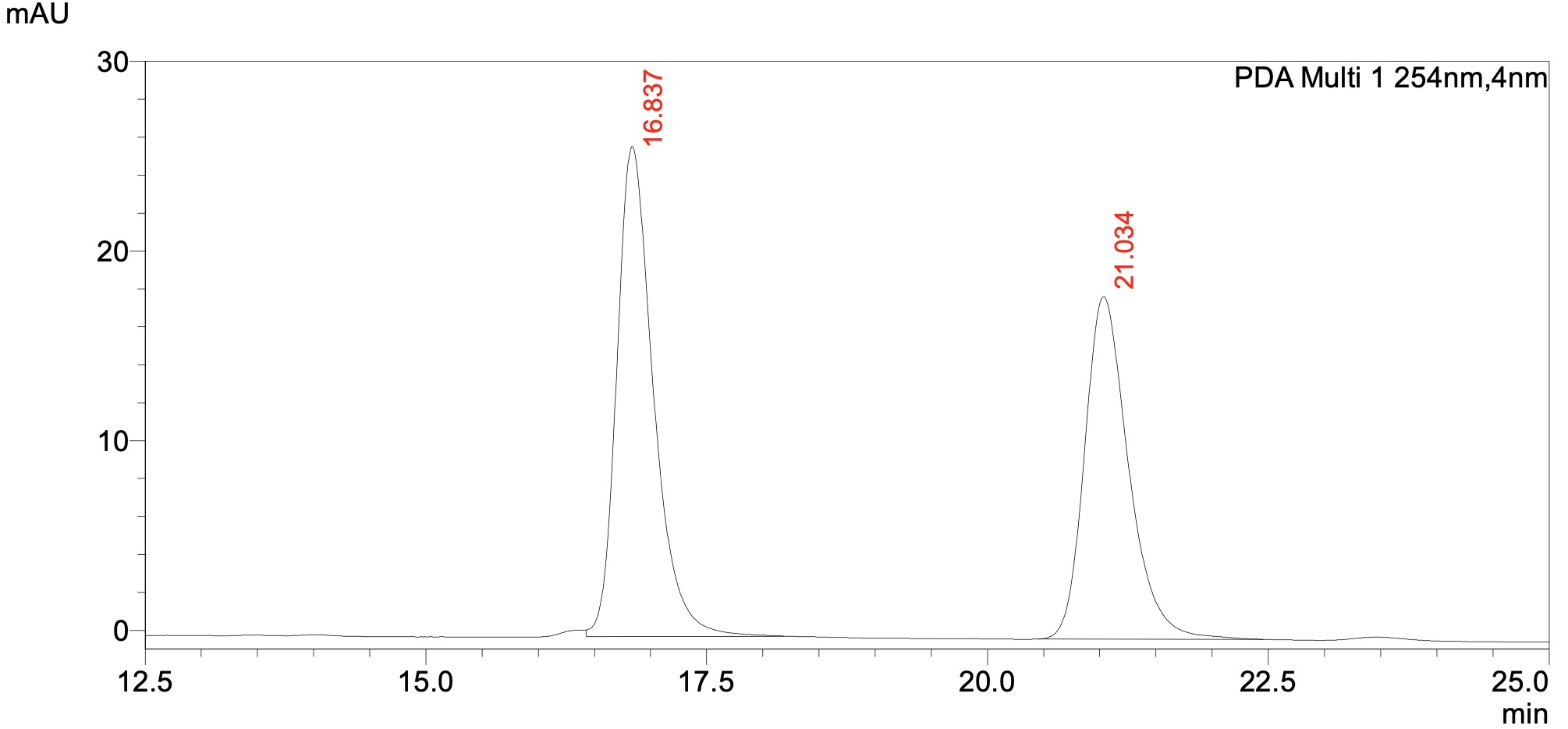

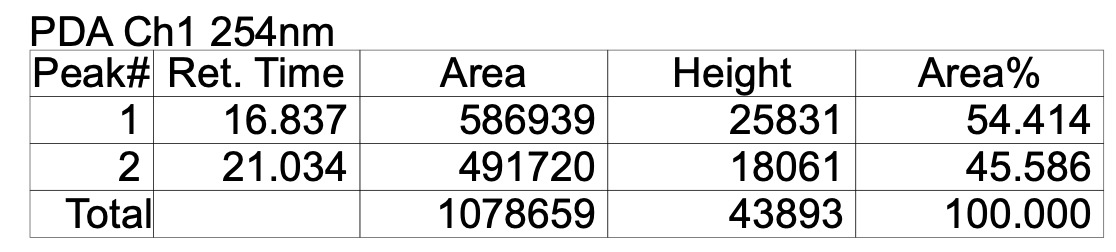


**(R)-3g**


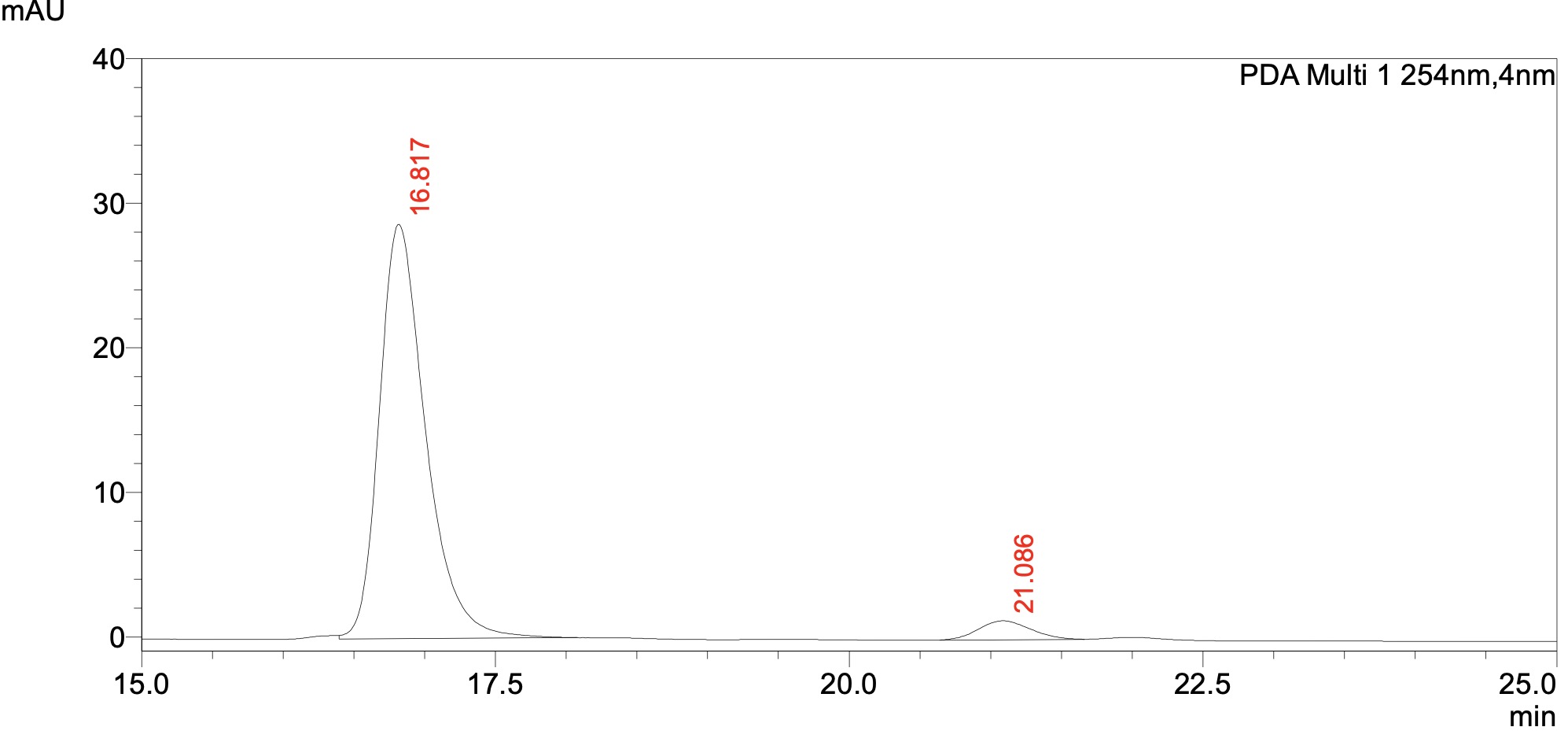


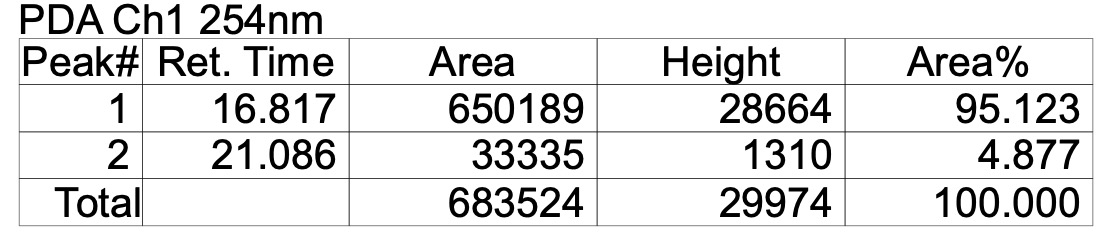


**Racemic 3h**


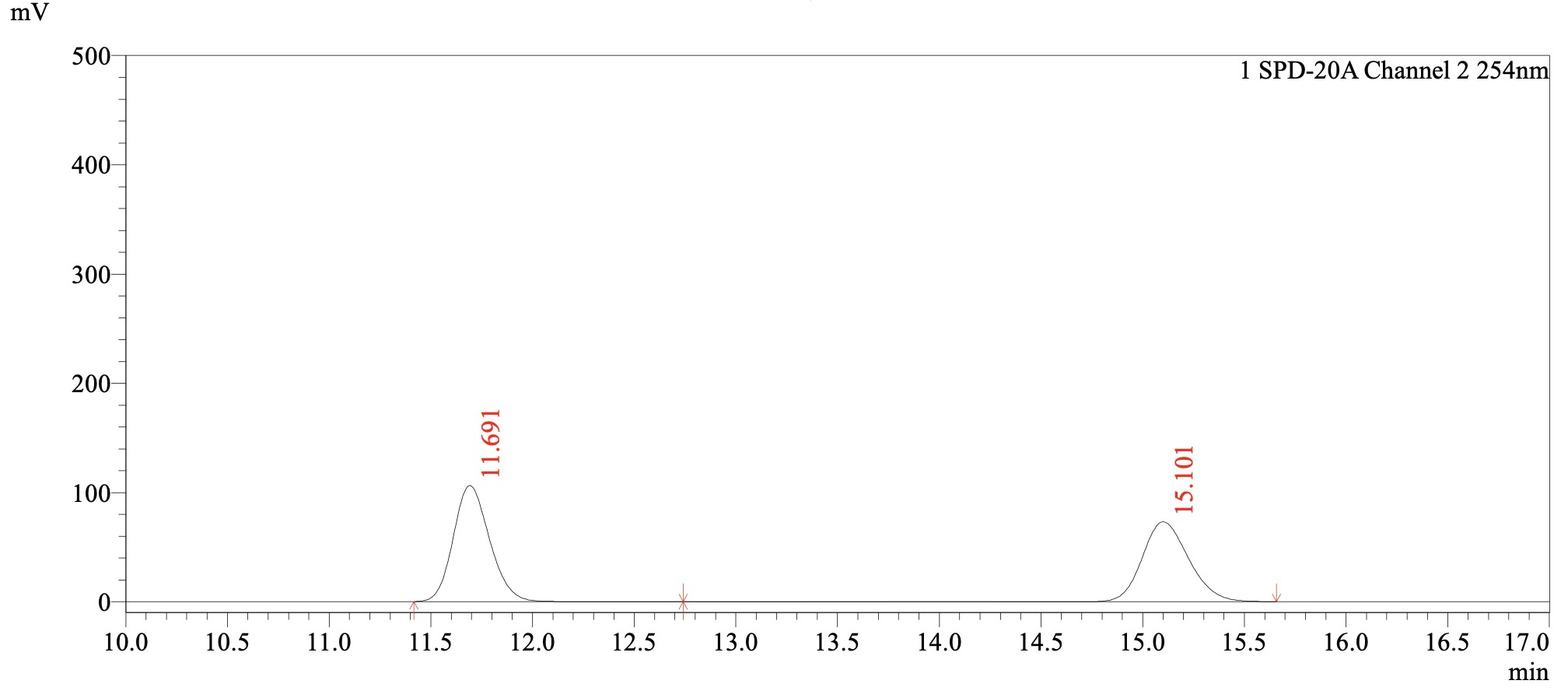


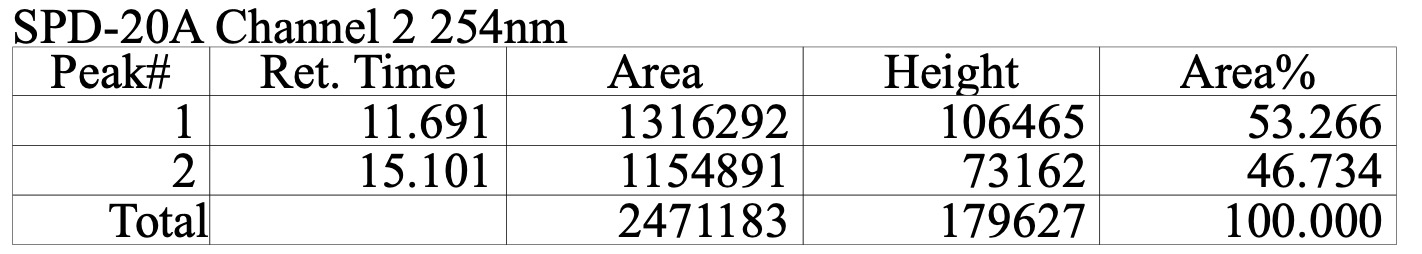


**(R)-3h**


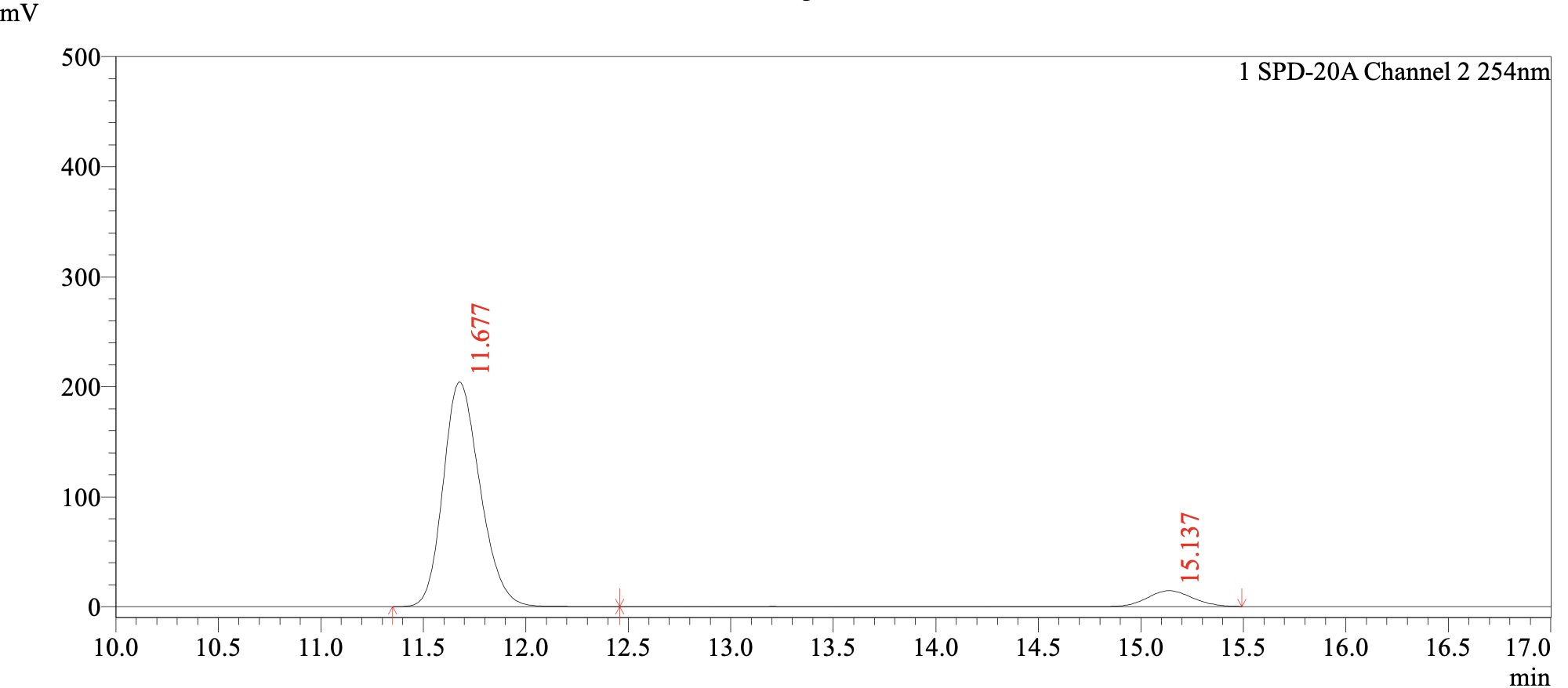


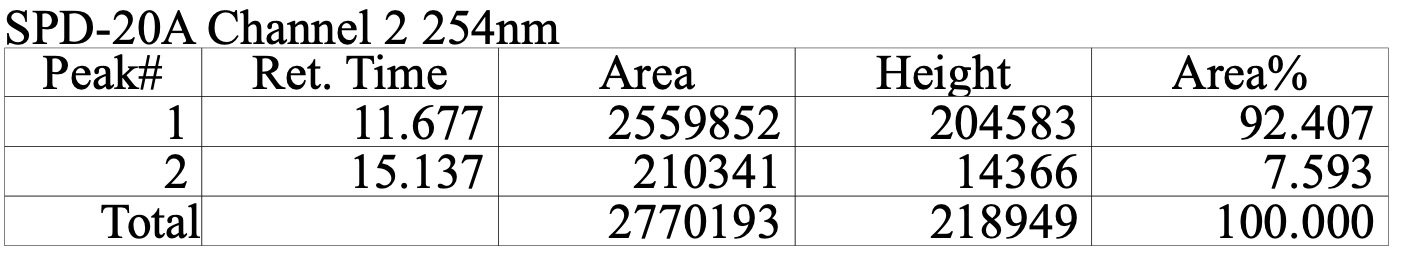


**Racemic 3i**


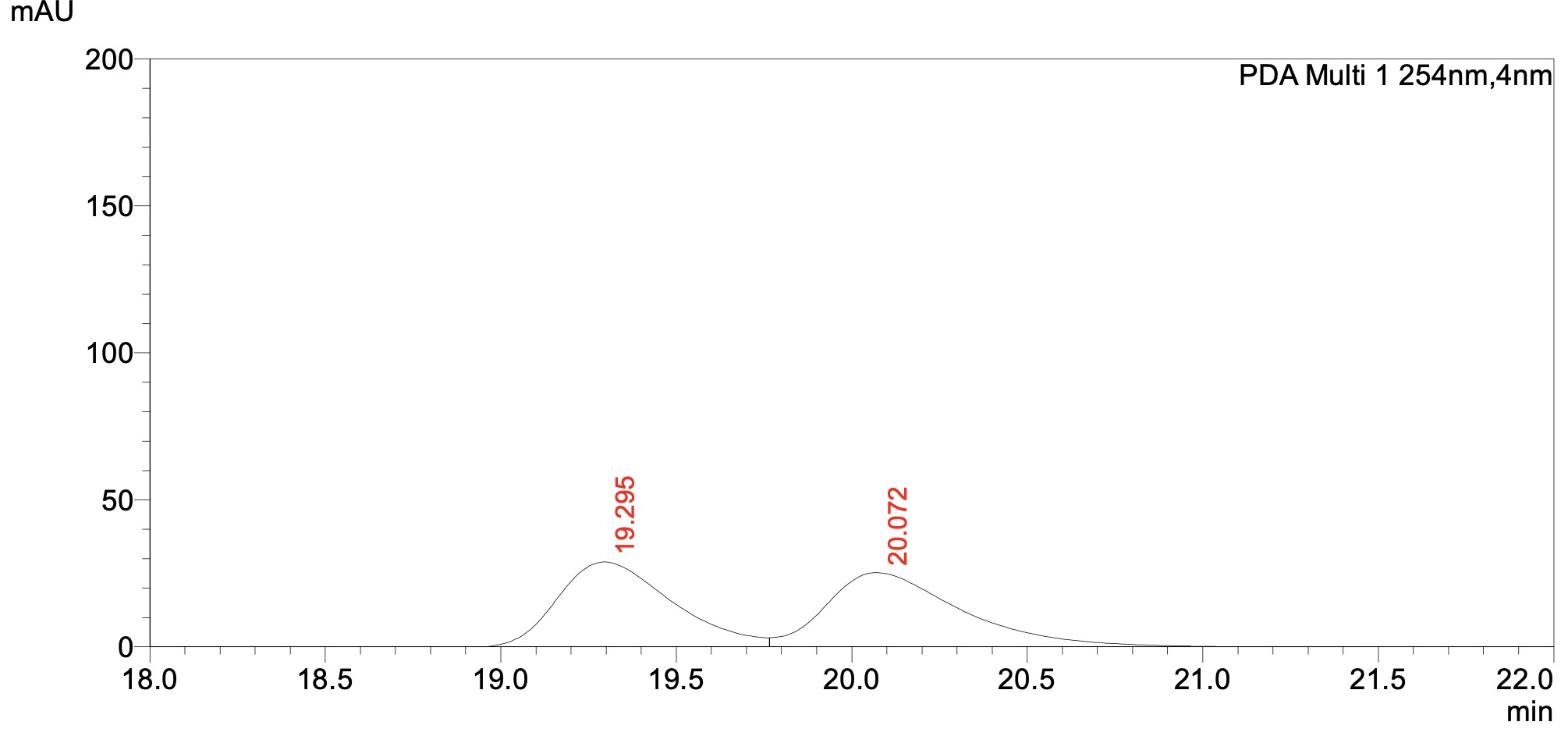


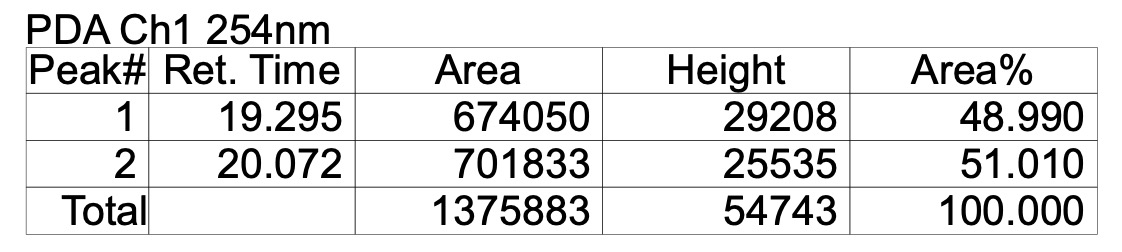


**(R)-3i**


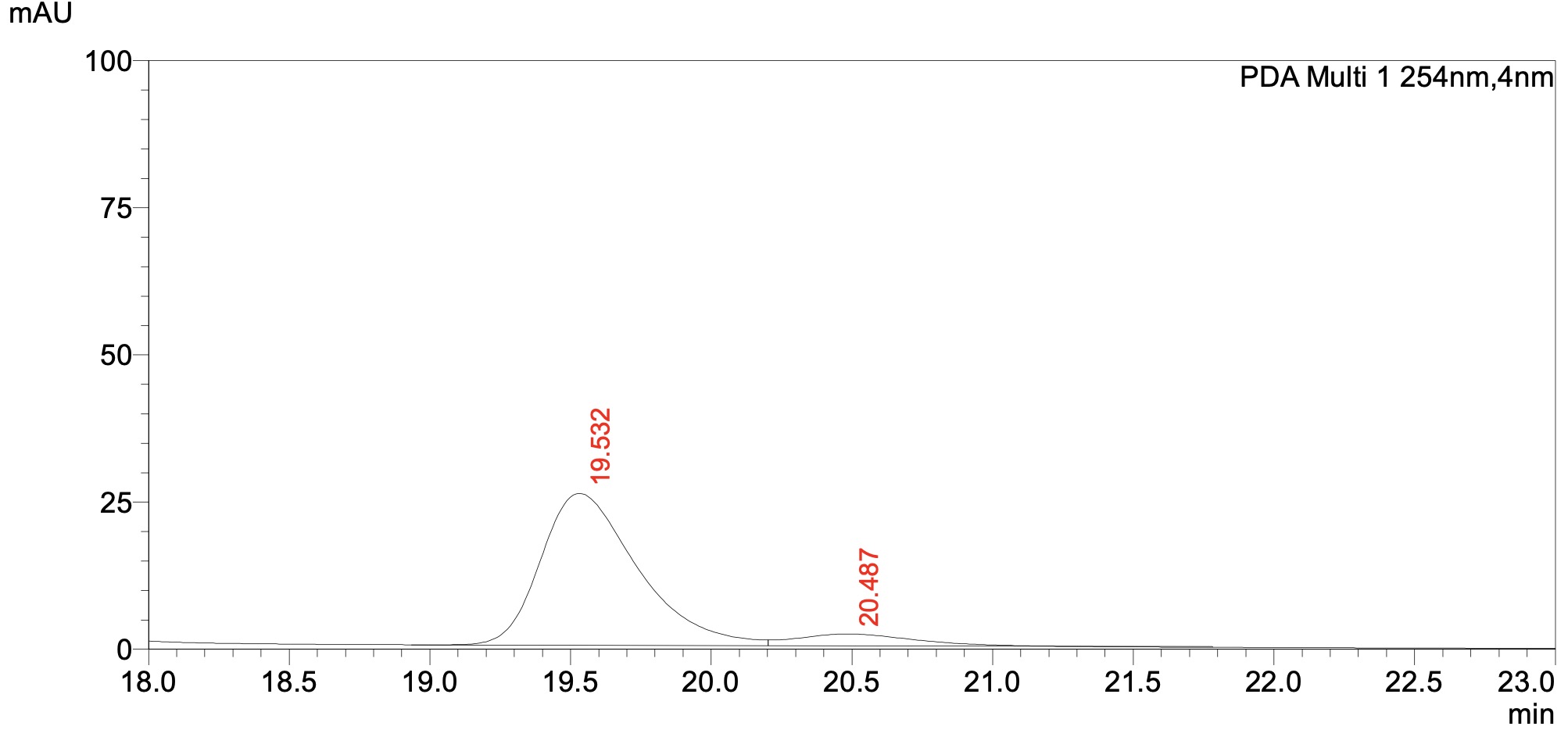


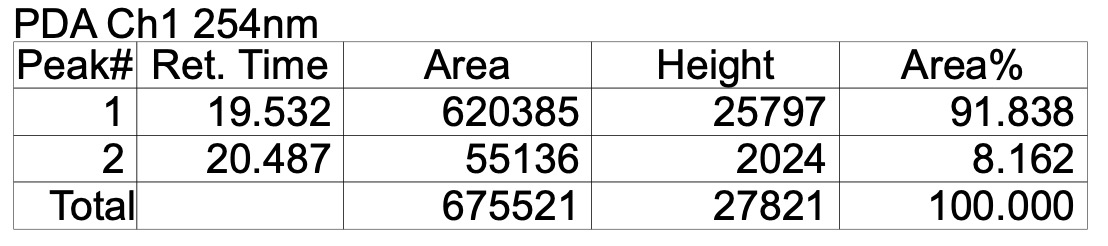


**Racemic 3j**


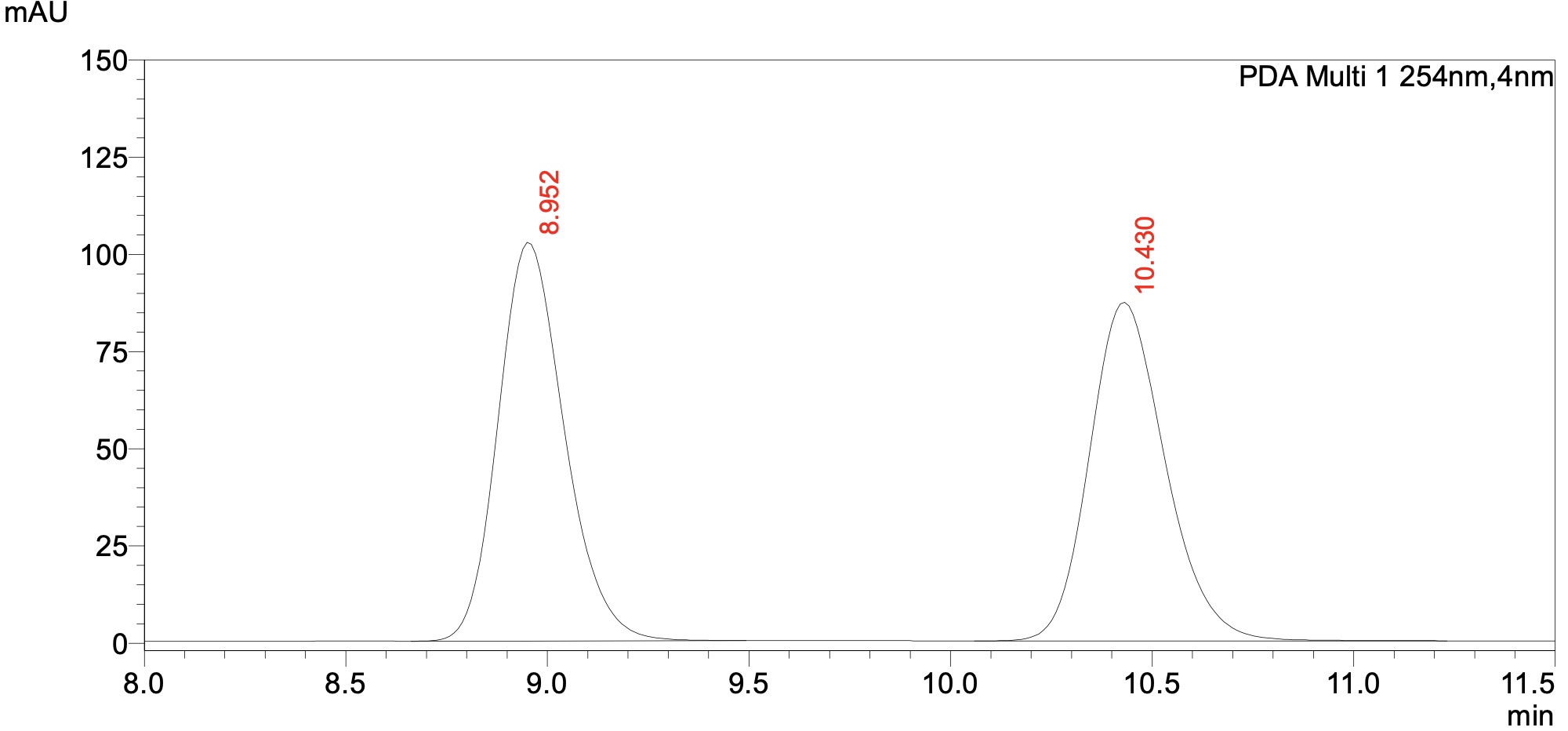


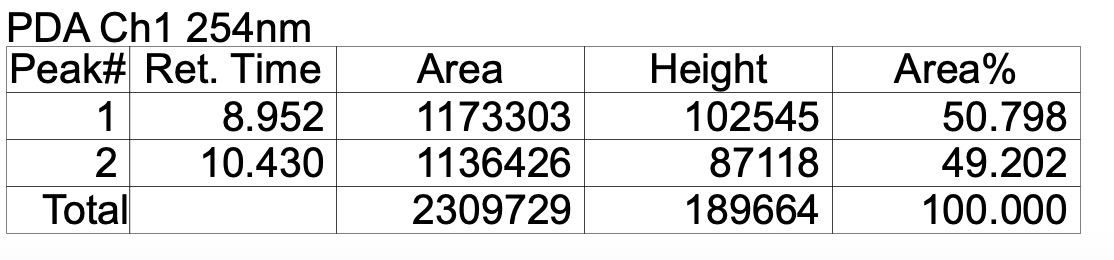


**(R)-3j**


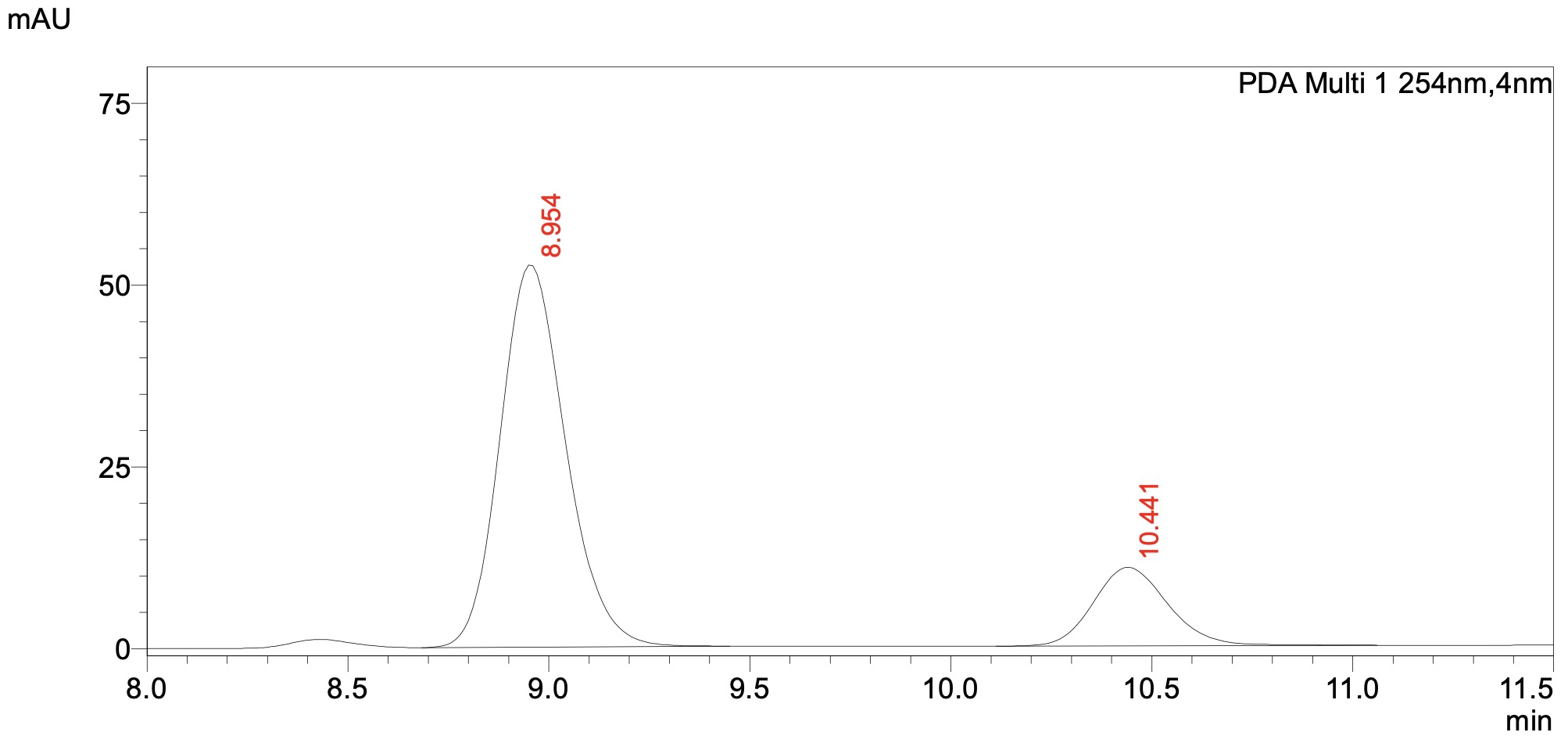


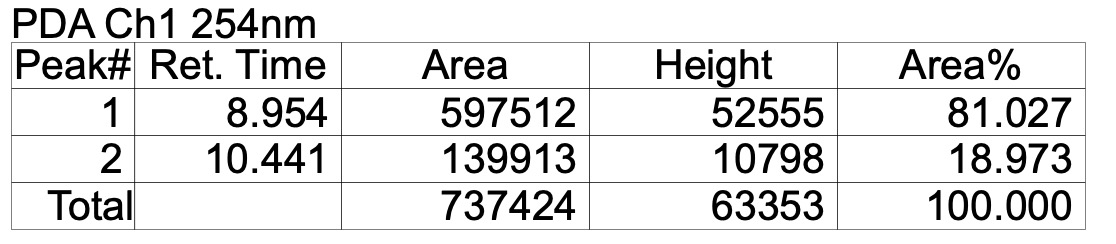


**Racemic 3k**


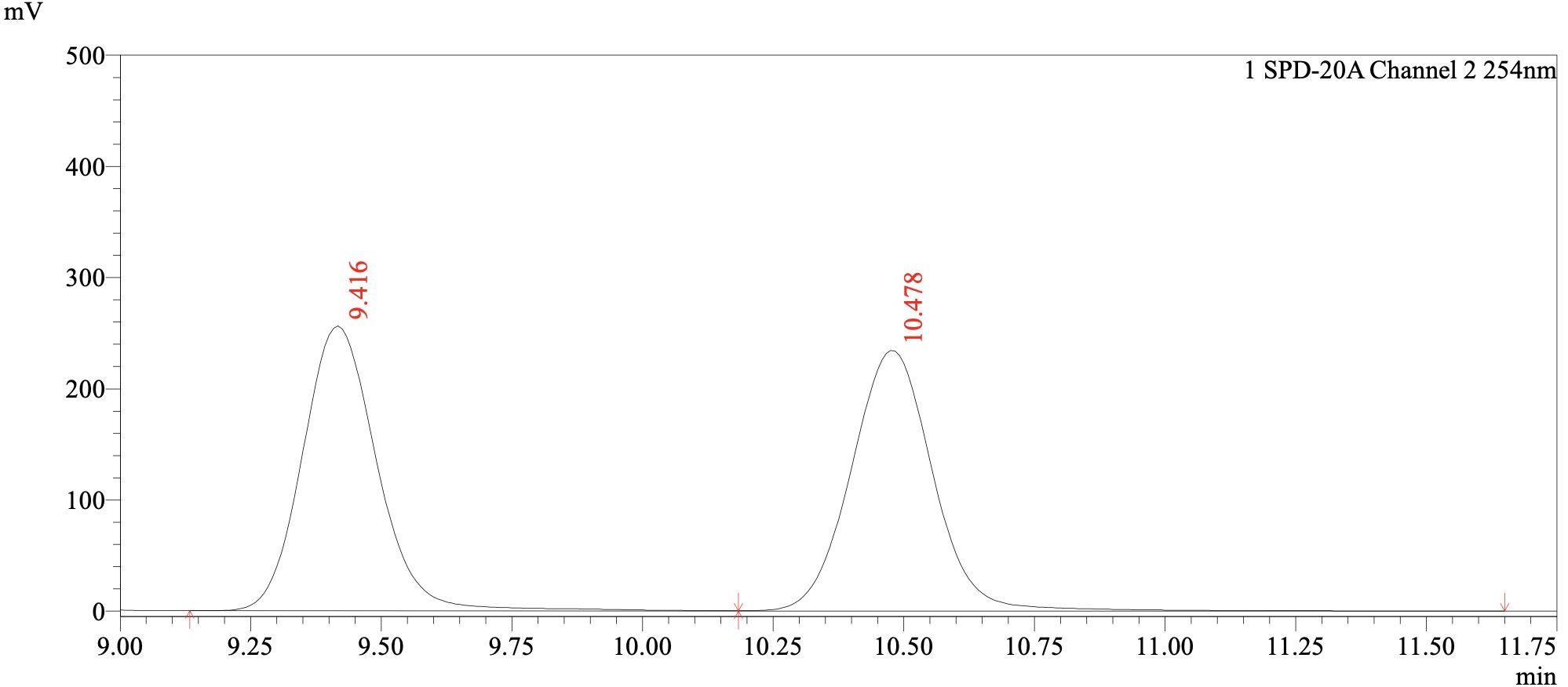


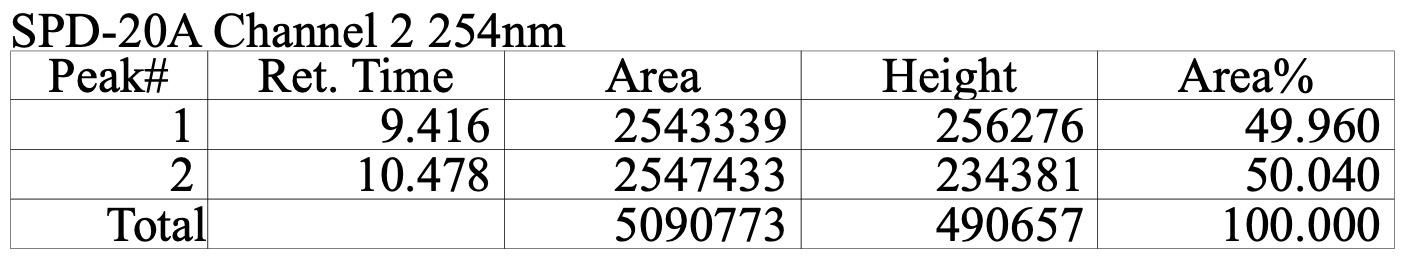


**(R)-3k**


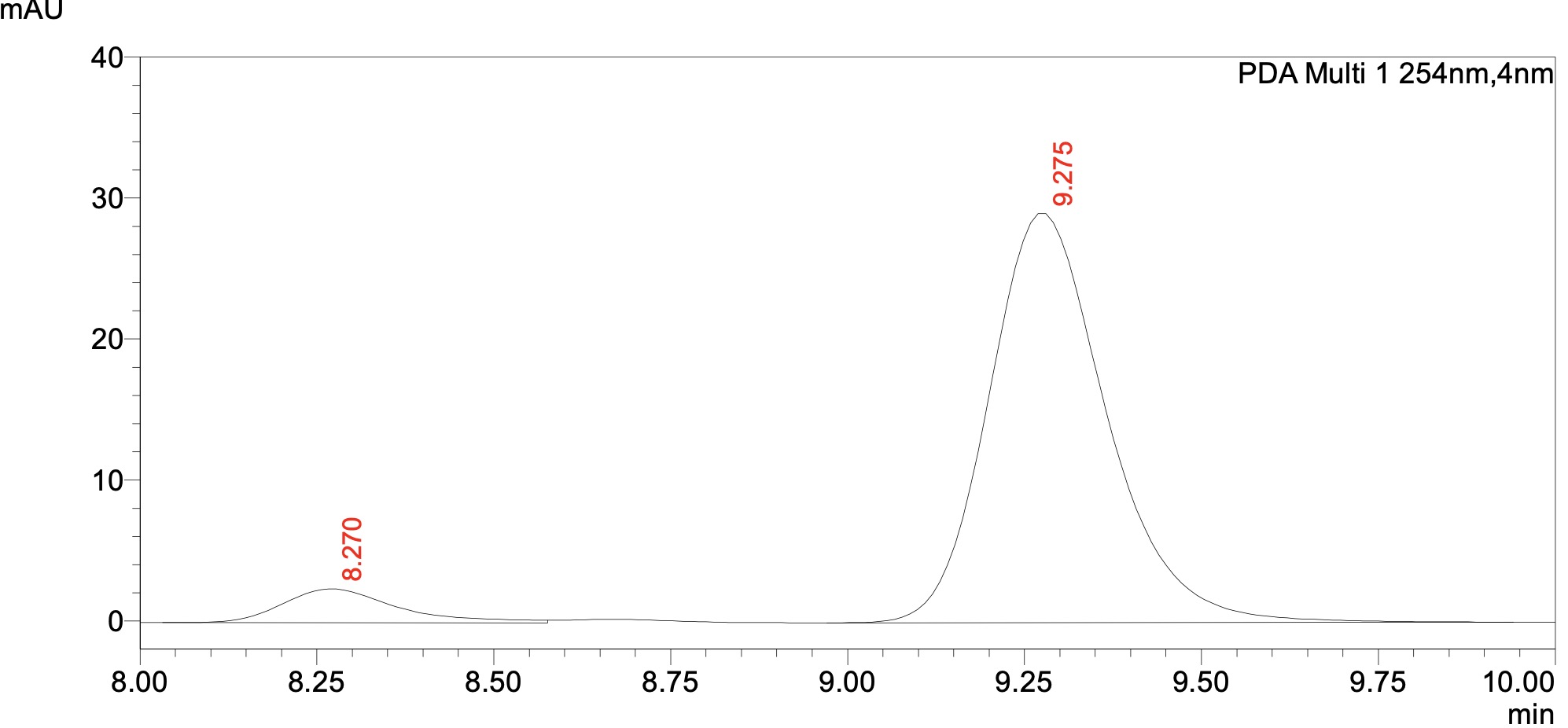


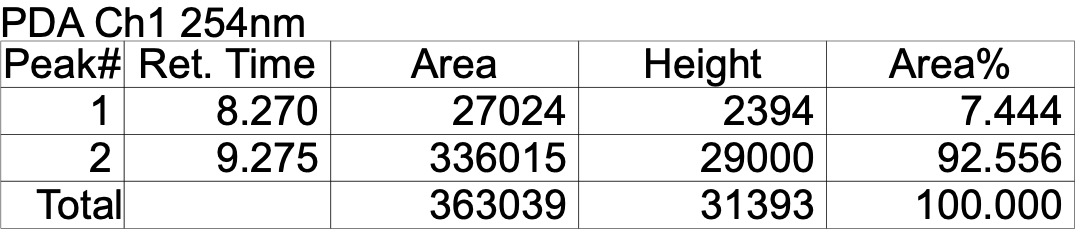


**Racemic 3l**


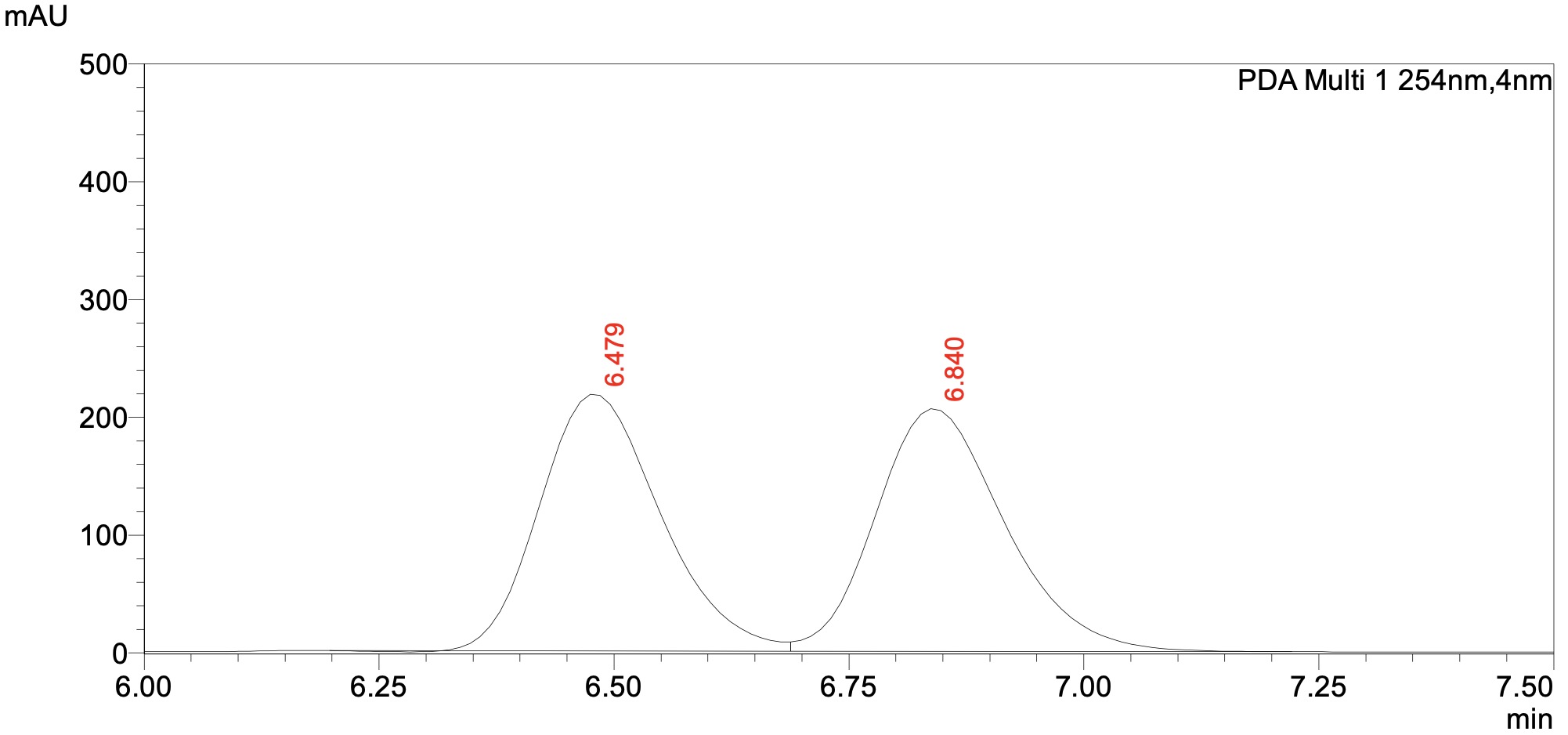


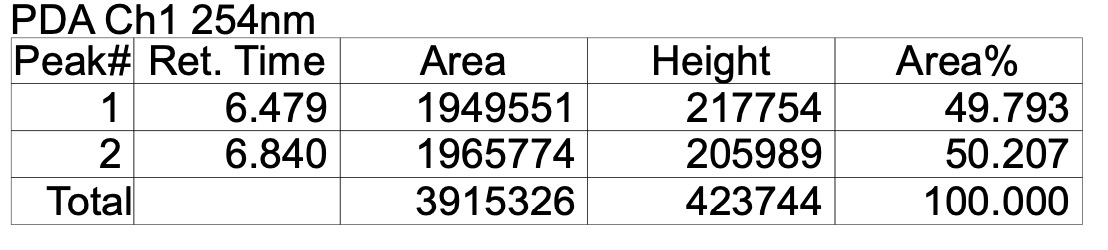


**(R)-3l**


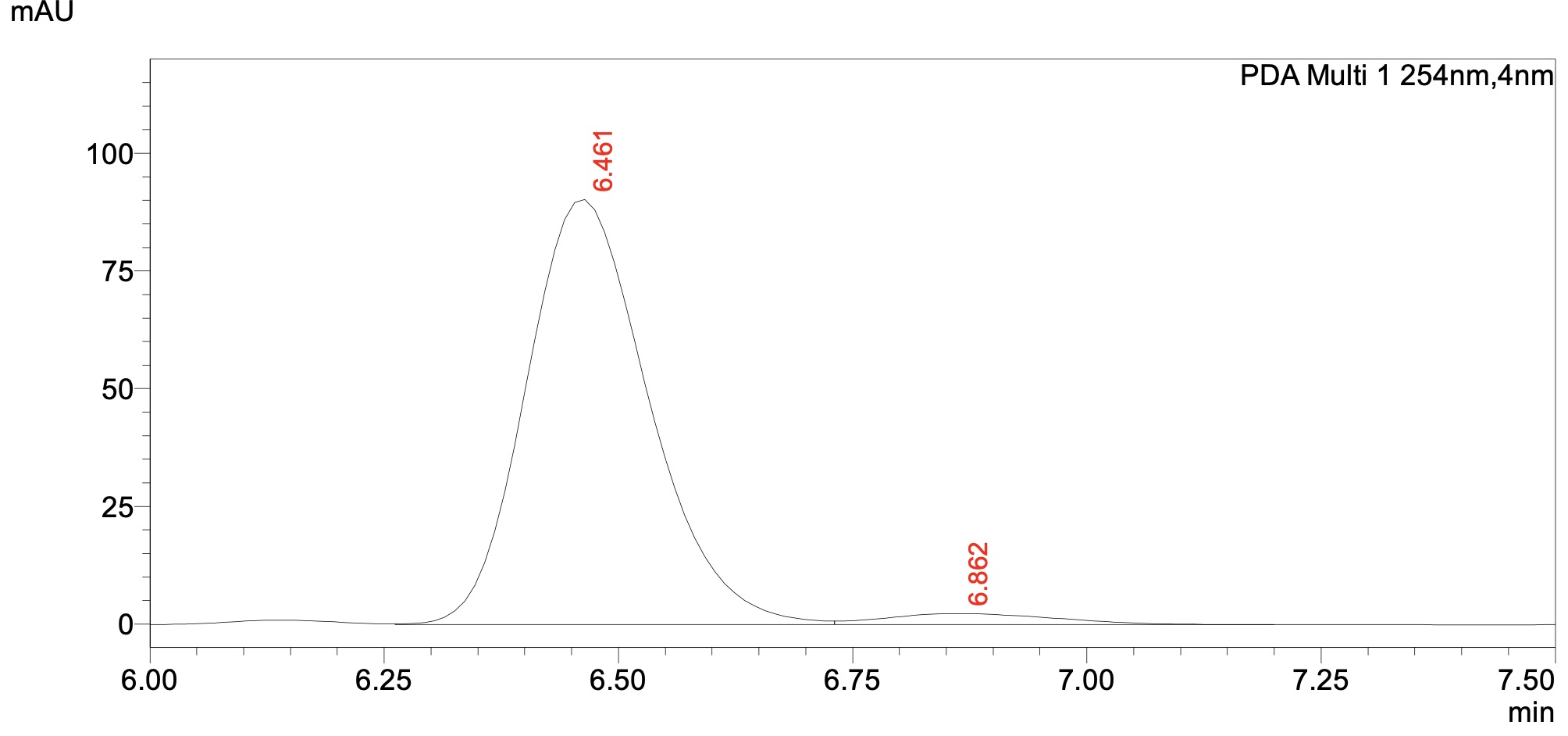


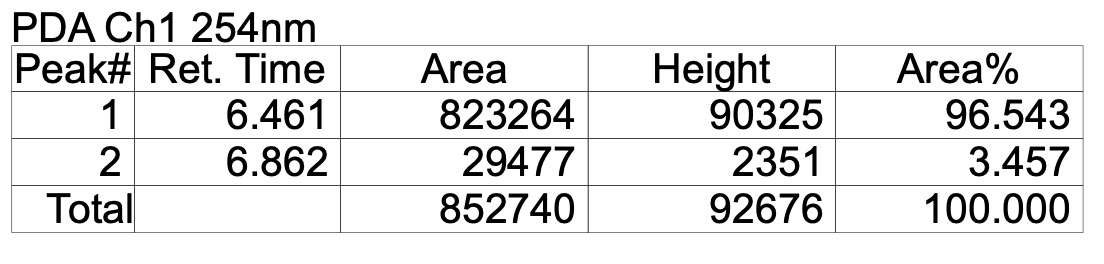


**Racemic 3m**


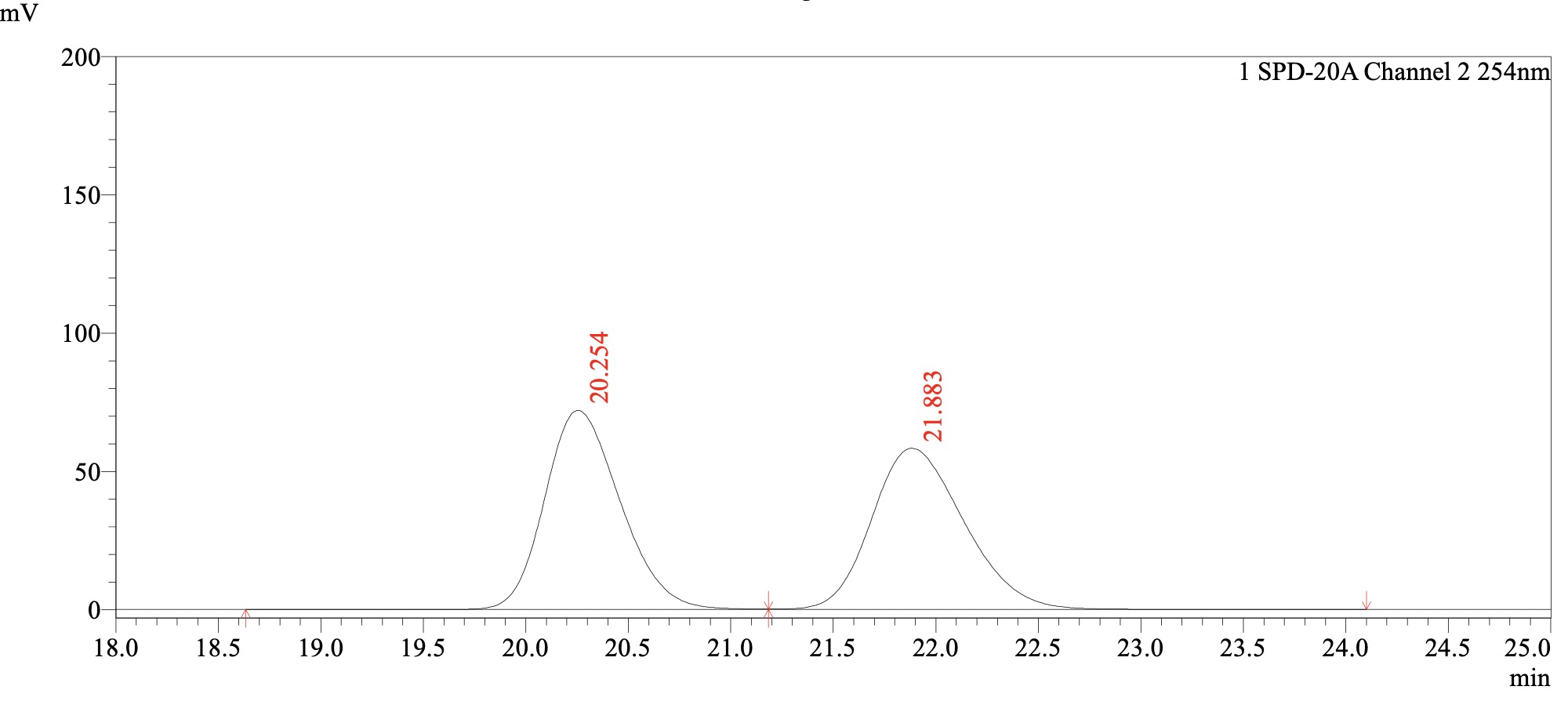


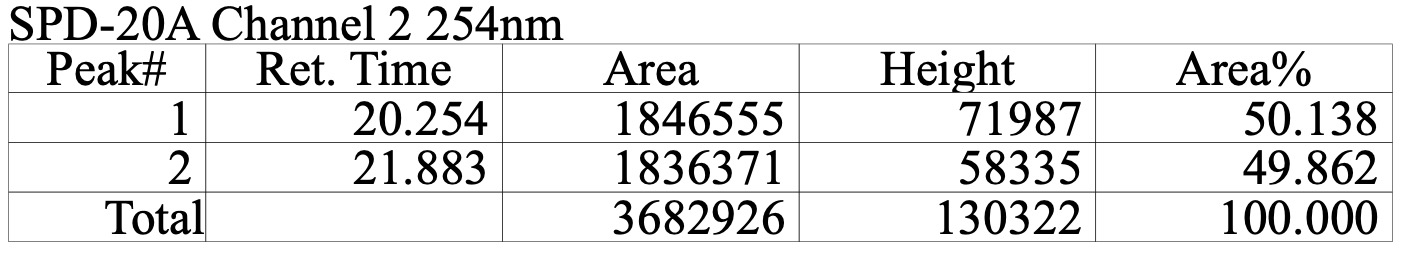


**(R)-3m**


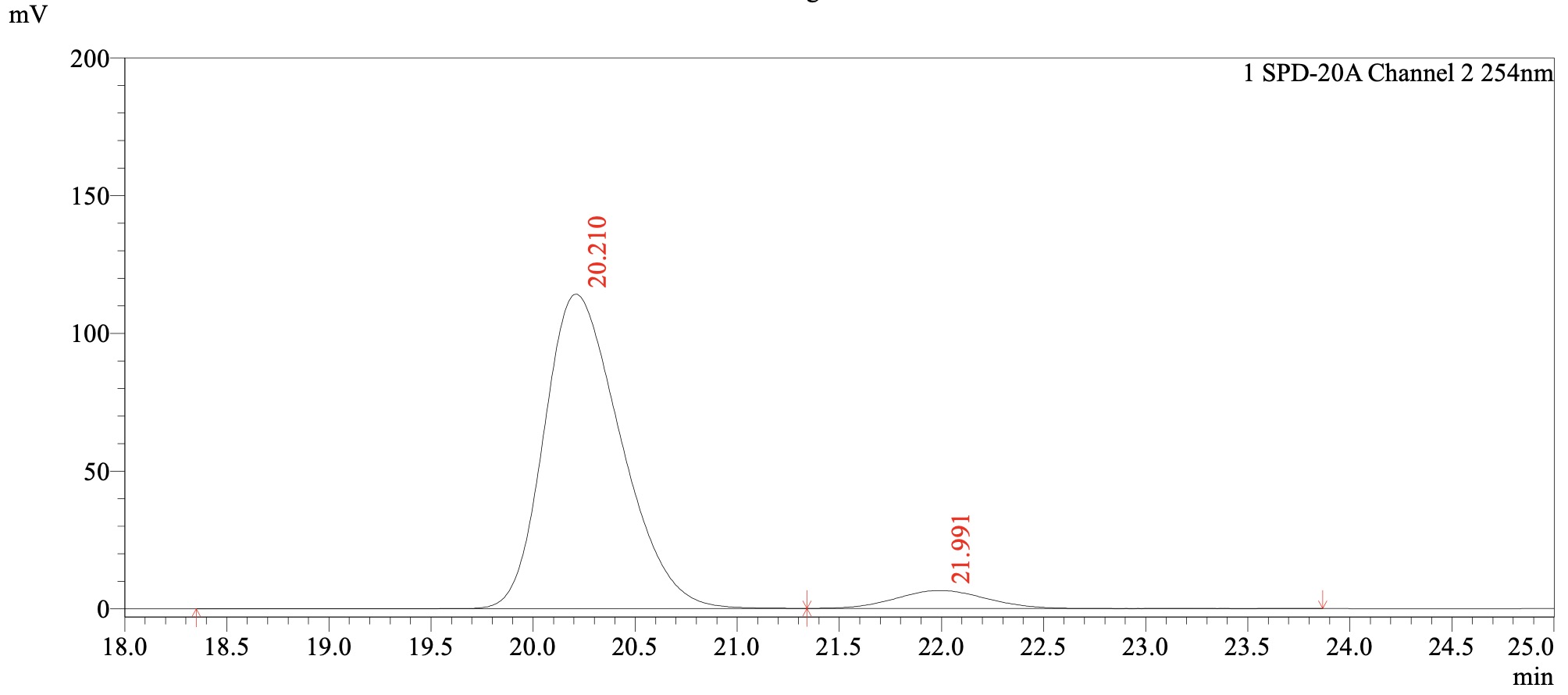


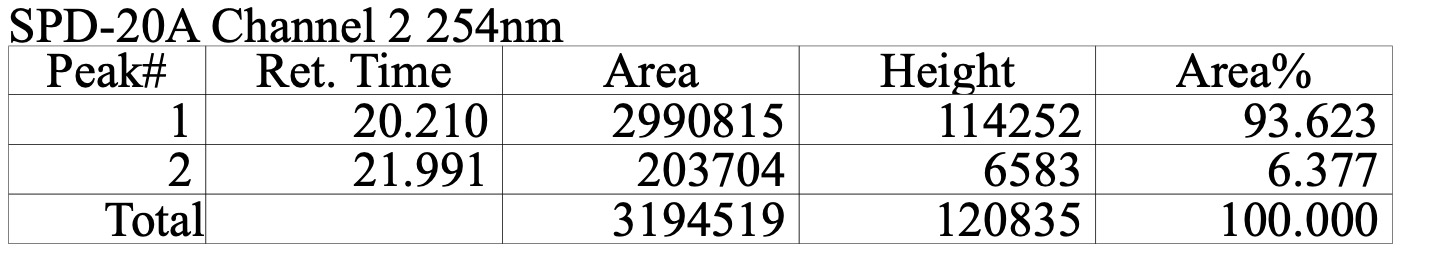


**Racemic 3n**


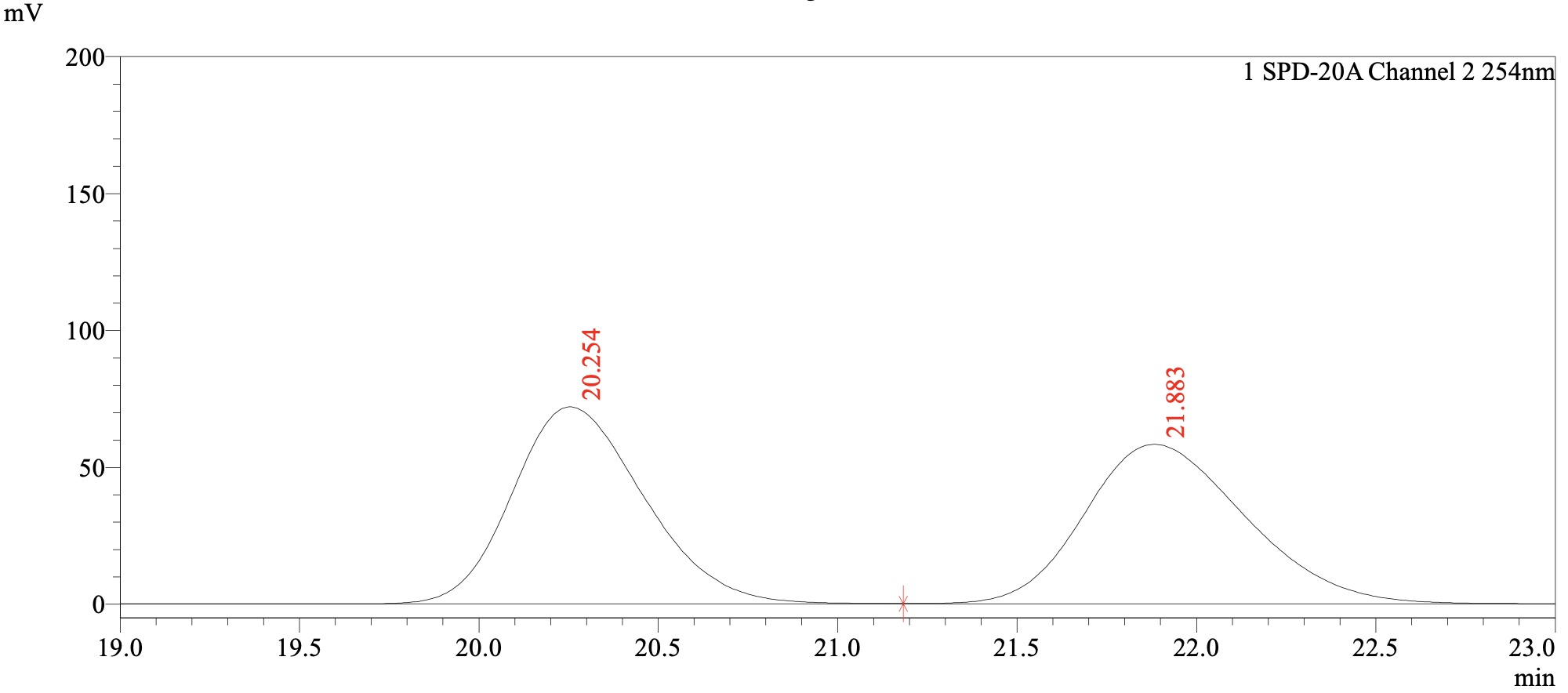


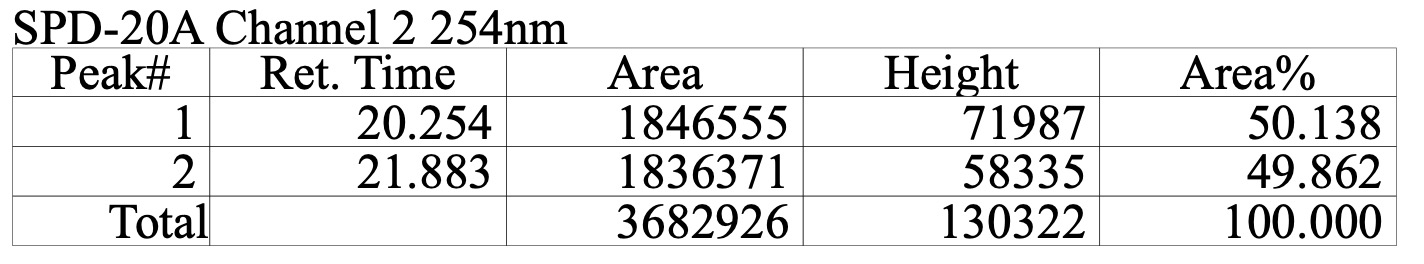


**(R)-3n**


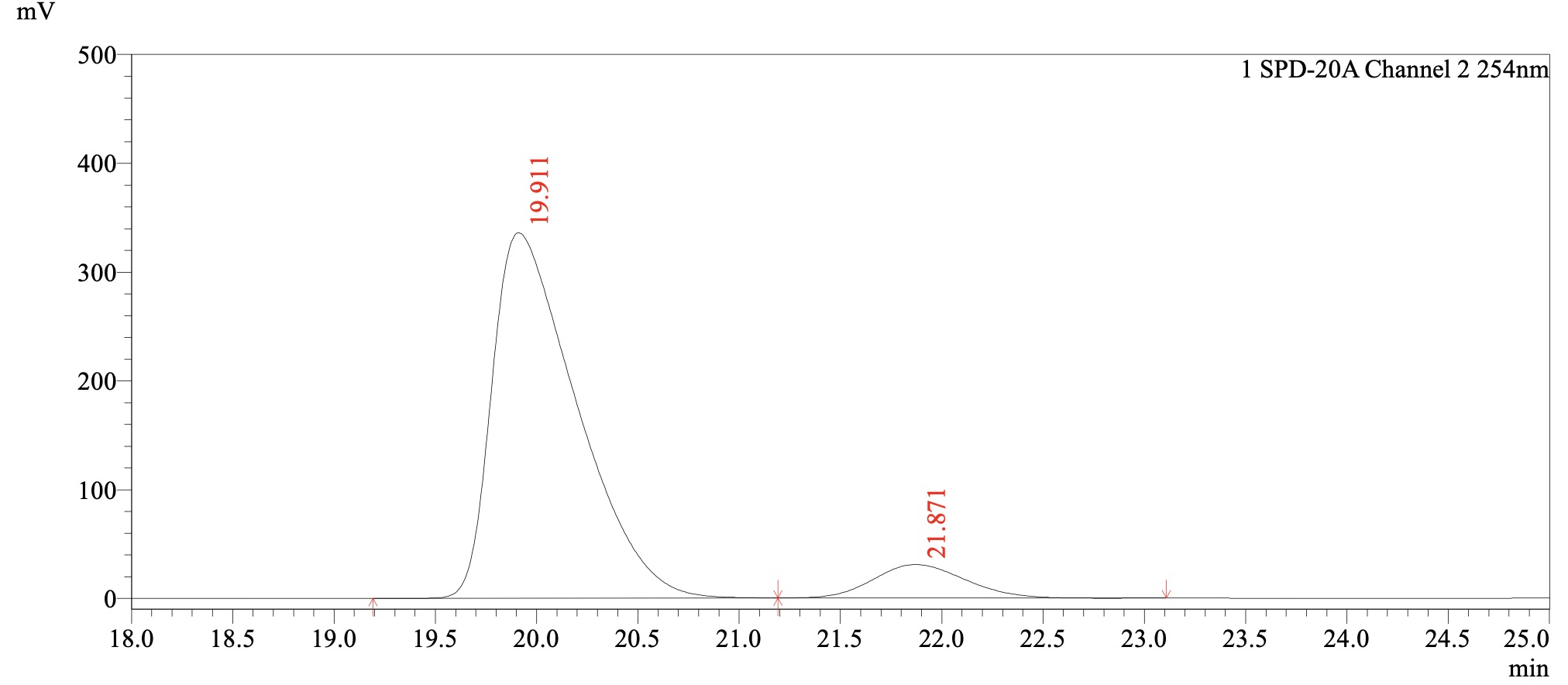


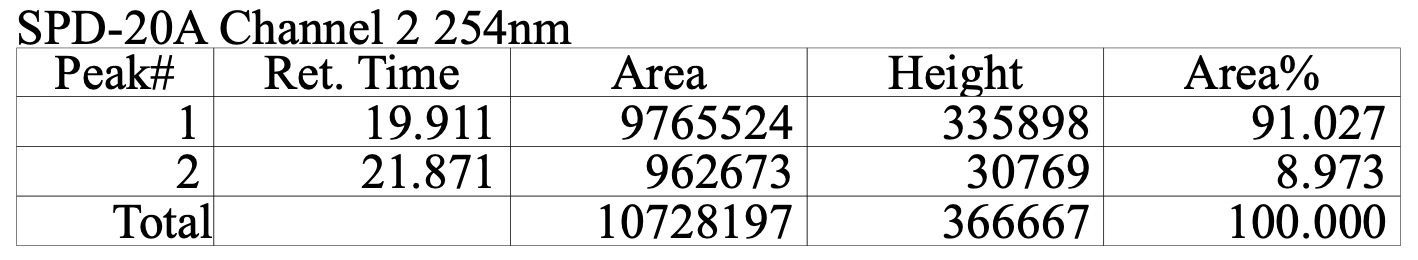


**Racemic 3o**


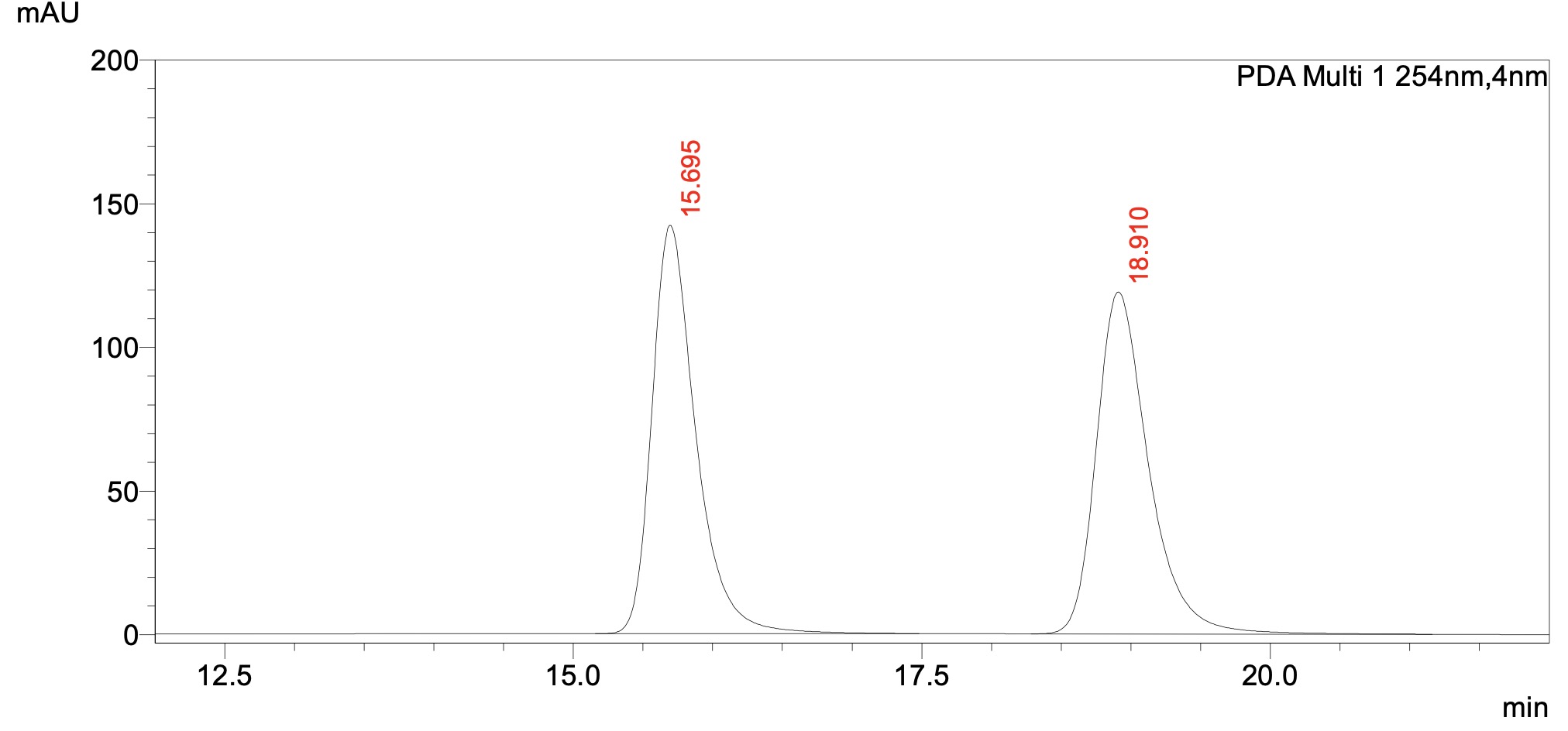


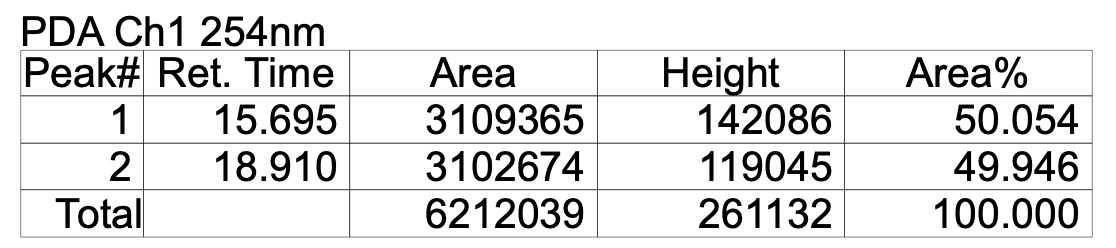


**(R)-3o**


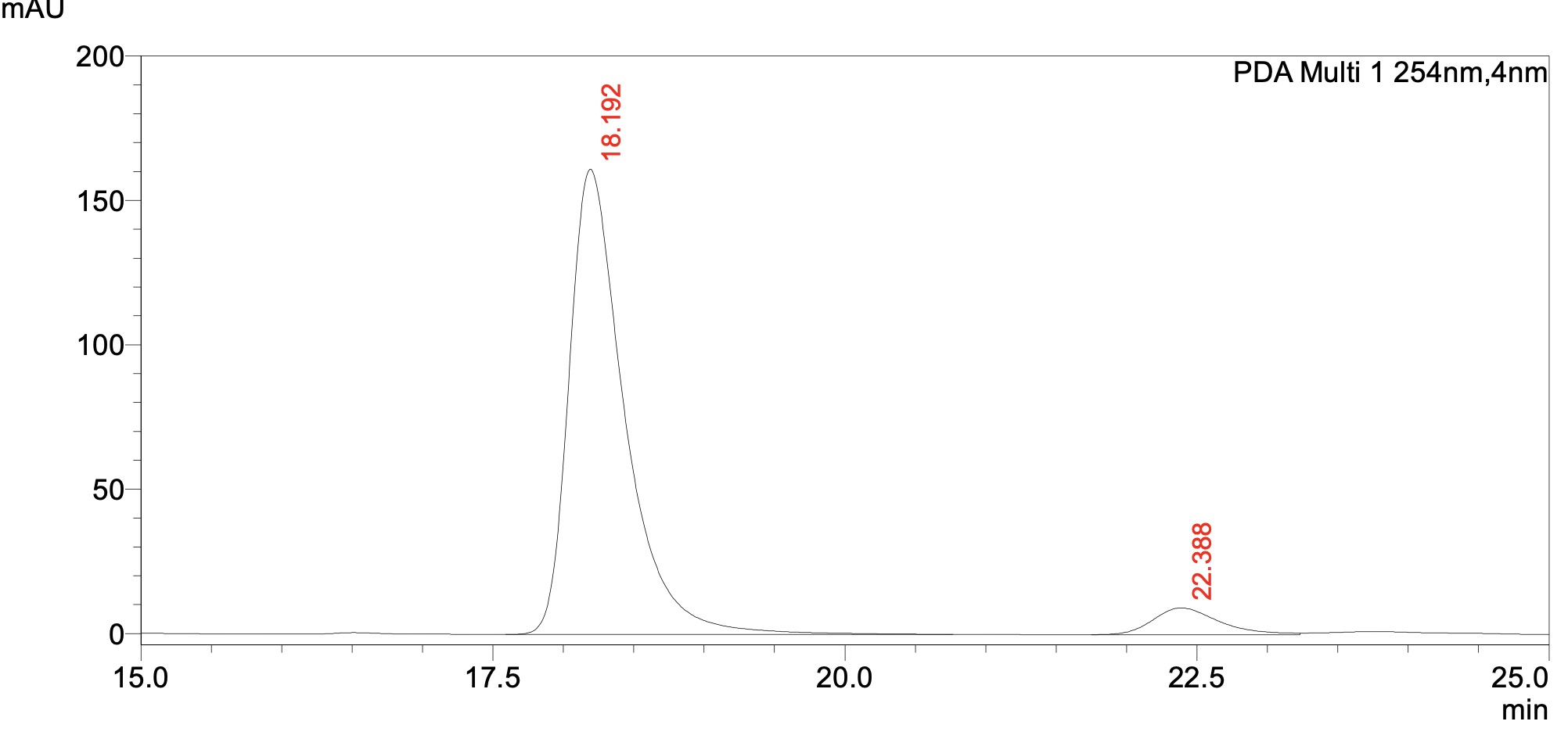


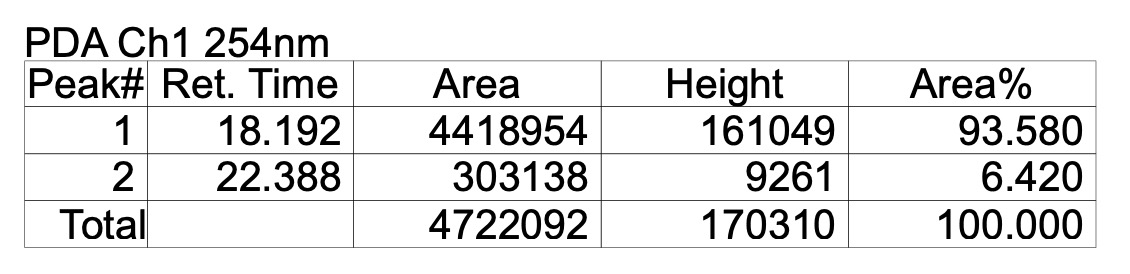


**Racemic 3p**


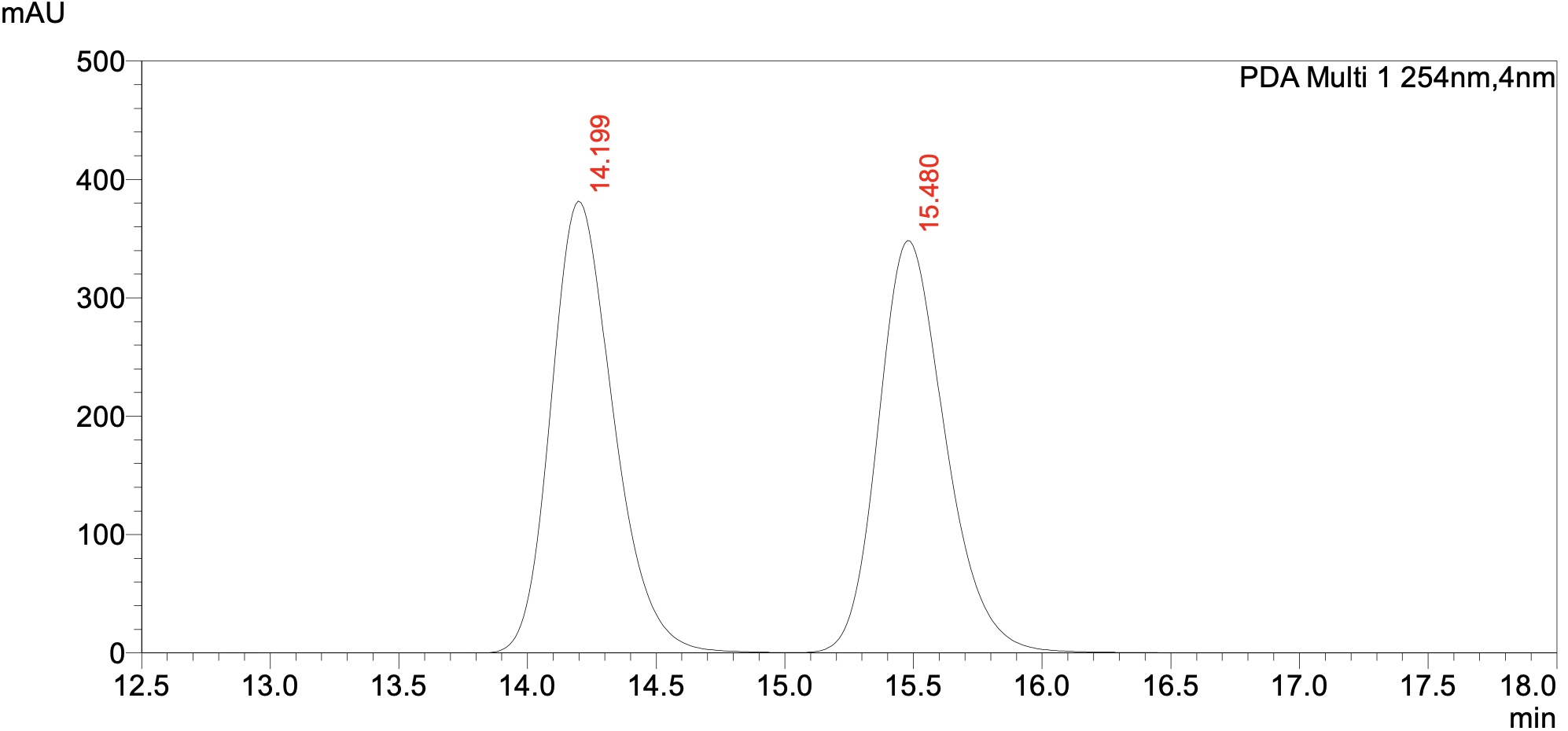


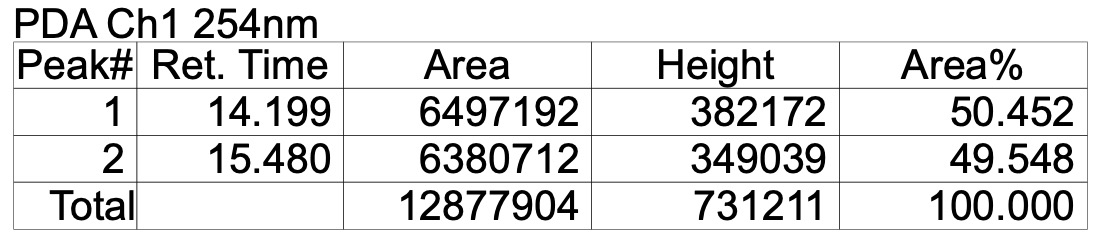


**(R)-3p**


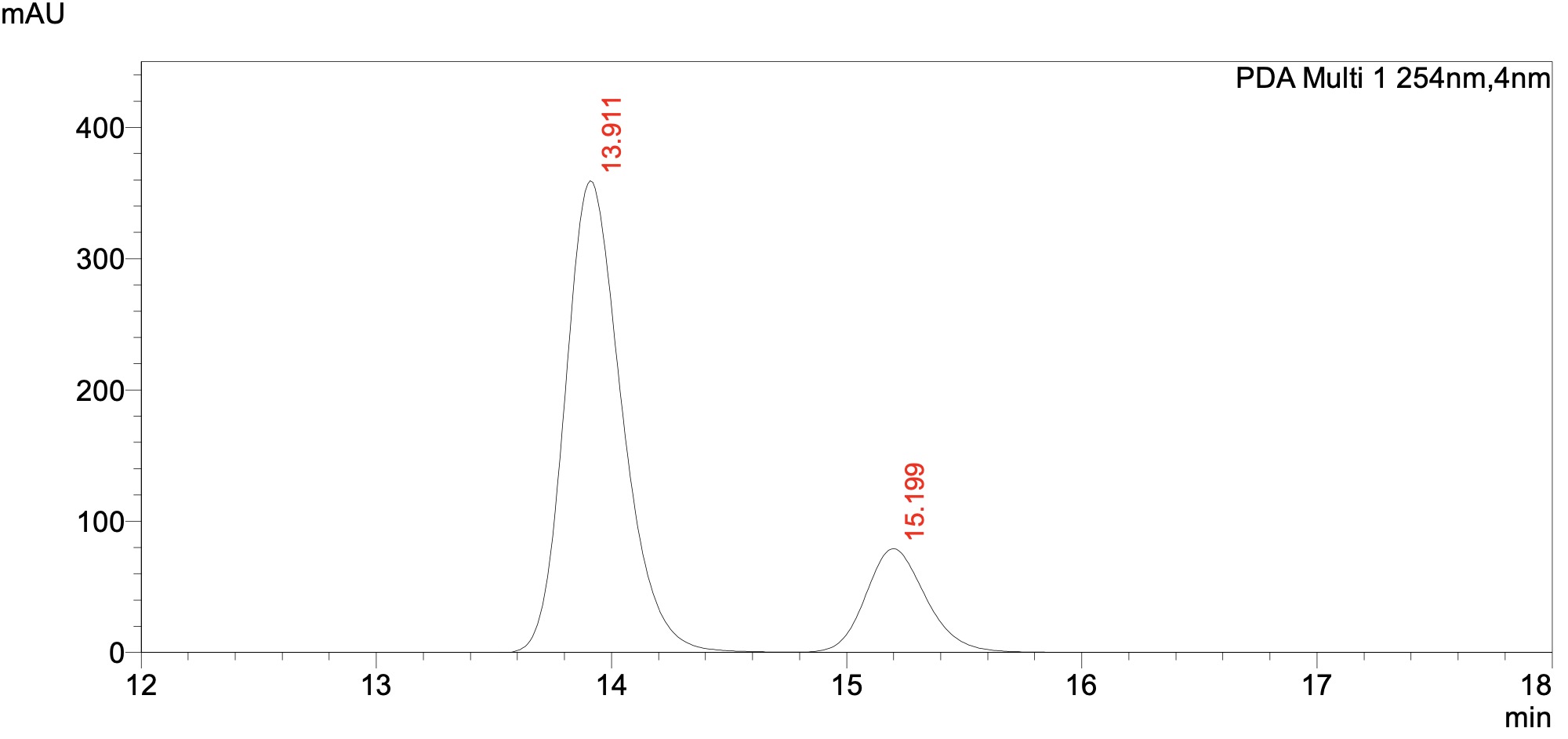


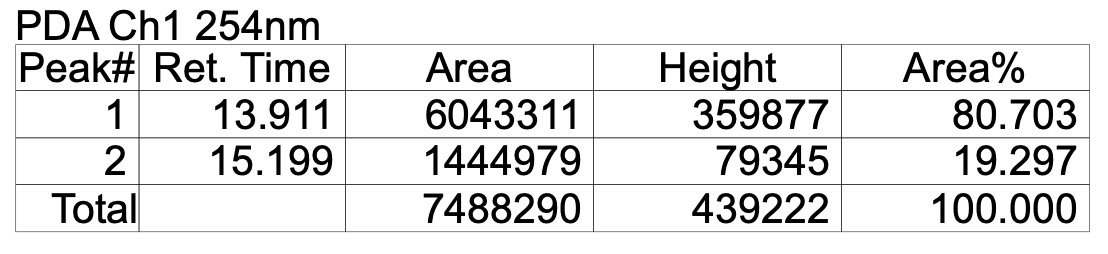


**Racemic 3q**


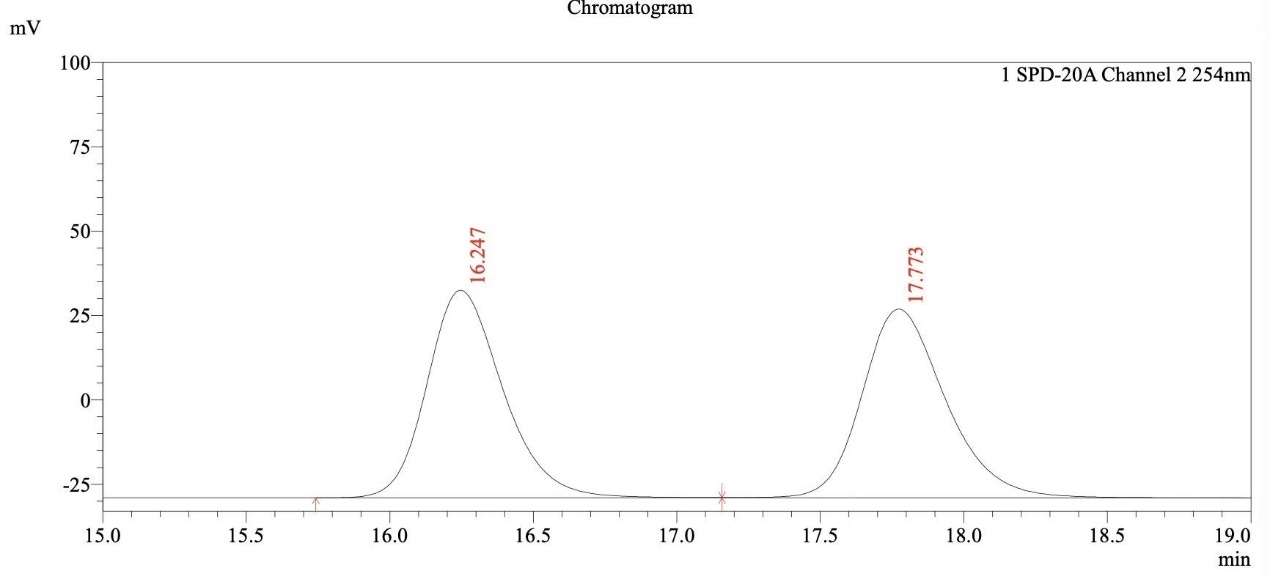


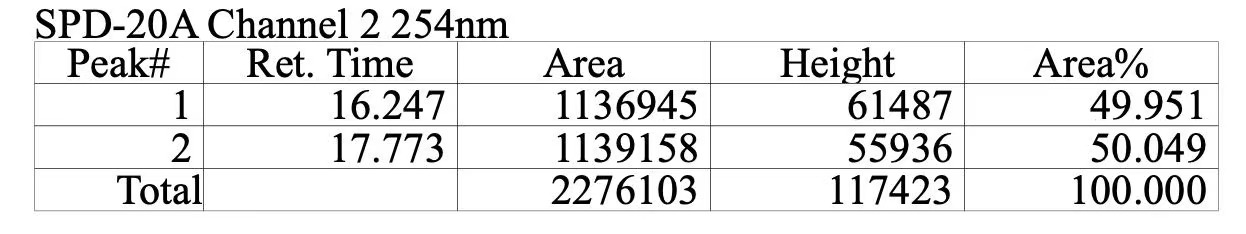


**(R)-3q**

**
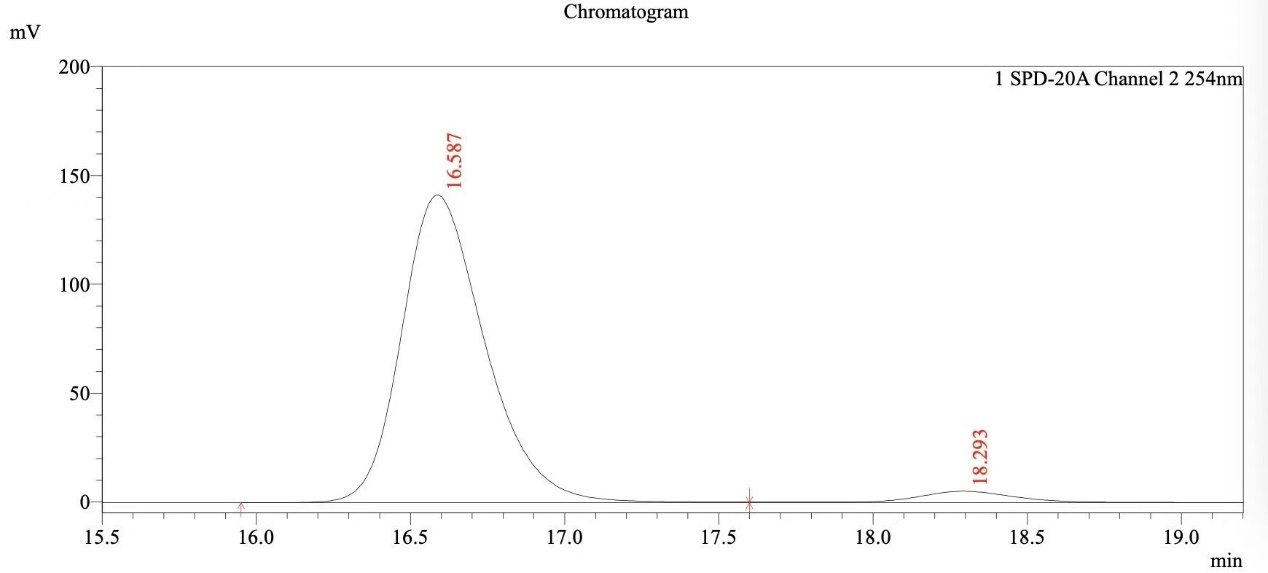
**

**
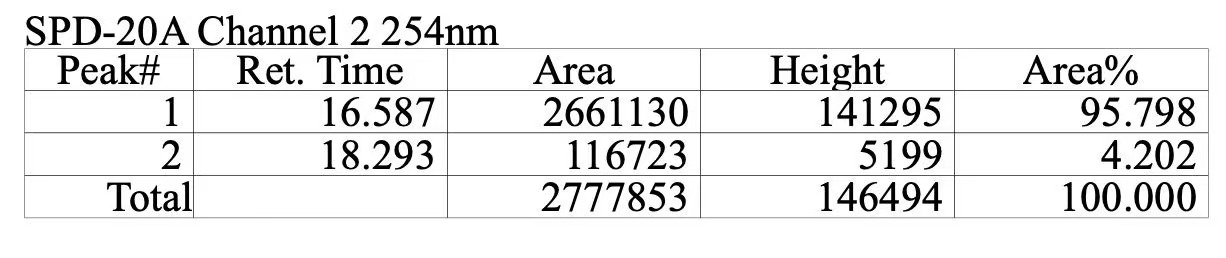
**

**Racemic 3r**


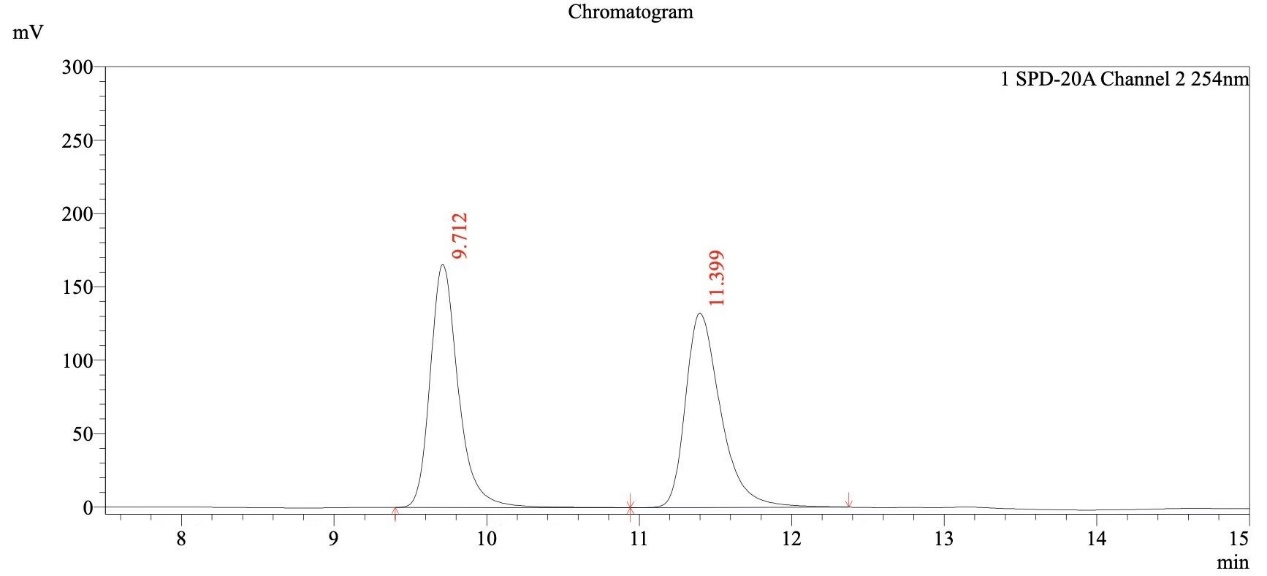


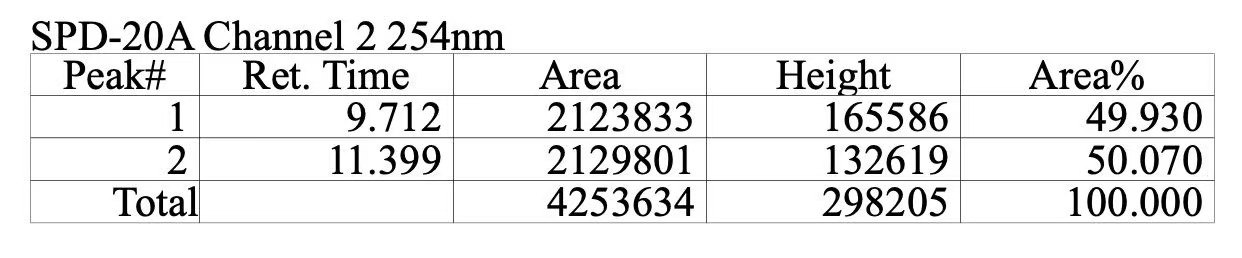


**(R)-3r**

**
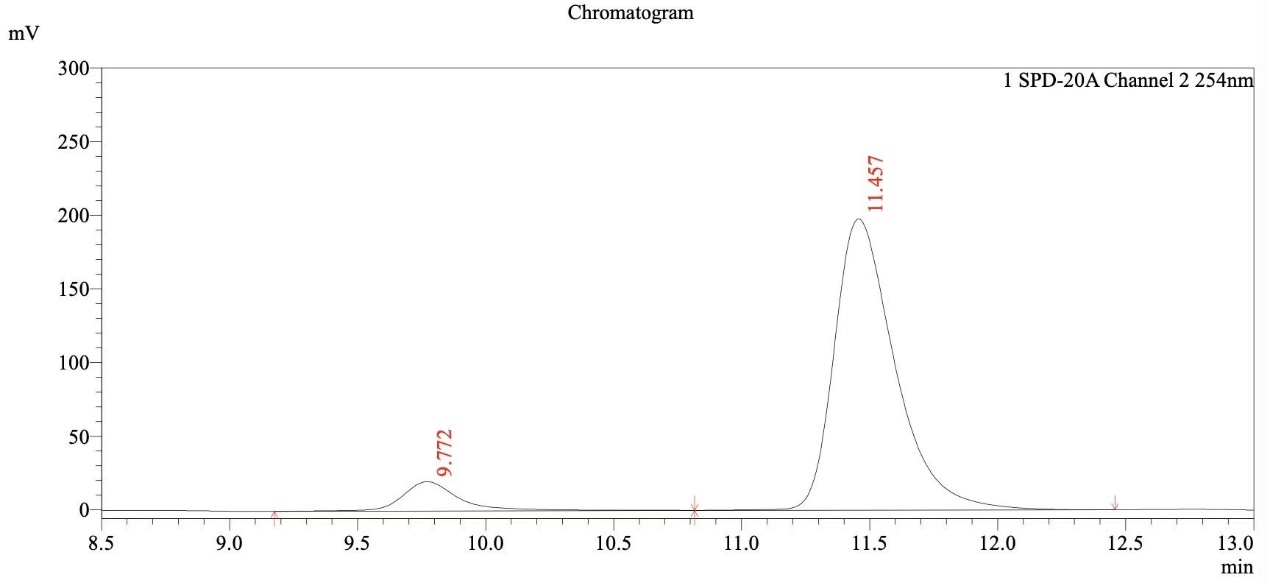
**

**
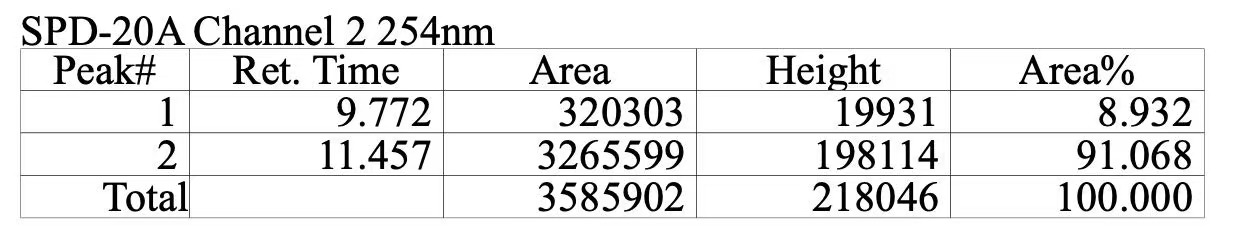
**

**Racemic 3s**


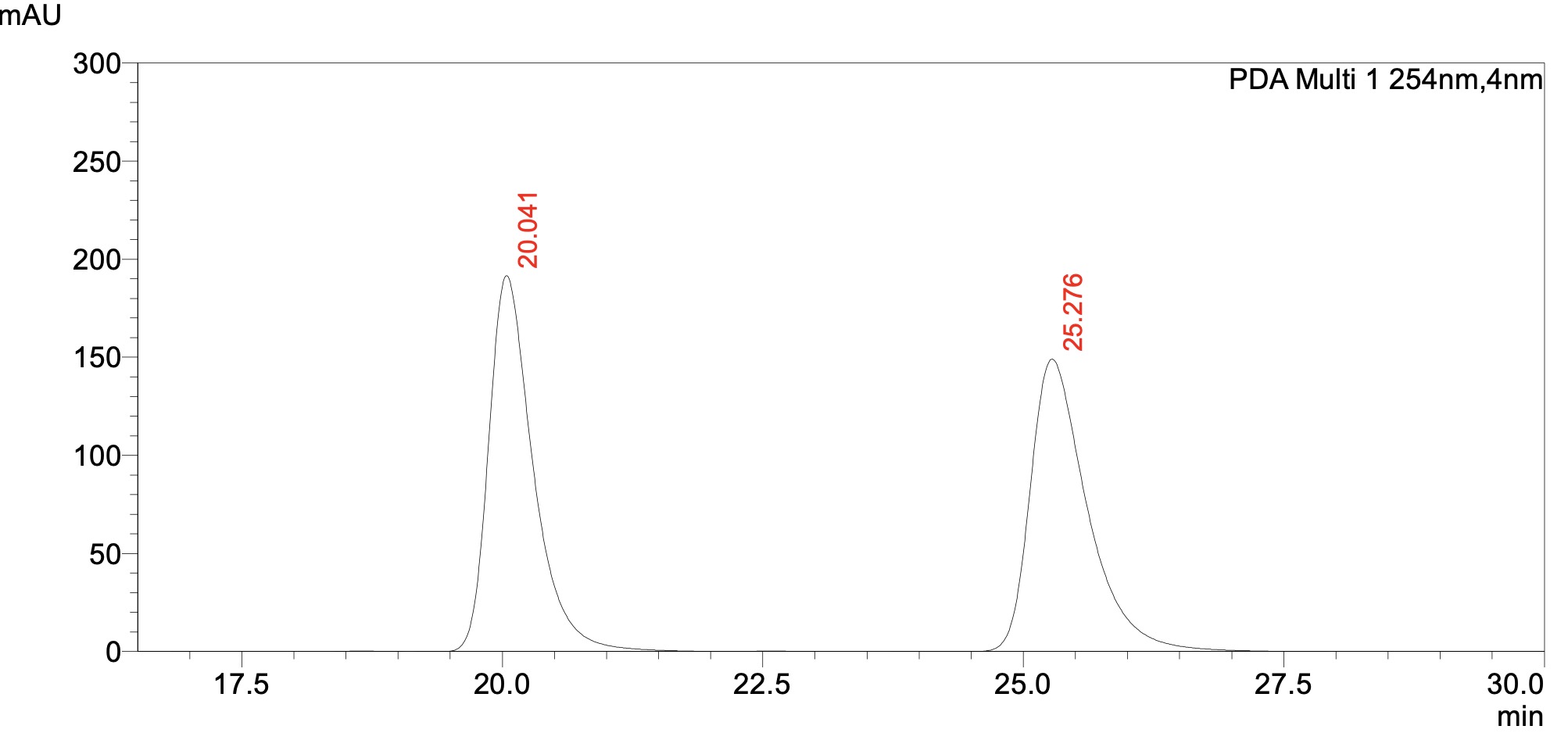


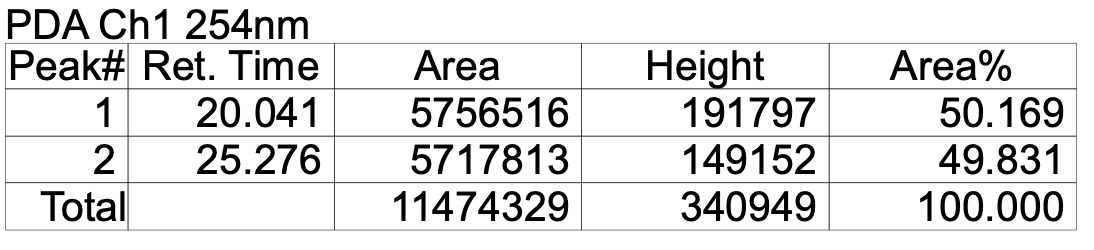


**(R)-3s**


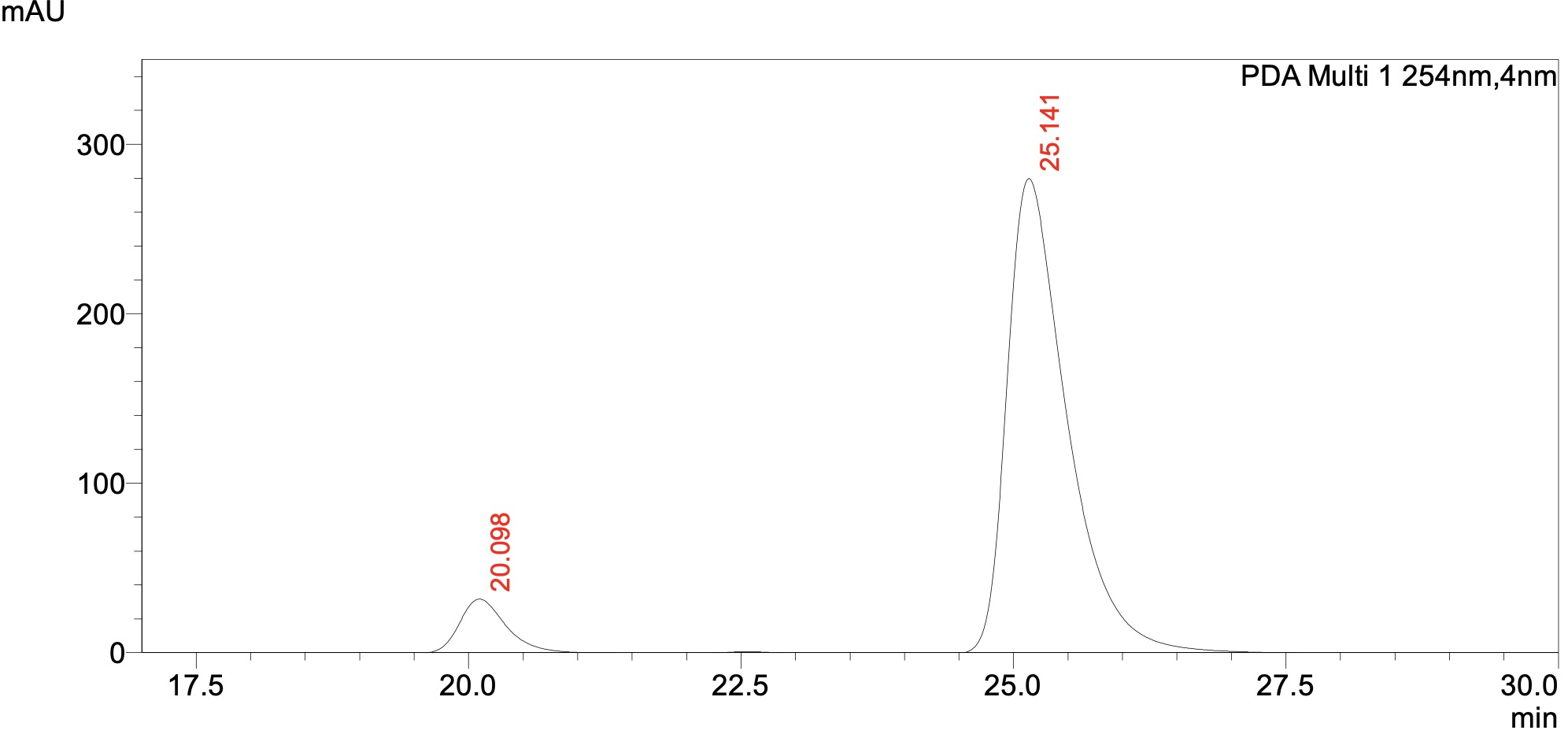


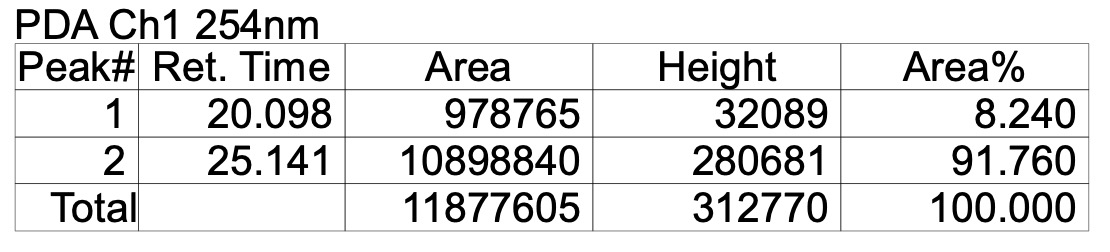


**Racemic 3t**


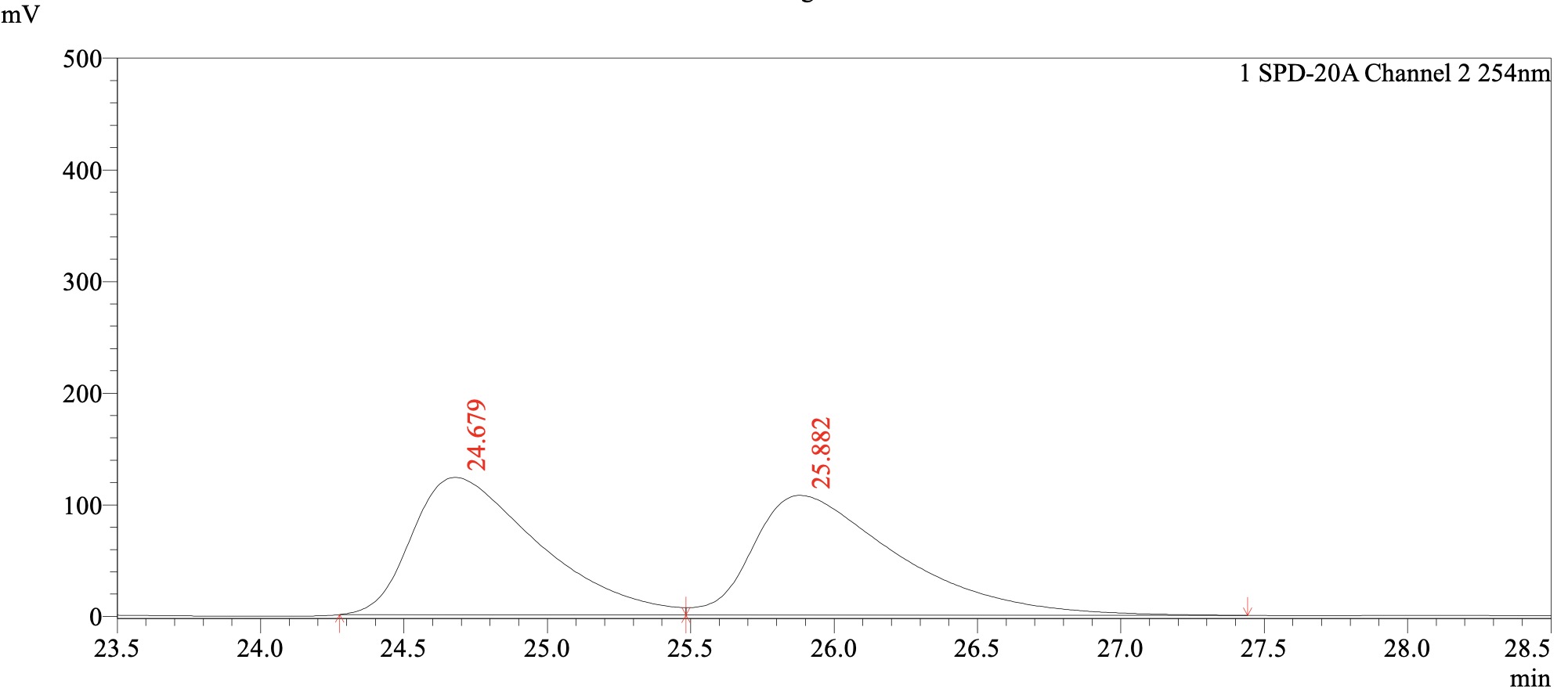


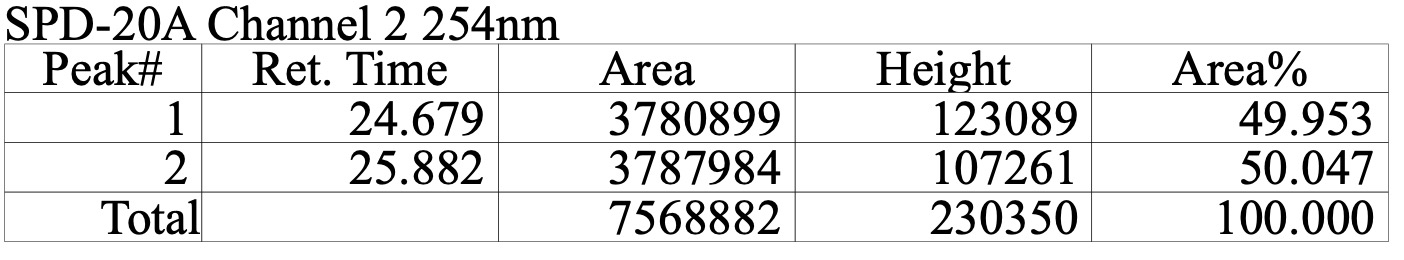


**(R)-3t**


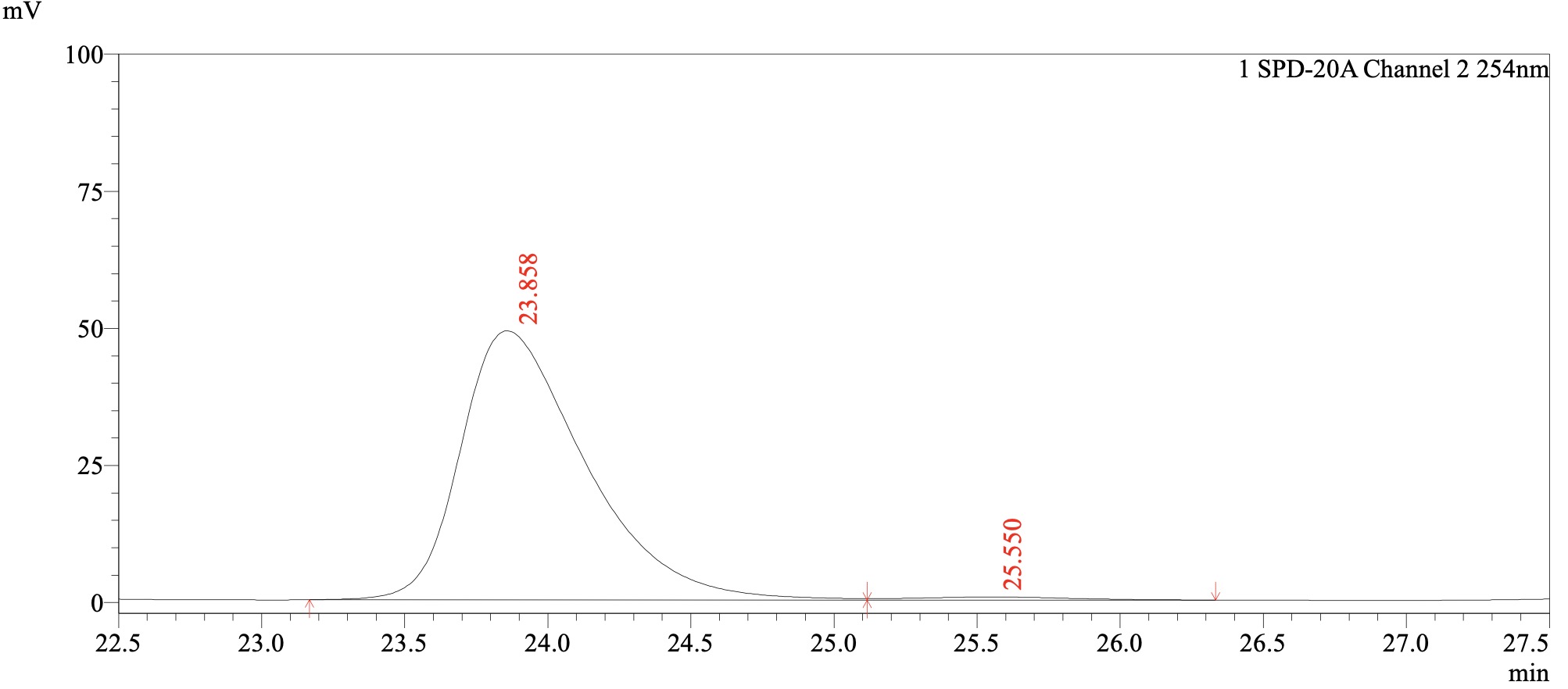

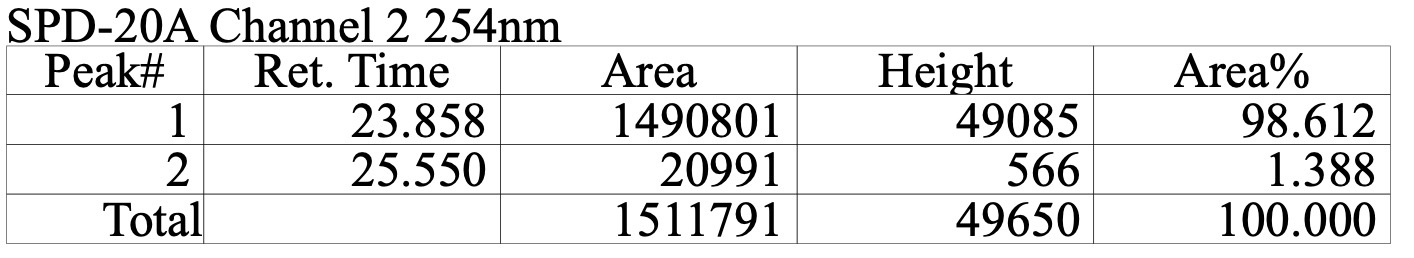


**Racemic 3u**


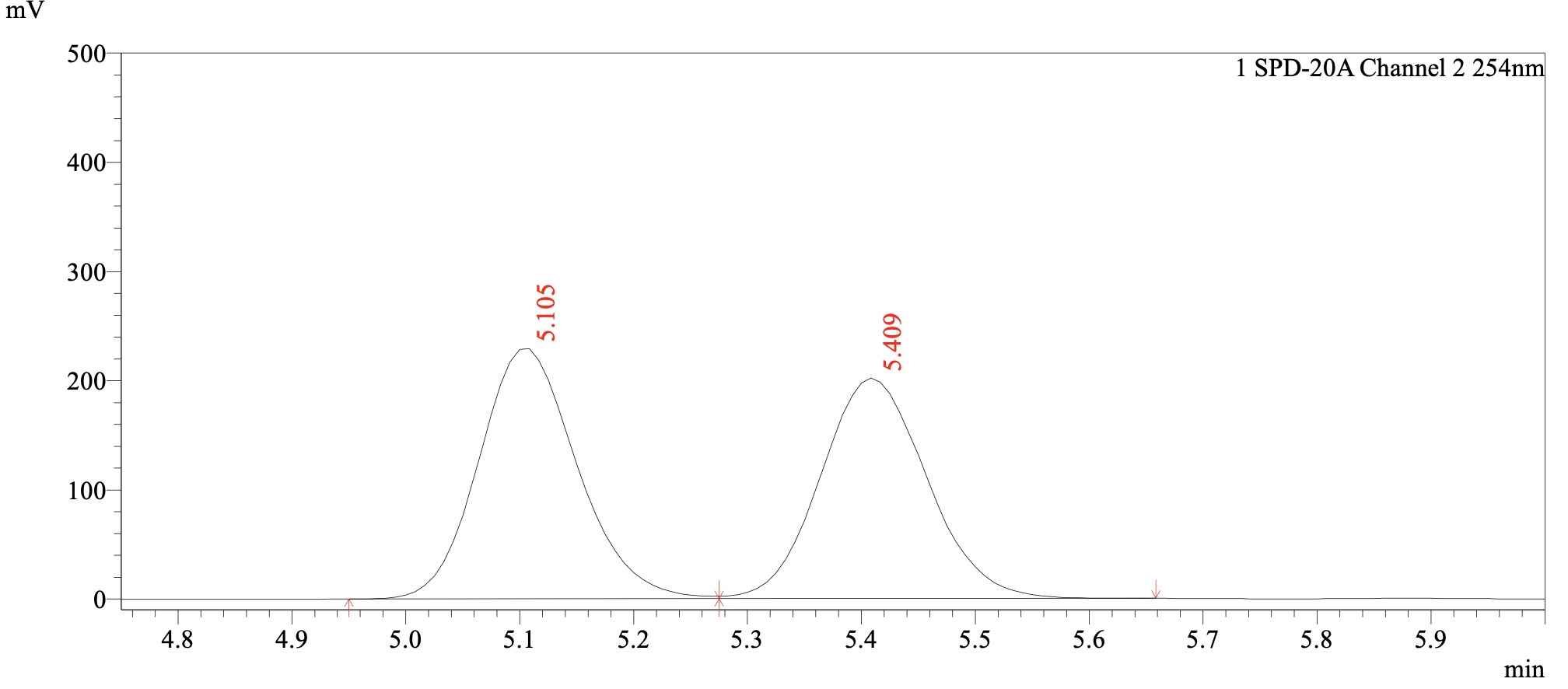


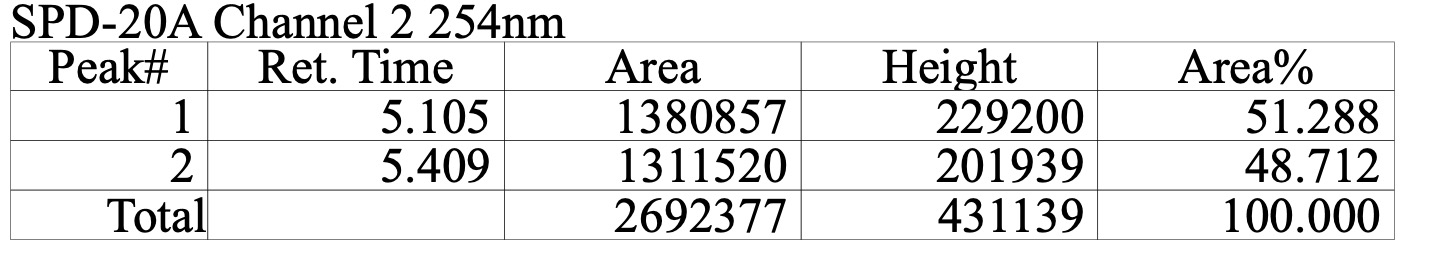


**(R)-3u**


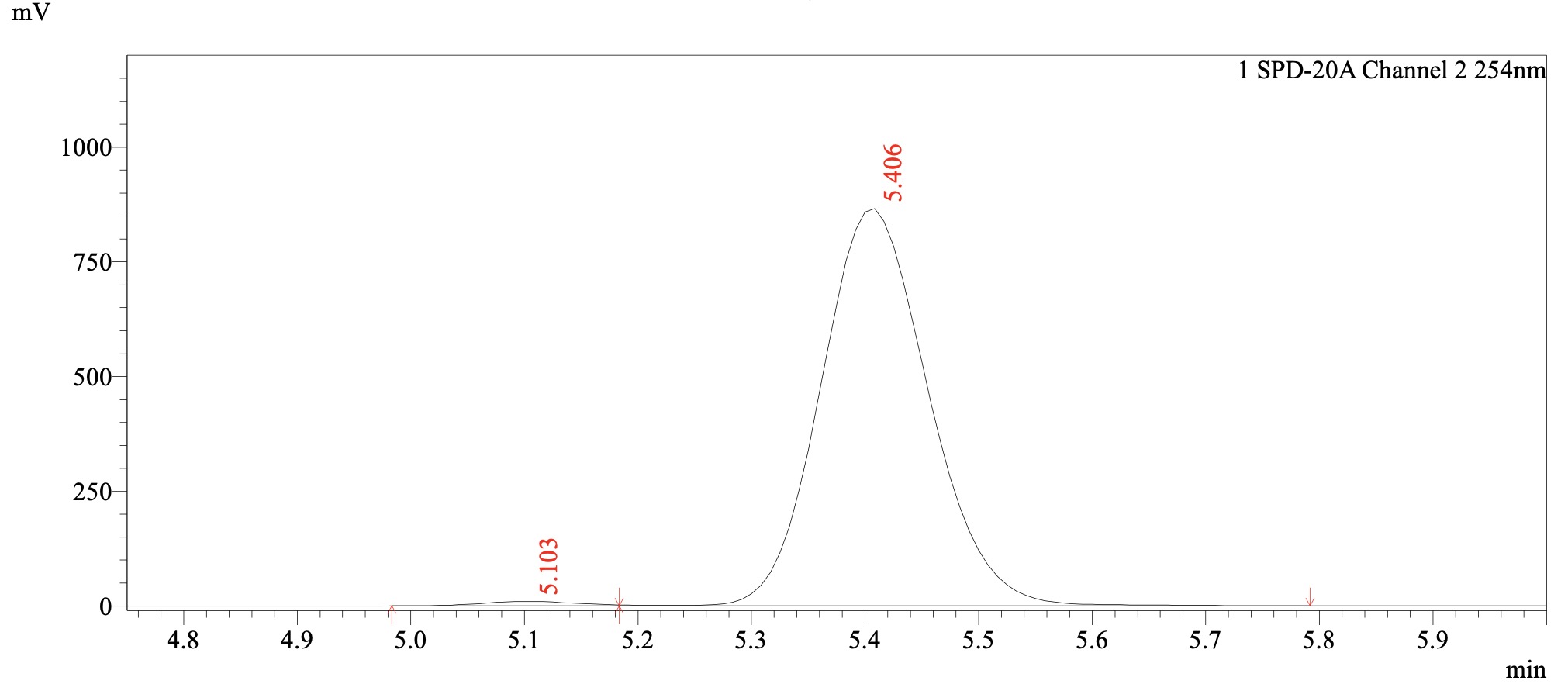


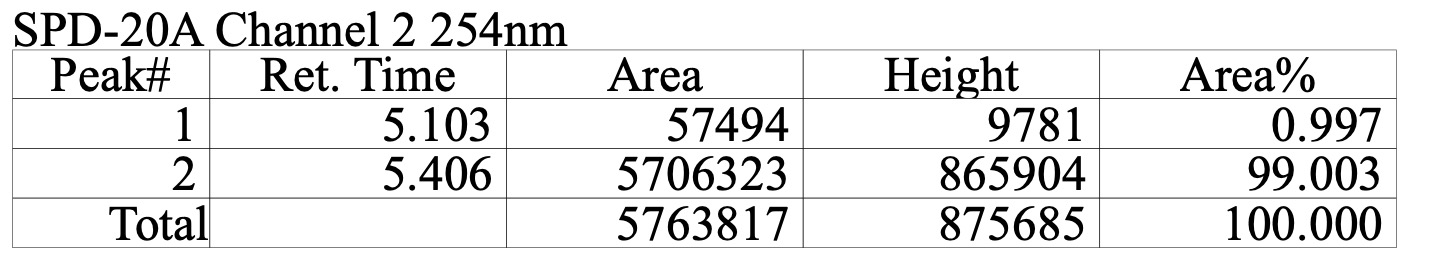


**Racemic 3v**


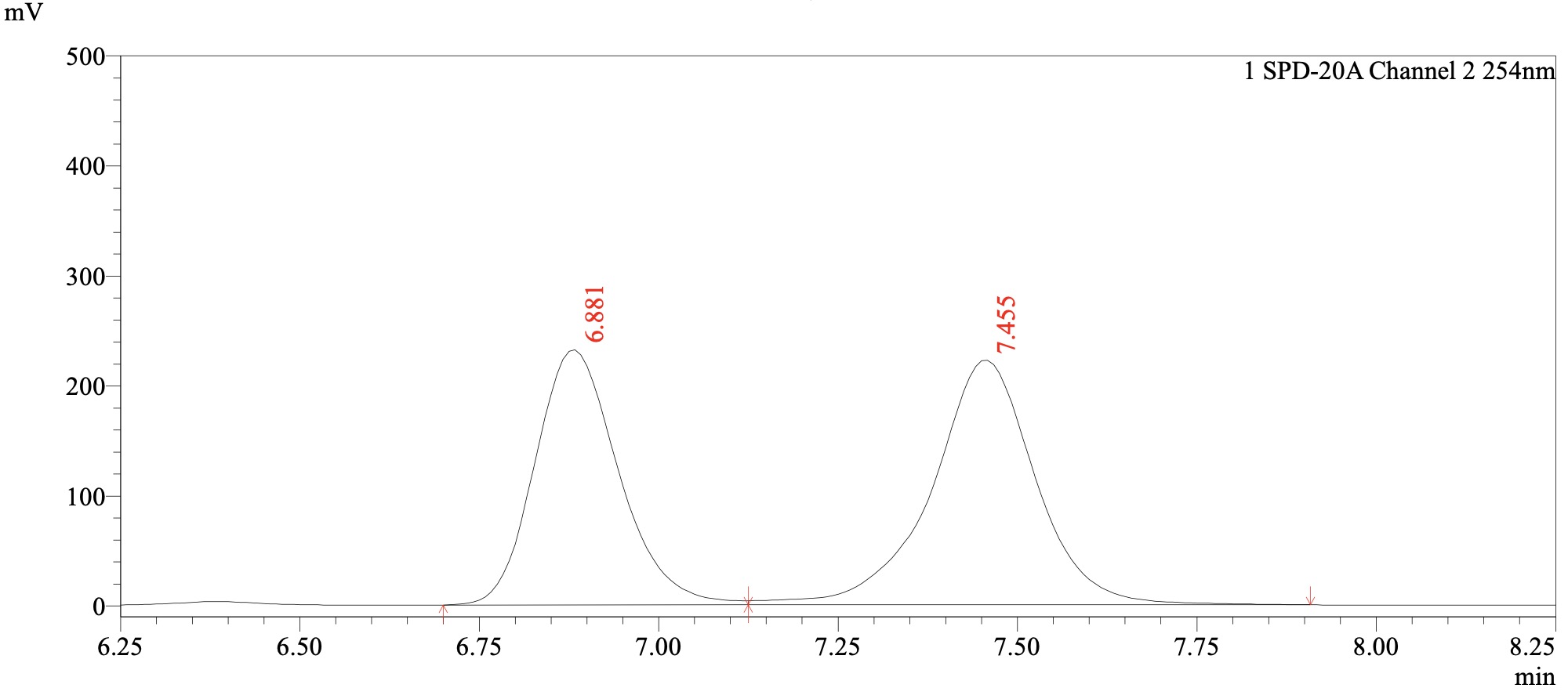


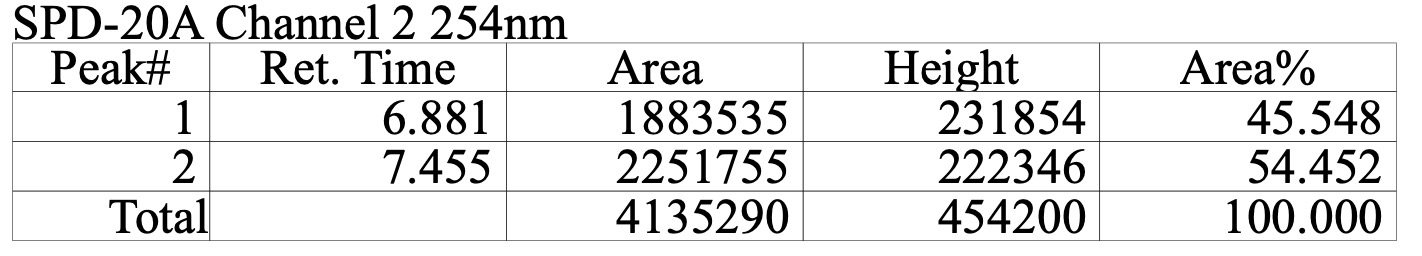


**(R)-3v**


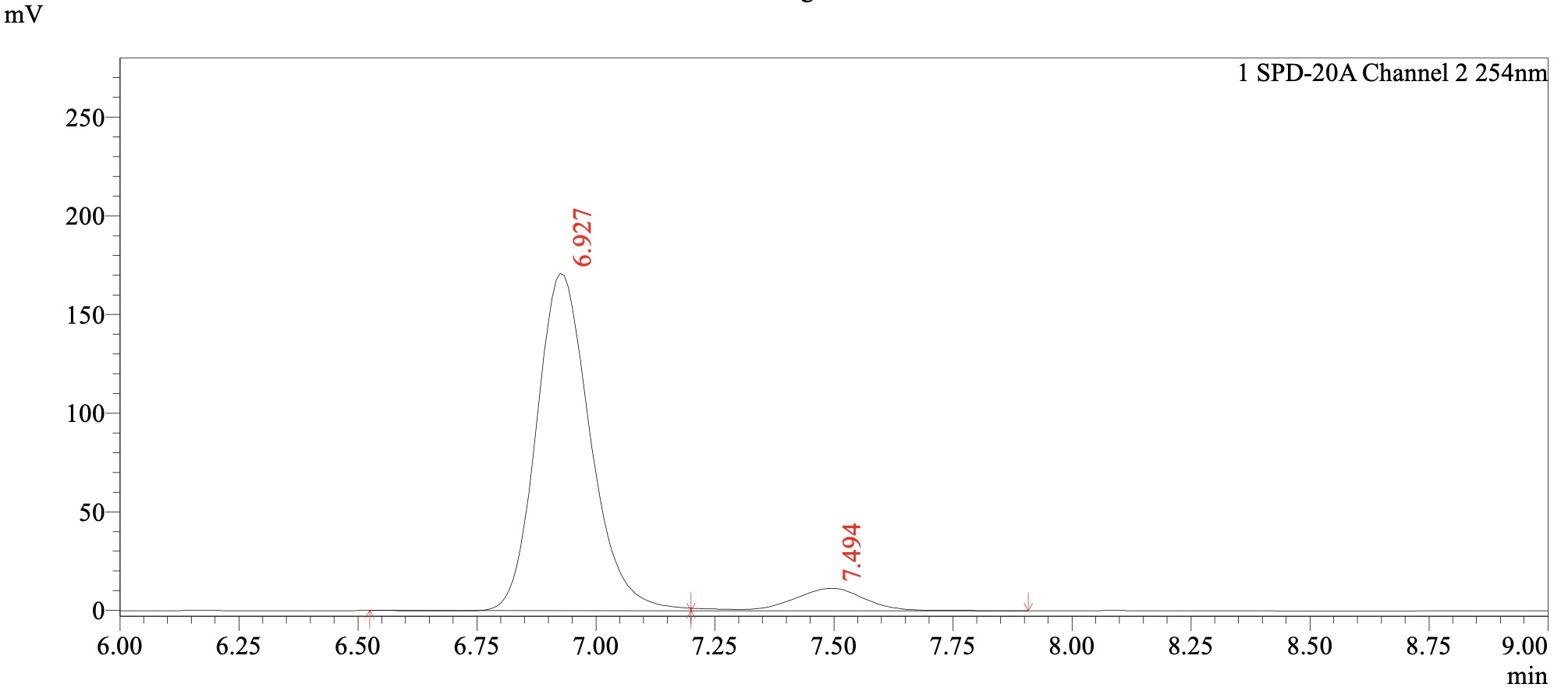


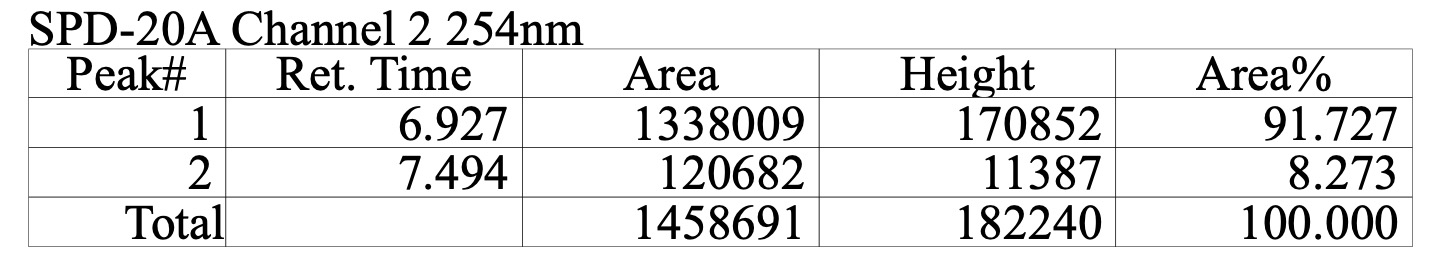


**Racemic 3w**


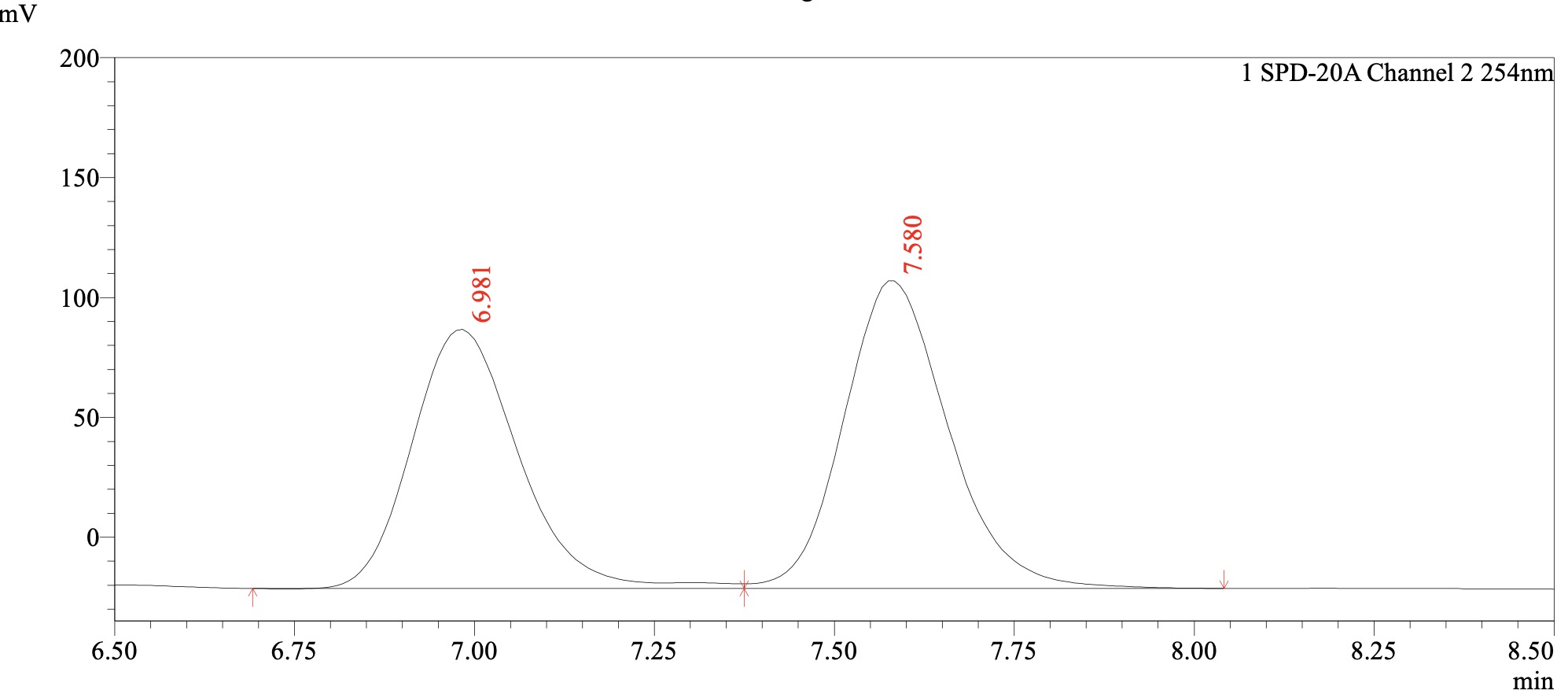


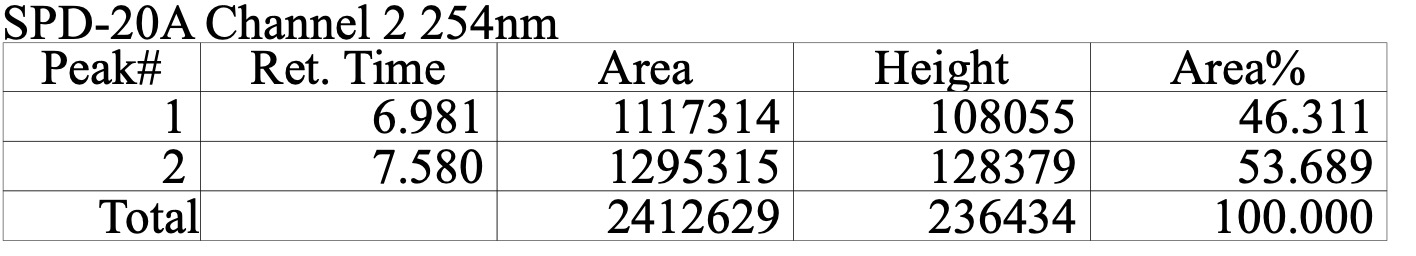


**(R)-3w**


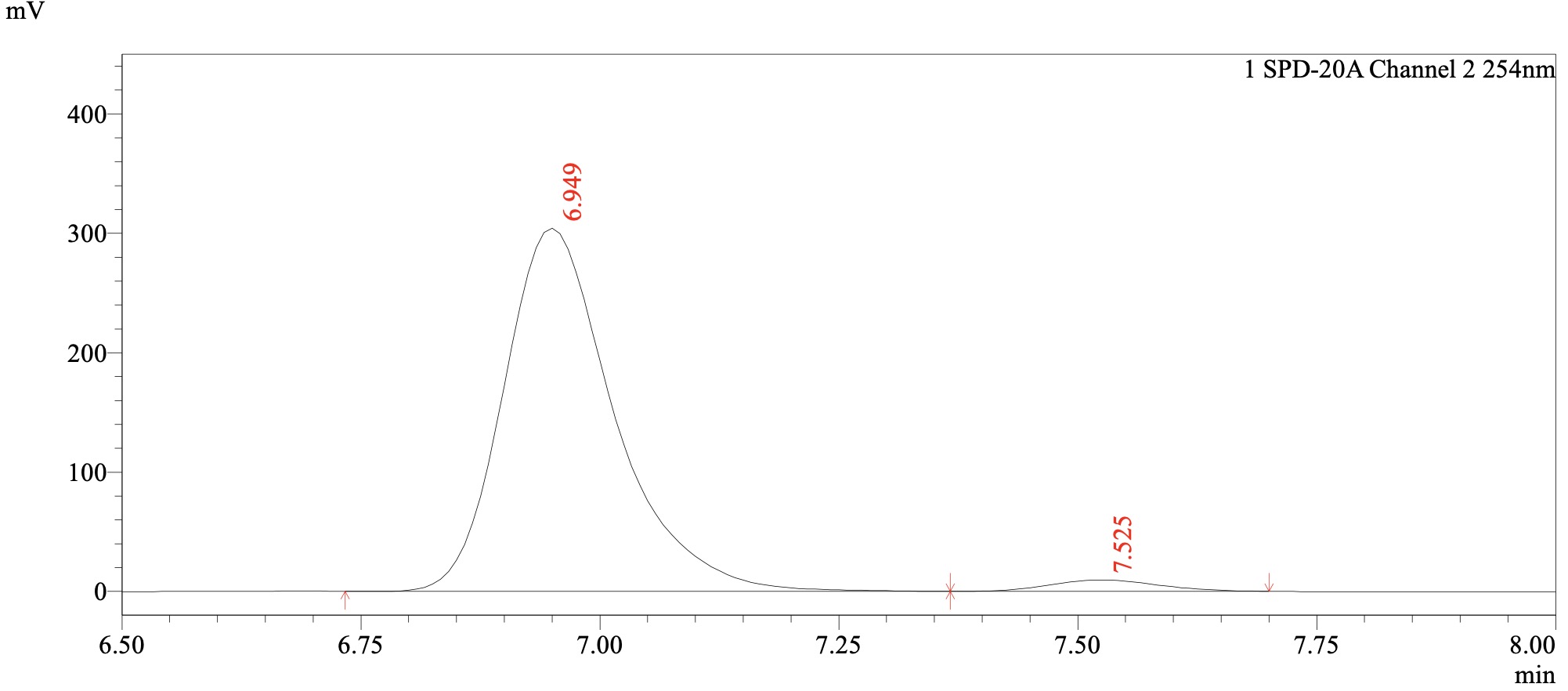


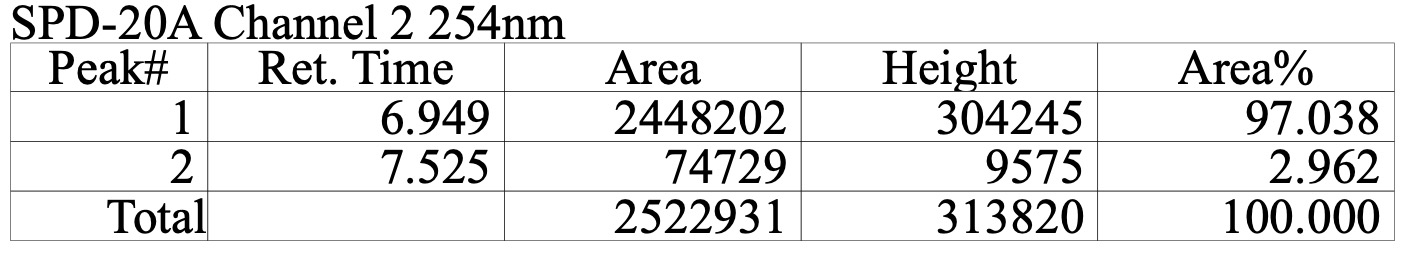


**(S)-3w**


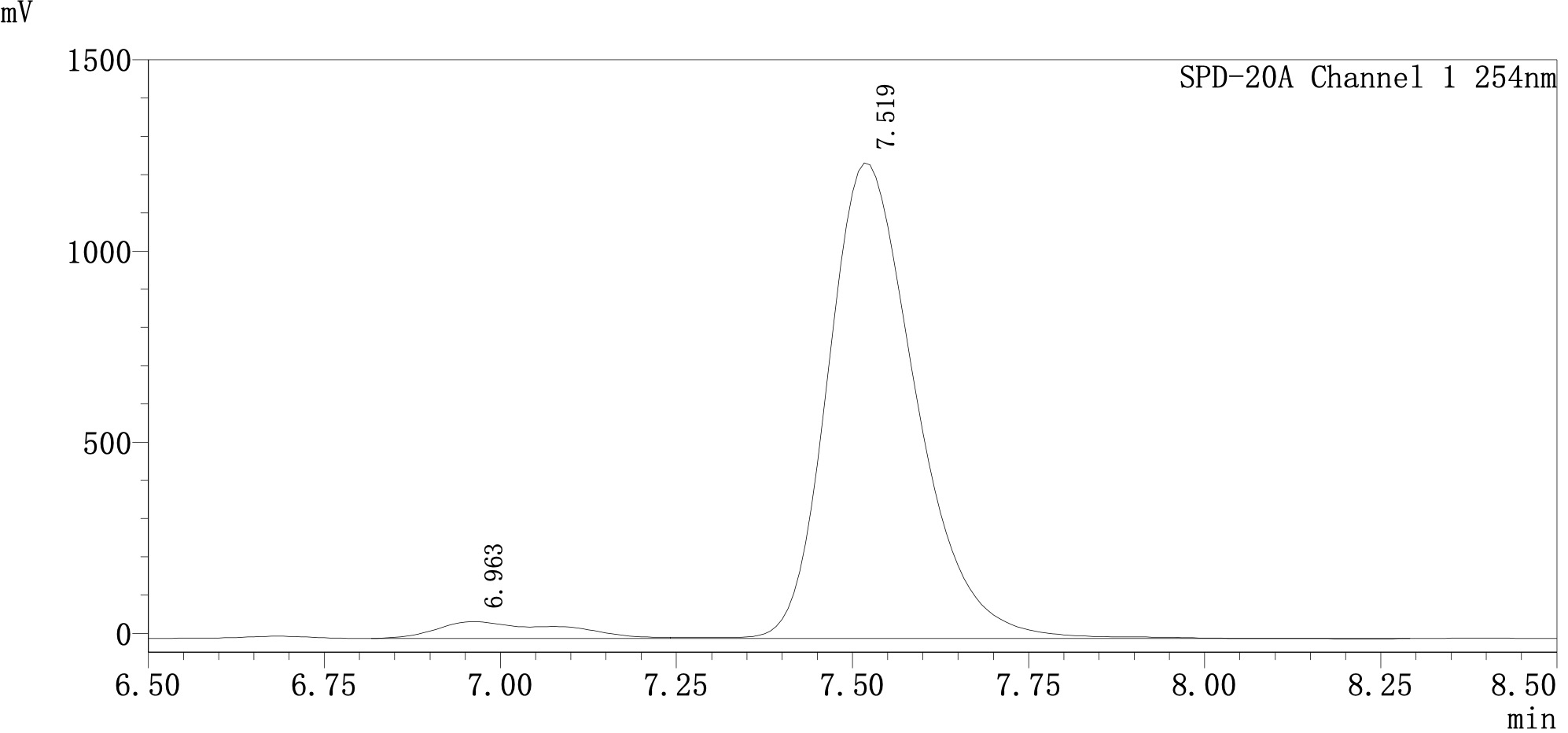


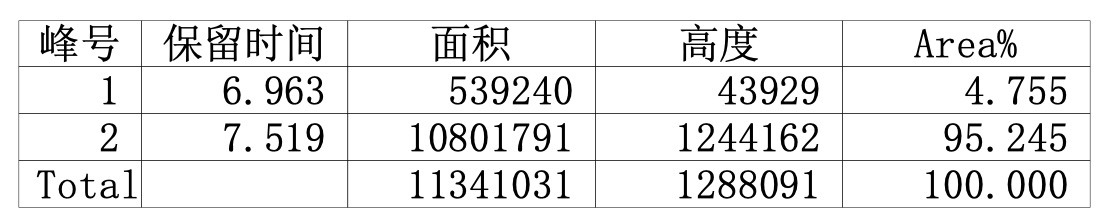


**Racemic 3x**


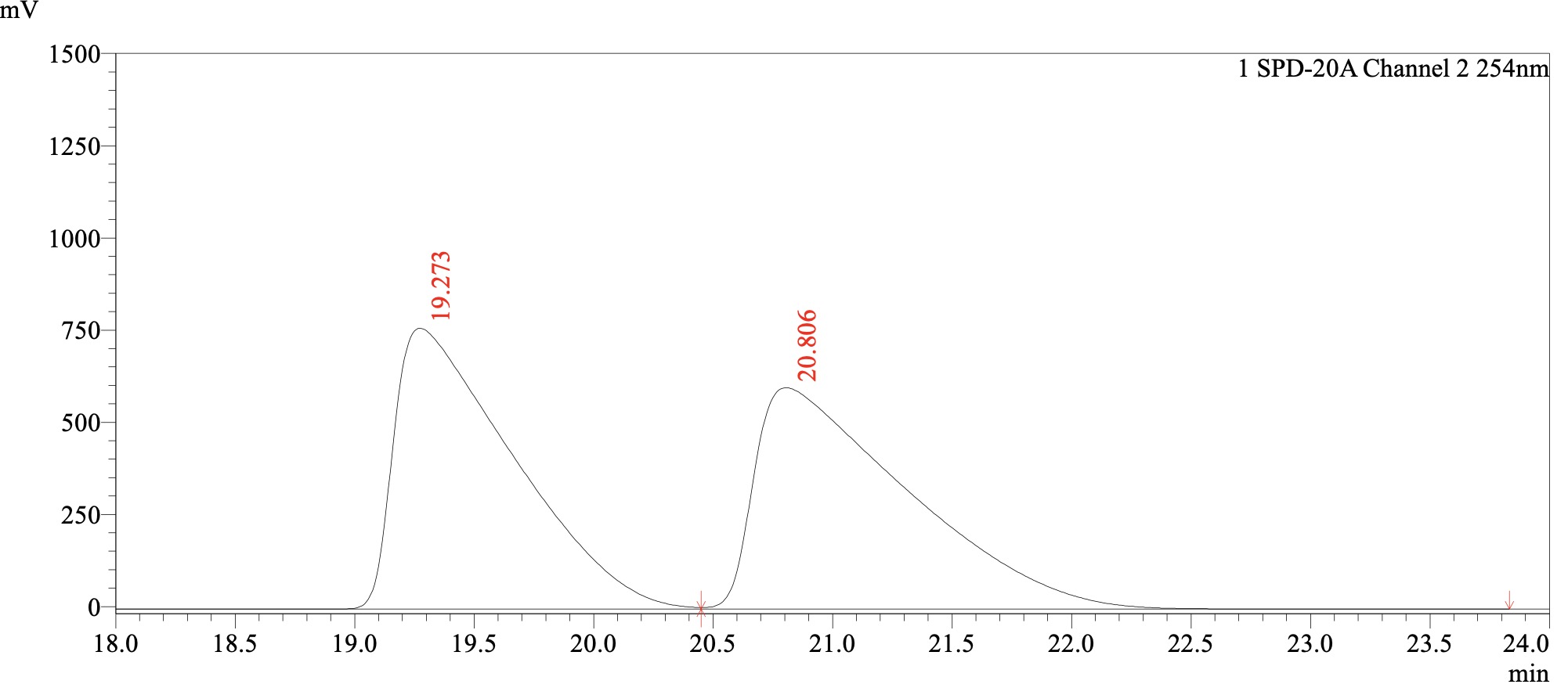


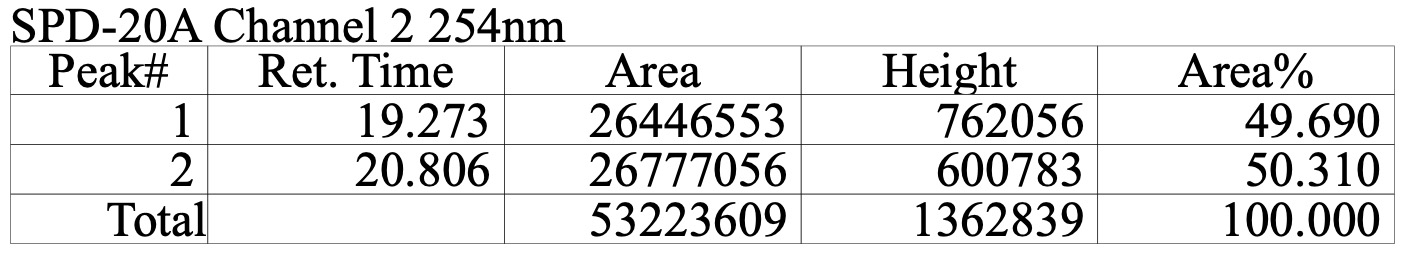


**(R)-3x**


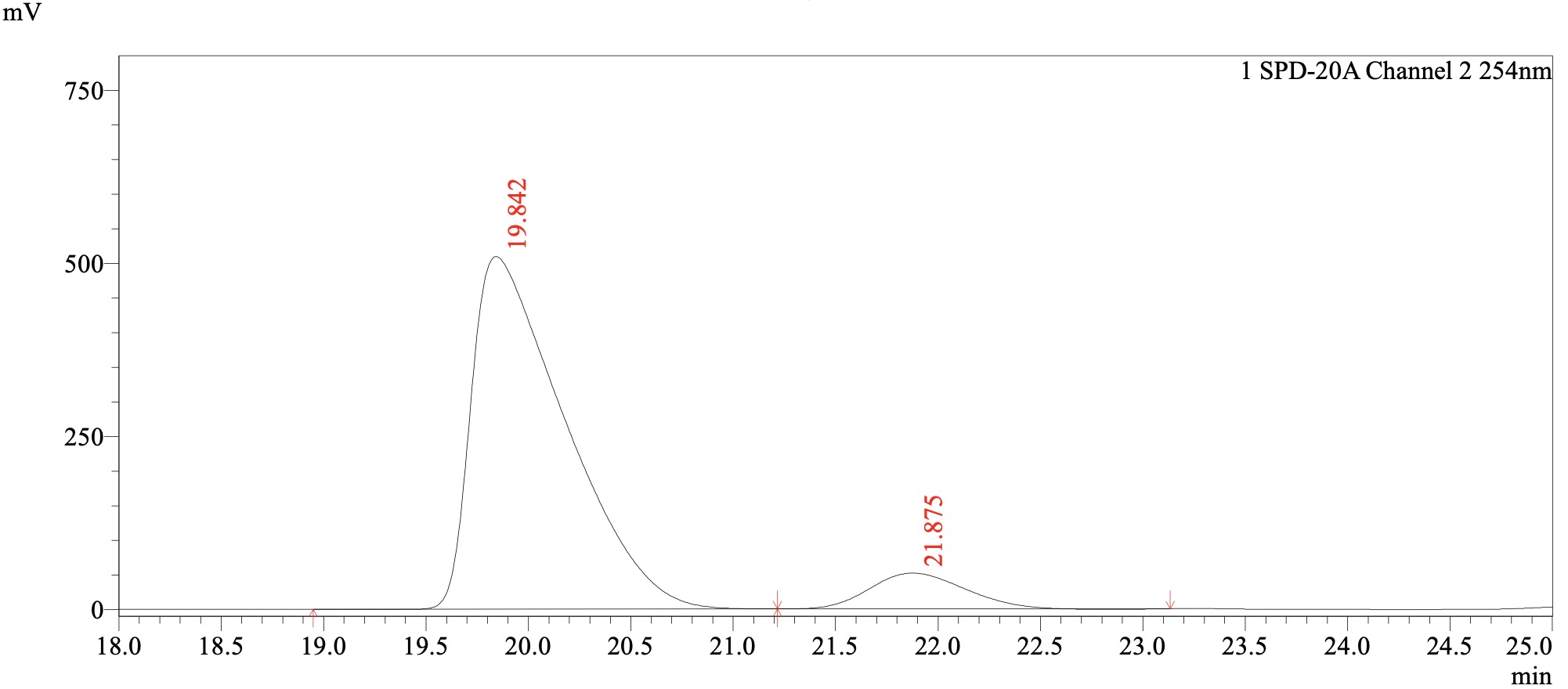


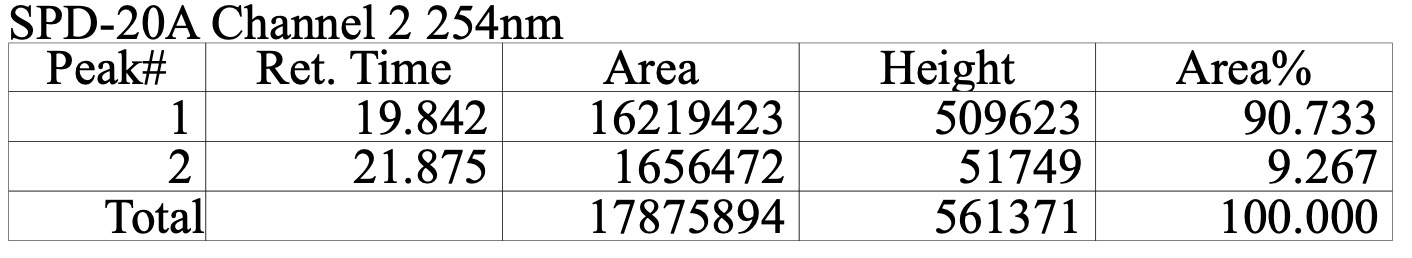


**Racemic 3y**


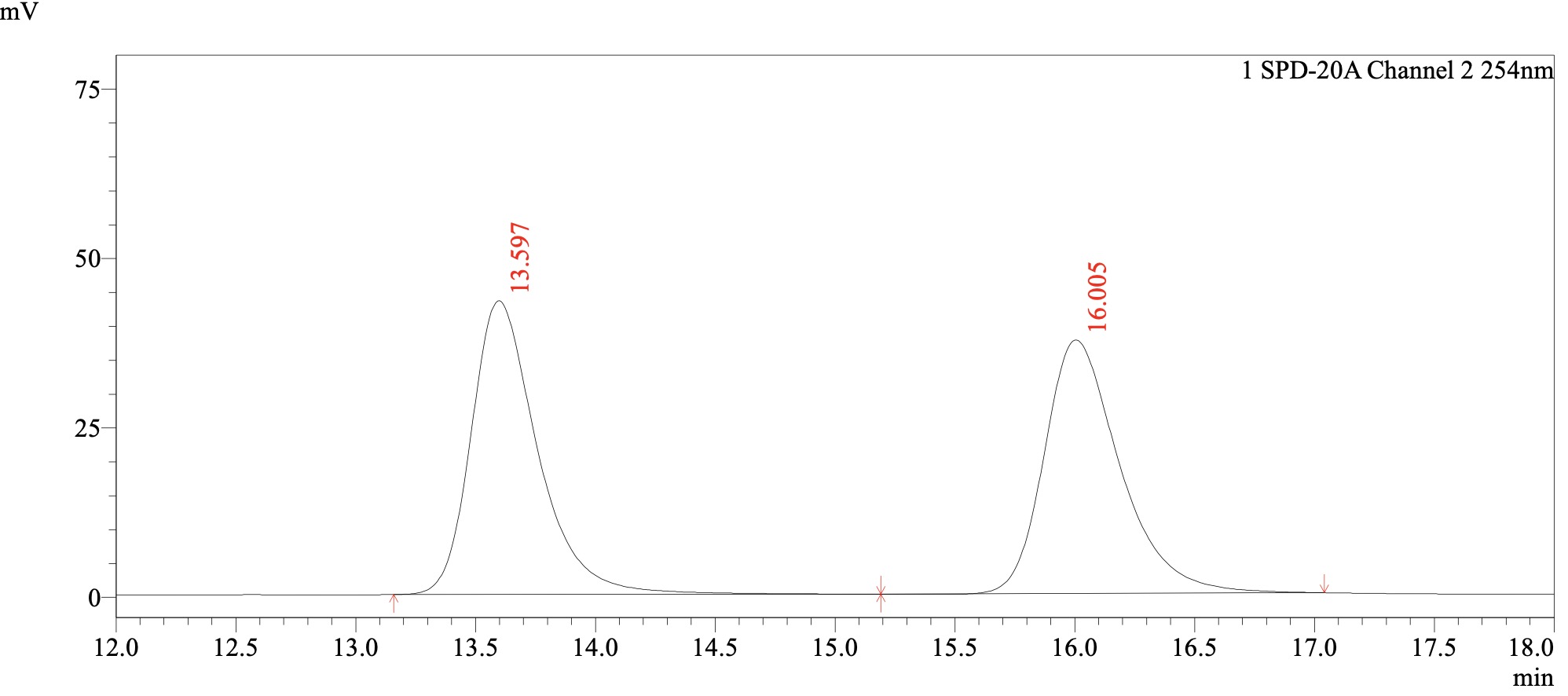


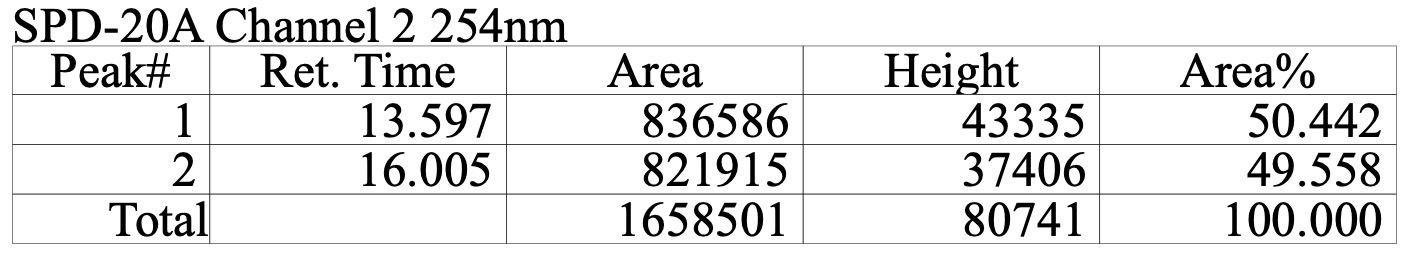


**(R)-3y**


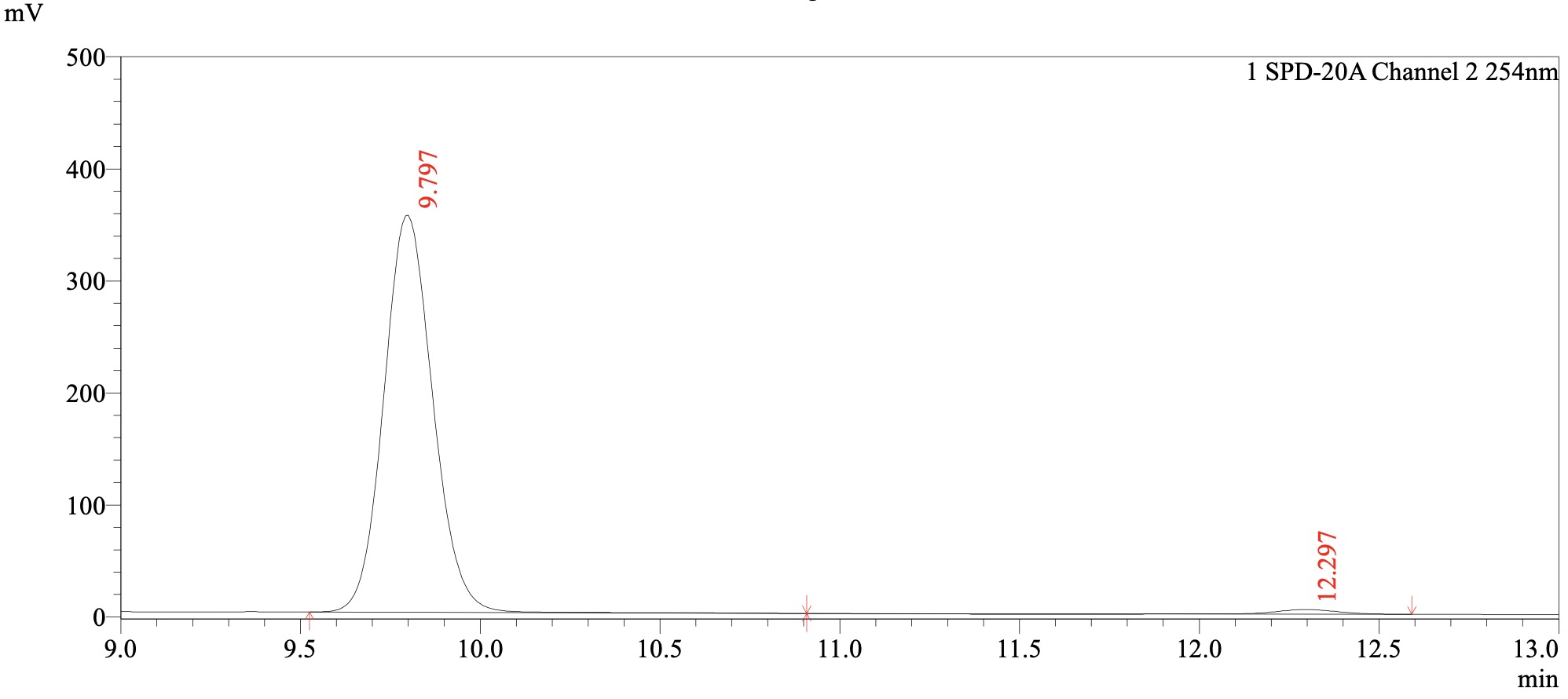


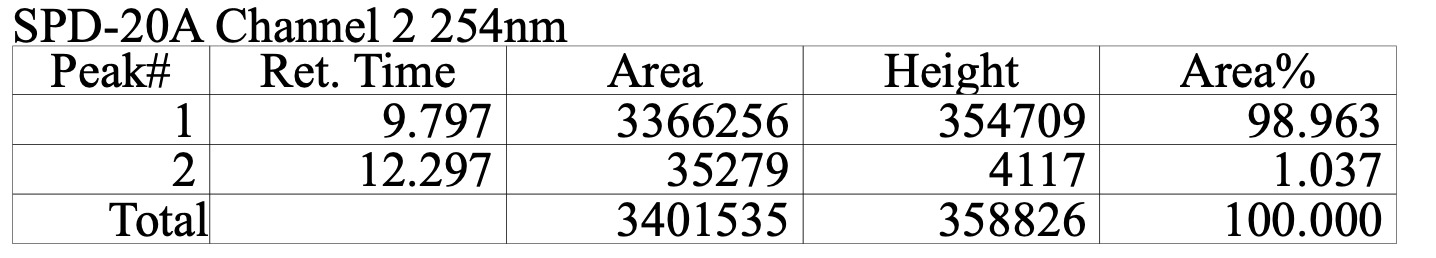


**Racemic 3z**


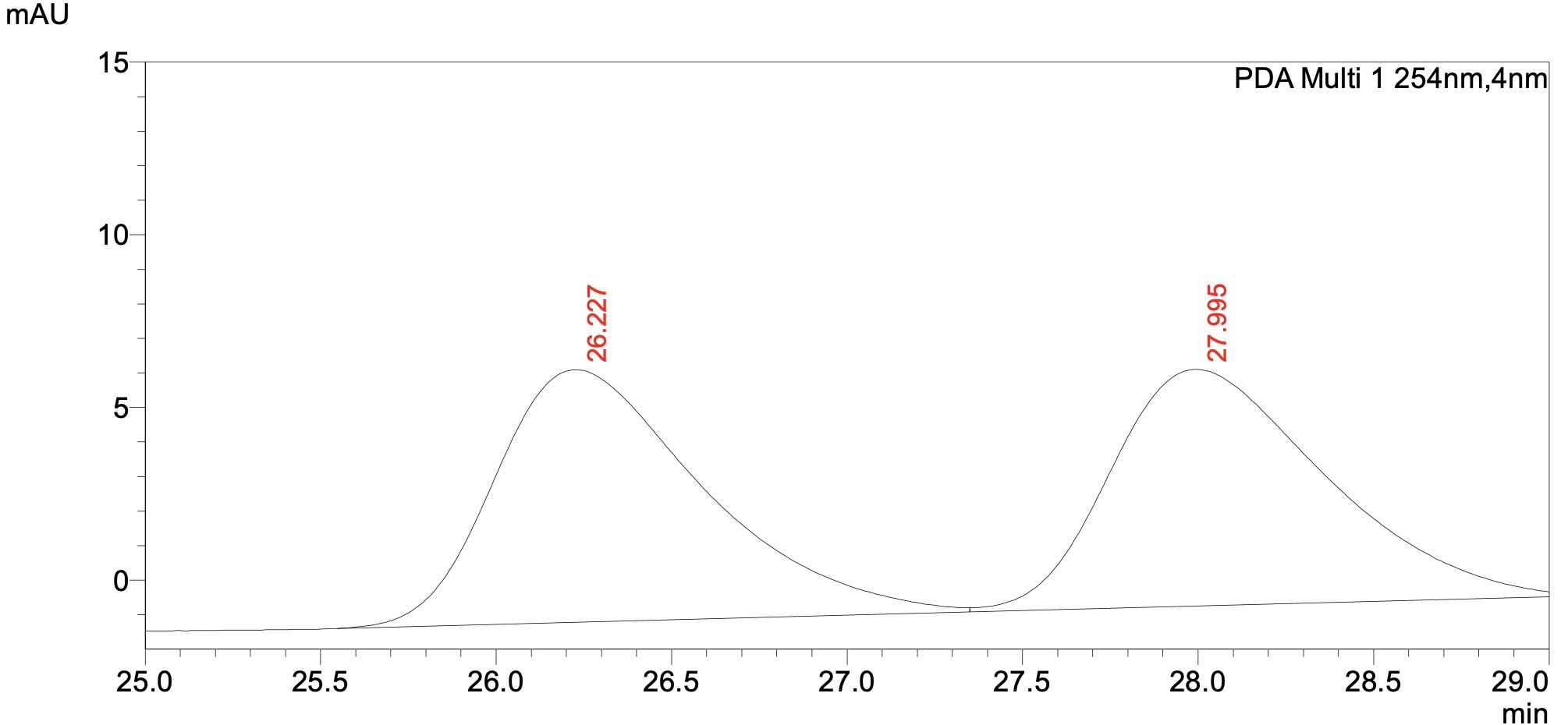


**(R)-3z**
